# Production and Structural Diversification of Withanolides by Aeroponic Cultivation of Plants of Solanaceae: Cytotoxic and Other Withanolides from Aeroponically Grown *Physalis coztomatl*

**DOI:** 10.3390/molecules27030909

**Published:** 2022-01-28

**Authors:** Ya-Ming Xu, E. M. Kithsiri Wijeratne, Manping X. Liu, Lijiang Xuan, Wenqiong Wang, A. A. Leslie Gunatilaka

**Affiliations:** 1 Southwest Center for Natural Products Research, School of Natural Resources and the Environment, College of Agriculture and Life Sciences, University of Arizona, 250 E. Valencia Road, Tucson, AZ 85706, USA; yamingx@arizona.edu (Y.-M.X.); Kithsiri@cals.arizona.edu (E.M.K.W.); manpingliu033@gmail.com (M.X.L.); 2 State Key Laboratory of Drug Research, Shanghai Institute of Materia Medica, Chinese Academy of Sciences, 501 Haike Road, Zhangjiang Hi-Tech Park, Shanghai 201203, China; ljxuan@simm.ac.cn (L.X.); wenqiong1019@126.com (W.W.)

**Keywords:** plants of Solanaceae, aeroponic cultivation, *Physalis coztomatl*, withanolides, anticancer activity, prostate cancer

## Abstract

Withanolides constitute one of the most interesting classes of natural products due to their diversity of structures and biological activities. Our recent studies on withanolides obtained from plants of Solanaceae including *Withania somnifera* and a number of *Physalis* species grown under environmentally controlled aeroponic conditions suggested that this technique is a convenient, reproducible, and superior method for their production and structural diversification. Investigation of aeroponically grown *Physalis coztomatl* afforded 29 withanolides compared to a total of 13 obtained previously from the wild-crafted plant and included 12 new withanolides, physacoztolides I−M (**9**–**13**), 15*α*-acetoxy-28-hydroxyphysachenolide C (**14**), 28-oxophysachenolide C (**15**), and 28-hydroxyphysachenolide C (**16**), 5*α*-chloro-6*β*-hydroxy-5,6-dihydrophysachenolide D (**17**), 15*α*-acetoxy-5*α*-chloro-6β-hydroxy-5,6-dihydrophysachenolide D (**18**), 28-hydroxy-5*α*-chloro-6*β*-hydroxy-5,6-dihydrophysachenolide D (**19**), physachenolide A-5-methyl ether (**20**), and 17 known withanolides **3**–**5**, **8**, and **21**–**33**. The structures of **9**–**20** were elucidated by the analysis of their spectroscopic data and the known withanolides **3**–**5**, **8**, and **21**–**33** were identified by comparison of their spectroscopic data with those reported. Evaluation against a panel of prostate cancer (LNCaP, VCaP, DU-145, and PC-3) and renal carcinoma (ACHN) cell lines, and normal human foreskin fibroblast (WI-38) cells revealed that **8, 13**, **15**, and **17**–**19** had potent and selective activity for prostate cancer cell lines. Facile conversion of the 5,6-chlorohydrin **17** to its 5,6-epoxide **8** in cell culture medium used for the bioassay suggested that the cytotoxic activities observed for **17**–**19** may be due to in situ formation of their corresponding 5β,6β-epoxides, **8**, **27**, and **28**.

## 1. Introduction

Withanolides, a class of polyoxygenated steroidal lactones frequently encountered in plants of the family Solanaceae [1], are known to exhibit a variety of biological activities including cytotoxic, anti-feedant, insecticidal, trypanocidal, leishmanicidal, antimicrobial, anti-inflammatory, phytotoxic, cholinesterase inhibitory and immune-regulatory activities, and the effects on neurite outgrowth and synaptic reconstruction [2,3]. Despite these interesting and diverse biological activities, studies on withanolides have not proceeded beyond preliminary evaluation in cellular and biochemical assays, arguably due to their supply issues as is the case with many biologically active natural products (NPs), including Taxol^®^ [4]. Traditionally, plant-based NPs are obtained from plant biomass produced by conventional cultivation in soil and/or wild-crafting. Both these methods are susceptible to unanticipated environmental catastrophes and also suffer from disadvantages as the former is labor intensive and is costly in land and water usage and the latter may lead to non-sustainable excessive harvesting causing ecological damage to their natural environment. To overcome these disadvantages, we have investigated the use of an environmentally controlled aeroponic cultivation technique for the production of biomass of some plants of Solanaceae and their constituent withanolides.

**Figure 1:**
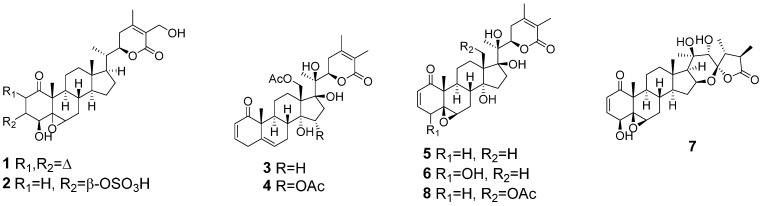
Major withanolides (**1**–**8**) encountered in some aeroponically grown plants of Solanaceae.

Possible application of soil-less aeroponic and hydroponic cultivation systems in controlled environments for research and commercial scale production of plant biomass has been recognized for nearly two decades [5]. Compared with the well-known hydroponic technique which utilizes a nutrient solution flowing over or in constant contact with the plant roots, the aeroponic cultivation technique constitutes a modified hydroponic technique in which the nutrient medium is intermittently sprayed on the roots which are suspended in air enclosed in an aeroponic chamber [6] (for details, see Supplementary Materials Figure S1). It has been suggested that out of the two techniques, aeroponic is the optimum technique for growing intact plants [7], especially because it allows control of root zone temperature, nutrition, moisture, and gas exchange while at the same time reducing disease occurrence and transmission [8]. It is known that cultivation of medicinal plants under aeroponic conditions provides opportunities for biomass production and improving the quality, purity, and consistency of the material produced, thus overcoming some of the major disadvantages of wild-harvesting and conventional soil and hydroponic cultivation techniques [9]. In addition, aeroponic cultivation under environmentally controlled conditions has been estimated to save the labor cost considerably, water usage by 98%, fertilizer usage by 60%, pesticide and herbicides usage by 100% and increase plant yield by 45% to 75% than either hydroponic or geoponic (soil-based) systems [10]. Although aeroponic systems have been used for the production of food crops [11,12,13,14] and medicinal plants [9], and its potential for improving production of high-value phyto-pharmaceuticals has been suggested [8], to the best of our knowledge this technique has not been exploited for the production of plant secondary metabolites prior to our recent report on the efficient production of a potential pro-drug of withaferin A (**1**), namely 2,3-dihydrowithaferin A-3*β*-O-sulfate (**2**) (Figure 1), by aeroponically grown *Withania somnifera* (Solanaceae) [15,16].

Studies with *W. somnifera* and another Solanaceae species, *Physalis crassifolia*, also suggested that the plant growth rate, yields of biomass and major withanolides, and the ability to produce structurally-diversified withanolides were improved when cultivated using the aeroponic technique compared to soil cultivation under identical controlled-environmental conditions. Thus, aeroponic cultivation of *W. somnifera* resulted in the production of two unusual withanolides, 3*α*-(uracil-1-yl)-2,3-dihydrowithaferin A and 3*β*-(adenin-9-yl)-2,3-dihydrowithaferin, in addition to withaferin A (**1**), 2,3-dihydrowithaferin A-3*β*-*O*-sulfate (**2**), and ten other known withanolides [17] (see Supplementary Data, Figure S2). Significantly, the aeroponic cultivation of *P. crassifolia* produced eleven new 17*β*-hydroxywithanolides (17-BHWs) [18] together with 15*α*-acetoxyphysachenolide D, 15*α*-acetoxy-28-hydroxyphysachenolide D, 18-acetoxy-17-epi-withanolide K, and physachenolide D encountered in the wild-crafted/soil-grown plant [19] (see Supplementary Data, Figure S3). We have also had notable success with the aeroponic technique in cultivating other plants of the Solanaceae, such as *P. peruviana* [20], *P. philadelphica* [21], *P. acutifolia* [22], and *P. coztomatl* (this study) and isolating and characterizing over 33 new withanolides, some with promising activities related to their potential use as anticancer agents. Depicted in Table 1 are some Solanaceae plant species grown using the aeroponic technique and comparison of the number of withanolides produced and the % yields of major withanolides (**1**–**8**, Figure 1) obtained from the biomass of the aeroponically grown plants with the wild-harvested and/or soil-cultivated plants.

We have previously demonstrated that unlike the most extensively studied cytotoxic withanolides including withaferin A (**1**) with a *β*-oriented side chain, 17*β*-hydroxywithanolides (17-BHWs) such as physachenolide C (**8**), with an *α*-oriented side chain, selectively inhibited prostate cancer (PC) cell lines at nanoMolar concentrations without affecting many other cancer cell lines and normal human fibroblast cells [18,19,20,21]. Our recent studies suggested that the 17-BHW, physachenolide C (**8**), was also capable of potentiating immunotherapy of renal carcinoma and melanoma, when used in combination with the immune adjuvants, tumor necrosis factor-*α* related apoptosis-inducing ligand (TRAIL) and the *ds*-RNA mimetic, poly I:C [29,30,31,32,33], respectively. Physachenolide C (**7**) was also shown to induce complete regression of established murine melanoma tumors via apoptosis and cell cycle arrest [34]. Thus, it was of interest to investigate withanolides belonging to different structural types for their potential anticancer activity. Herein we report the isolation and identification of 12 new (**9**–**20**) and 17 known (**3**–**5**, **8**, and **21**–**33**) withanolides from aeroponically grown *Physalis coztomatl* Moc. and Sessé ex Dunal (Solanaceae) and in vitro evaluation of withanolides (**3**–**5**, and **8**–**33**) against a panel of prostate cancer and renal carcinoma cell lines, and normal human fibroblast cells. Previous studies on *P. coztomatl*, a plant native to South America, has resulted in the isolation of 13 withanolides in two independent studies [27,28], including six 17-BHWs (**3**, **4**, **25**, **26**, **30**, and **32**), all of which were also encountered in the biomass obtained from aeroponic cultivation of this plant.

## 2. Results and Discussion

### 2.1. Isolation and Structure Elucidation

**Figure 2:**
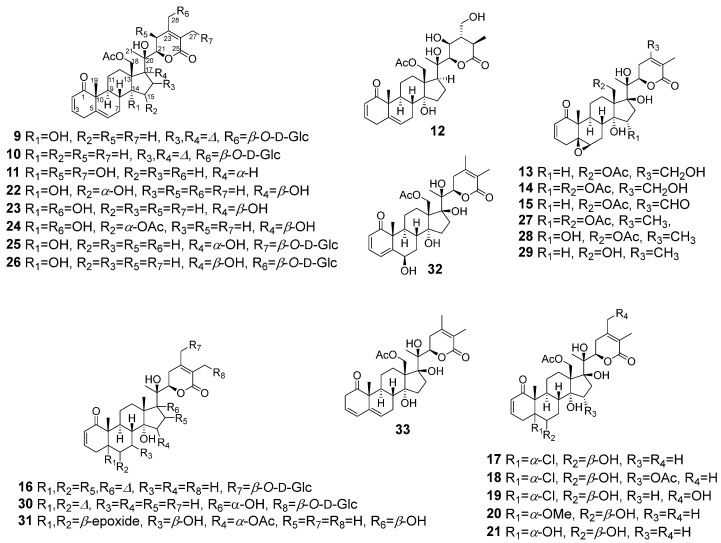
Structures of withanolides **9**–**33** encountered in aeroponically grown *P. coztomatl* (for structures of other withanolides (**3**–**5** and **8**) encountered, see Figure 1).

A MeOH extract of the aerial parts of aeroponically grown *P. coztomatl* on fractionation by solvent–solvent partitioning, and column chromatograpy (CC) employing HP-20SS, C_18_ RP, and silica gel followed by purification using prep TLC and HPLC afforded withanolides **3**–**5**, **8** (Figure 1), and **9**–**33** (Figure 2).

Compounds **9** and **10** were identified as withanolide glycosides from their characteristic NMR data and were named as physacostolides I and J, respectively. The molecular formula of **9** was determined to be C_36_H_48_O_13_ by a combination of its HRESIMS and NMR data, suggesting thirteen degrees of unsaturation. The ^1^H NMR spectrum of **9** (Table 2) showed three singlet methyl signals typical of withanolides [*δ*_H_ 1.22 (s), 1.28 (s), 1.85 (s)], a signal for an acetate group [*δ*_H_ 2.07 (s)], four olefinic signals [*δ*_H_ 5.57 (br s, H-6), 5.82 (br s, H-16), 5.83 (br d, *J* = 9.6 Hz, H-2), 6.77 (br d, *J* = 9.6 Hz, H-3)], three oxygenated methylenes [*δ*_H_ 4.59 (d, *J* = 10.4 Hz, H-18), 3.99 (d, *J* = 10.4 Hz, H-18), 4.46 (m) and 3.81 (m, Ha-6′); 3.82 (m, Hb-6′)], and an anomeric proton of a sugar moiety [*δ*_H_ 4.27 (d, *J* = 6.0 Hz)]. The ^13^C NMR spectrum of **9** (Table 2) displayed thirty-six carbon signals including six signals typical of a glucoside moiety (*δ*_C_ 102.5, 73.3, 75.8, 69.4, 76.4, and 61.2), three methyls (*δ*_C_ 12.3, 18.7, and 24.8), eight olefinic carbons (*δ*_C_ 151.1, 148.2, 145.7, 135.2, 127.8, 126.7, 124.9, and 123.4), two ester carbonyls (*δ*_C_ 165.9 and 171.4), and a conjugated ketone carbonyl (*δ*_C_ 204.4). The absence of a signal due to an oxygenatied carbon around 87 ppm in the ^13^C NMR spectrum indicated that C-17 is non-oxygenated [18]. Thus, **9** was suspected to contain a 16,17-double bond and this was confirmed by the HMBC correlations (see Supplementary Data, Figures S7 and S60) of H_3_-21 [*δ*_H_ 1.28 (s)]/C-17(*δ*_C_ 151.1), H_3_-21/C-20 (*δ*_C_ 74.4), and H_3_-21/C-22 (*δ*_C_ 80.8). The NMR chemical shifts (*δ*_H_ 1.85; *δ*_C_ 12.3) of one of the methyl group suggested that it was attached to an olefinic carbon. This was confirmed to be C-27 methyl group by the HMBC correlations of H_3_-27 [*δ*_H_ 1.85 (s)]/C-26 (*δ*_C_ 165.9), H_3_-27/C-25 (*δ*_C_ 123.4), and H_3_-27/C-24 (*δ*_C_ 148.2). The absence of a signal due to C-28 methyl group and the presence of an oxygenated CH_2_ group suggested the possible attachment of an *O*-glucosyl moiety to C-28 [18]. The HMBC correlations of H_3_-19 [*δ*_H_ 1.22 (s)]/C-1 (*δ*_C_ 204.4), H_3_-27 [*δ*_H_ 1.85 (s)]/C-26 (*δ*_C_ 165.9), H_3_-27/C-25 (*δ*_C_ 123.4), and H_3_-27/C-24 (*δ*_C_ 148.2) (see Supplementary Data, Figure S60) precluded oxygenation of C-19 and C-27 methyl groups. The identity of the sugar moiety was confirmed to be a d-glucose by the acid hydrolysis of **9** to afford a sugar with positive specific optical rotation. The ECD spectrum of **9** showed positive cotton effect at 256 nm (see Supplementary Data, Figure S59), suggesting the *R* configuration of C-22 [35,36]. Based on the foregoing data, the structure of physacoztolide I was determined as (20*S*,22*R*)-18-acetoxy-28*β*-d-*O*-glucopyranosyl-14*α*,20*β*-dihydroxy-1-oxo-witha-2,5,16,24-tetraenolide (**9**).

The molecular formula of physacostolide J (**10**) was determined to be C_36_H_48_O_12_ from its HRESIMS and NMR data. The ^1^H NMR data of **10** (Table 1) were similar to those of **9**, and the difference in molecular formulae between **10** (C_36_H_48_O_12_) and **9** (C_36_H_48_O_13_) indicated that **10** may be a deoxygenated analogue of **9**. The assignment of the ^13^C NMR spectrum (Table 2) by HSQC and HMBC data (see Supplementary Data, Figures S11 and S60) also revealed the similarities between **9** and **10**. The major difference in the NMR data was found to be the absence of oxymethine group at *δ*_C_ 83.4, which was assigned to C-14 in **9**. Instead, **10** showed the presence of a methine group (*δ*_C_ 57.6). This was confirmed by the up-field chemical shifts (Δ*δ*_C_: −3.2 ppm for C-8, −5.7 ppm for C-13, and −9.3 ppm for C-15) of carbons located *β* to C-14 in **10** when compared with those of **9** (Table 2). Acid hydrolysis of **10** gave d-glucose. The ECD spectrum of **10** showed a positive cotton effect at 257 nm (see Supplementary Data, Figure S59), suggesting the *R* configuration of C-22 [35,36]. Thus, the structure of physacoztolide J was determined as (20*S*,22*R*)-18-acetoxy-28*β*-d-*O*-glucopyranosyl-20*β*-hydroxy-1-oxo-witha-2,5,16,24-tetraenolide (**10**).

The HRESIMS, ^1^H and ^13^C NMR data of physacoztolide K (**11**) were consistent with the molecular formula, C_30_H_40_O_9_. The ^1^H NMR data of **11** (Table 2) exhibited signals typical of a withanolide consisting of three olefinic protons [*δ*_H_ 5.57 (brs, H-6), 5.84 (dd, *J* = 10.0, 2.0 Hz, H-2), and 6.78 (ddd, *J* = 10.0, 4.8, 2.4 Hz, H-3)], suggesting the presence of 2,3-en-1-one and 5,6-double bond moieties similar to physachenolide D (**3**) [18]. The ^1^H NMR signals due to four methyls including an acetate group [*δ*_H_ 1.20 (s), 1.43 (s), 2.06 (s), and 2.07 (s)] suggested that two of the methyl groups of the withanolide skeleton are substituted. This was confirmed by the presence of two oxygenated methylene signals [*δ*_H_ 4.34 (2H, s), 3.98 (1H, d, *J* = 11.6 Hz) and 4.40 (1H, d, *J* = 11.6 Hz)]. The two low-field oxygenated methines [*δ*_H_ 4.23 (d, *J* = 7.8 Hz) and 4.35 (d, *J* = 7.8 Hz)] in the ^1^H NMR spectrum of **11** which coupled with each other suggested that C-23 is oxygenated. The ^13^C NMR spectrum of **11** (Table 2) exhibited signals for five oxygenated carbons including two methylenes, two methines, and one non-protonated carbon, implying that C-14 or C-17 in **11** are not oxygenated. The absence of any oxygen-bearing substituents at C-17 was confirmed by the HMBC correlation of H_3_-21 [*δ*_H_ 1.43 (s)]/C-17 (*δ*_C_ 49.3) (see Supplementary Data, Figure S60), and the triplet for H-17 [*δ*_H_ 2.70 (t, *J* = 9.2 Hz)] established the configuration of the side chain at C-17 to be *β* [37]. This was further supported by the NOE correlations of H_3_-21/H-17*α* and H_3_-21/H-12*β* (see Supplementary Data, Figure S61). The presence of oxygenated substituents at C-23 and C-27 of **11** was apparent from the HMBC correlations of H_3_-28 [*δ*_H_ 2.06 (s)]/C-23 (*δ*_C_ 66.9) and H_2_-27 [*δ*_H_ 4.34 (s)]/C-26 (*δ*_C_ 164.6) (see Supplementary Data, Figures S15 and S60). The large coupling constant observed for H-22/H-23 (*J* = 7.8 Hz) was consistent with 23*β*-hydroxyphysacoztolide E-type sub-structure [18], suggesting the orientation of OH-23 as *β*. The positive Cotton Effect at 256 nm in its ECD spectrum (see Supplementary Data, Figure S59) established the 22*R* configuration [35,36]. On the basis of the foregoing evidence, the structure of physacoztolide K was elucidated as (17*R*,20*S*,22*R*)-18-acetoxy-14*α*,20*β*,23*β*,27-tetrahydroxy-1-oxo-witha-2,5,24-trienolide (**11**).

The molecular formula of physacoztolide L (**12**) was determined to be C_30_H_42_O_9_ from its HRESIMS and NMR data. The ^1^H and ^13^C NMR data (Table 2) suggested that the ring E of **12** is saturated unlike the other withanolides found to co-occur in this plant which contained an unsaturated E-ring. The ^1^H NMR spectrum of **12** (Table 2) exhibited signals due to four methyls including an acetyl and a secondary methyl [*δ*_H_ 1.22 (s), 1.28 (d, *J* = 6.8 Hz), 1.40 (s,), and 2.14 (s,)], two oxygenated methylenes [*δ*_H_ 4.89 (d, *J* = 11.6 Hz)/3.70 (d, *J* = 11.6 Hz) and 3.83 (1H, dd, *J* = 11.2, 2.0 Hz)/3.64 (1H, dd, *J* = 11.2, 7.6 Hz)], three olefinic protons [*δ*_H_ 5.57 (d, *J* = 5.6 Hz, H-6), 5.85 (dd, *J* = 10.0, 2.0 Hz, H-2), and 6.76 (ddd, *J* = 10.0, 4.8, 2.4 Hz, H-3)], and two oxygenated methines [*δ*_H_ 4.06 (dd, *J* = 8.4, 8.0 Hz, H-23), 3.60 (d, *J* = 8.4 Hz, H-22)]. The coupling between the two oxygenated methines suggested possible hydroxylation at C-23 [18]. The ^13^C NMR spectrum of **12** (Table 2) displayed thirty carbon signals including an acetyl group (*δ*_C_ 169.7 and 21.2). The assignment of ^13^C NMR spectrum with the help of HSQC and HMBC data suggested that C-17 (*δ*_C_ 49.9) is not oxygenated like in **11**, and ring E is saturated as indicated by the up-field shift of the carbonyl signal (*δ*_C_ 177.6) compared withanolides bearing an unsaturated E-ring *δ*-lactone [18]. The remaining ^13^C NMR signals [*δ*_C_ 75.9 (CH), 81.1 (CH), 51.0 (CH), and 37.0 (CH)] of the *δ*-lactone further confirmed that ring E is saturated. The presence of HMBC correlation of H_3_-27 [*δ*_H_ 1.28 (d, *J* = 6.8 Hz)]/C-26 (*δ*_C_ 177.6) (see Supplementary Data, Figures S21 and S60) confirmed that C-28 methyl is oxygenated to a CH_2_OH moiety. The ^1^H–^1^H COSY correlations of H-22/H-23, H-23/H-24, H-24/H_2_-28, and H_3_-27/H-25 (see Supplementary Data, Figures S22 and S60) together with the above data established the planer structure of ring E. The NOESY correlations of H_3_-21/H-23 and H-22/H23 (see Supplementary Data, Figures S23 and S61) suggested the *cis* configuration for H-22 and H-23, and hence the orientation of H-23 should be equatorial. The coupling constant (*J* = 8.0 Hz) between H-23 and H-24 was identical to that of 24,25-dihydro-23*β*,28-dihydroxywithanolide G [20], which established the *trans* configuration of H-23 and H-24. The irradiation of H_3_-27 showed an NOE with H-24, suggesting the *trans* configuration of CH_3_-27 and CH_2_OH-28. These data indicated that the gross structure of ring E of **12** is the same as that of 24,25-dihydro-23*β*,28-dihydroxywithanolide G, which was further supported by their almost identical ^13^C NMR chemical shifts for the carbons of the ring E moiety [20]. The absolute configuration of C-22 was determined as *R* by the positive Cotton effect at 256 nm in its ECD spectrum [28] (see Supplementary Data, Figure S59). The appearance of H-17 as a triplet [*δ*_H_ 2.73 (t, *J* = 9.7 Hz)] in its ^1^H NMR spectrum established the configuration of the side chain at C-17 as *β* [37]. Thus, the structure of physacoztolide L was identified as (17*S*,20*R*,22*R*,24*S*,25*R*)-18-acetoxy-14*α*,20*β*,23*β*,28-tetrahydroxy-1-oxo-witha-2,5-dienolide (**12**).

Withanolides **13**–**15** were found to contain a 5*β*,6*β*-epoxide moiety as indicated by their ^1^H NMR spectra having a typical broad singlet or a doublet with a small coupling constant for H-6*α* [*δ*_H_ 3.23 (br s) for **13**, 3.20 (d, *J* = 2.0 Hz) for **14**, and 3.15 (br s) for **15**] and from their ^13^C NMR signals for C-5 and C-6 [*δ*_C_ 63.4 (C-5) and 65.0 (C-6) for **13**, 63.2 (C-5) and 65.0 (C-6) for **14**, and 62.0 (C-5), and 63.9 (C-6) for **14**]. Their ECD spectra (see Supplementary Data, Figure S59) were almost identical and showed positive cotton effects at 258 nm and 341 nm, suggesting the *cis*-linkage of rings A/B and the 22*R* configuration [35,36].

Based on its HRESIMS and NMR data, withanolide **13** was determined to have the molecular formula C_30_H_40_O_10_ indicating eleven degrees of unsaturation. The ^1^H NMR and ^13^C NMR data (Table 3) suggested that its structure is closely related to that of physachenolide C (**8**), the major withanolide of this plant. These NMR data, together with its molecular formula, indicated that **13** contained one oxygen atom more than that of physachenolide C (**8**). Comparison of the ^1^H and ^13^C NMR data (Table 3) of **13** with those of **8** [38] revealed that CH_3_-27 or CH_3_-28 in **8** was oxygenated to a CH_2_OH group [*δ*_H_ 4.36 (d, *J* = 13.6 Hz) and 4.24 (d, *J* = 13.6 Hz); *δ*_C_ 61.9] in **13**. The presence of CH_2_OH-18 in **13** was confirmed by the HMBC correlations of H_3_-27 [*δ*_H_ 1.88 (s)]/C-26 (*δ*_C_ 169.1) and H_2_-28/C-23 (*δ*_C_ 30.3) (see Supplementary Data, Figures S27 and S60). The ECD spectrum of **13** showed a positive cotton effect at 256 nm (see Supplementary Data, Figure S59), suggesting the *R* configuration of C-22 [35,36]. Thus, withanolide **13** was identified as 28-hydroxyphysachenolide C [(20*S*,22*R*)-18-acetoxy-5*β*,6*β*-epoxy-14*α*,17*β*,20*β*,28-tetrahydroxy-1-oxo-witha-2,24-dienolide].

The molecular formula of **14** was established as C_32_H_42_O_12_ by its HRESIMS and NMR data. Careful analysis of ^1^H NMR and ^13^C NMR spectra of **14** (Table 3) suggested that it could be an acetoxy analogue of 28-hydroxyphysachenolide C (**13**) or an oxygenated analogue of 15*α*-acetoxyphysachenolide C (**27**) [29]. Comparison of the NMR data of **14** with those of **13** and **27** confirmed that the signals due to the rings A–D of **14** were identical with those of **27** [18], and the signals of the side chain including ring E of **14** were same as those of **13** suggesting that it could be 15*α*-acetoxy analogue of 28-hydroxyphysachenolide C. The structure was further supported by the HMBC correlations of H_2_-28 (*δ*_H_ 4.27 and 4.20)/C-23 (*δ*_C_ 30.3), H_2_-28/C-25 (*δ*_C_ 122.4), and H_3_-27 (*δ*_H_ 1.87)/C-26 (*δ*_C_ 169.0) (see Supplementary Data, Figures S31 and S60). The positive Cotton effect at 258 nm (see Supplementary Data, Figure S59) in its ECD spectrum established the *R* configuration for C-22 of **14** [35,36]. Thus, the structure of this withanolide was elucidated as 15*α*-acetoxy-28-hydroxyphysachenolide C [(20*S*,22*R*)-15*α*,18-diacetoxy-5*β*,6*β*-epoxy-14*α*,17*β*,20*β*,28-tetrahydroxy -1-oxo-witha-2,24-dienolide] (**14**).

The molecular formula of withanolide **15** was determined to be C_30_H_38_O_10_ based on its HRESIMS and NMR data, suggesting twelve degrees of unsaturation. The analysis of the ^1^H NMR and ^13^C NMR spectra (Table 3) with the help of HSQC and HMBC data revealed that the signals of rings A, B, C, and D of **15** were similar to those of 28-hydroxyphysachenolide C (**13**), except for those of ring E. Comparison of the molecular formula of **15** (C_30_H_38_O_10_) with that of **13** (C_30_H_40_O_10_) indicated that **15** contains two protons fewer than **13**. The presence of a low-field proton signal at 10.30 ppm in the ^1^H NMR spectrum and a signal at 190.2 ppm in the ^13^C NMR spectrum of **15** suggested that the CH_2_OH group attached to the ring E of **13** has undergone oxidation to a CHO group in **15**. The HMBC correlations of H_3_-27 [*δ*_H_ 2.35 (s)]/C-26 (*δ*_C_ 165.6) and H-28 [*δ*_H_ 10.30 (s)]/C-23 (*δ*_C_ 24.5) (see Supplementary Data, Figures S35 and S60) located this CHO to be at C-28. The positive Cotton effect at 258 nm (see Supplementary Data, Figure S59) in its ECD spectrum established the *R* configuration of C-22 [35,36]. The structure of **15** was thus established as 28-oxophysachenolide C [(20*S*,22*R*)-18-acetoxy-5*β*,6*β*-epoxy-14*α*,17*β*,20*β*-trihydroxy-1,28-dioxo-witha-2,24-dienolide].

Based on its HRESIMS and NMR data, withanolide **16** was determined to have the molecular formula C_30_H_40_O_10_. It was suspected to be a glucoside from its molecular formula, C_34_H_46_O_11_, and the presence of a signal due to an anomeric proton at *δ*_H_ 4.23 (d, *J* = 8.0 Hz) and the typical ^13^C NMR signals (*δ*_C_ 102.5, 73.3, 75.9, 69.8, 76.4, and 61.5) of the glucose moiety and was named physacoztolide M. The ^1^H NMR spectrum of **16** (Table 3) displayed signals due to four singlet methyl protons (*δ*_H_ 1.11, 1.18, 1.26, and 1.82), four olefinic protons [*δ*_H_ 5.79 (dd, *J* = 10.0, 2.0 Hz, H-2), 6.74 (ddd, *J* = 10.0, 4.8, 2.4 Hz, H-3), 5.57 (d, *J* = 5.6 Hz, H-6), and 5.78 (br s, H-16)], an oxygenated methine proton [*δ*_H_ 4.37 (t, *J* = 8.0 Hz, H-22)], and protons on an oxygenated methylene group [*δ*_H_ 4.42 (br s)]. The olefinic region of the ^1^H NMR spectrum of **16** was found to similar to that of physacoztolide I (**9**) (see above). The absence of a singlet methyl signal around 2.0 ppm in **16** suggested that it lacked the acetyl group present in **9**. These data suggested **16** has a similar skeleton as that of **9** and contained three double bonds at 2(3), 5(6), and 16(17) positions, and the AcOCH_2_ at C-13 in **9** was replaced by a CH_3_ group in **16**. The assignment of the ^13^C NMR spectrum of **16** (Table 3) with the help of the HSQC data (see Supplementary Data, Figure S38) and HMBC data (see Supplementary Data, Figures S39 and S60) and comparison of the ^13^C NMR data with those of **9** further confirmed that the AcO group at C-18 of **9** is replaced with a proton in **16**. The presence of the double bonds at 2(3) and 5(6), and 16(17) in **16** was further confirmed by the HMBC correlations of H_3_-19/C-5, H_3_-19/C-1, H-4/C-2, H-4/C-6, and H_3_-18/C-17, H_3_-21/C-17, H-16/C-20, respectively (see Supplementary Data, Figure S60). The long-range HMBC correlation between the anomeric proton of the glucose moiety and C-28 (*δ*_C_ 67.7) located the *O*-glycosyl moiety at C-28. The presence of the d-glucose moiety in **16** was further confirmed by the acid hydrolysis and the positive [*α*]_D_ obtained for the resulting sugar. The ECD spectrum of **16** showed a positive Cotton effect at 256 nm (see Supplementary Data, Figure S59) establishing the *R* configuration of C-22 [35,36]. Therefore, the structure of physacoztolide M was determined as (20*S*,22*R*)-28*β*-d-*O*-glucopyranosy-14*α*,20*β*-dihydroxy-1-oxo-witha-2,5,16,24-tetraenolide (**16**).

The HRESIMS data together with their ^1^H and ^13^C NMR spectra (Table 4) indicated that compounds **17**–**19** are chlorinated withanolides. The ^13^C NMR chemical shifts of C-5 and C-6 of these [*δ*_C_ 79.5 (C-5) and 75.0 (C-6) for **17**, 79.0 (C-5) and 74.6 (C-6) for **18**, and 82.3 (C-5), and 75.6 (C-6) for **19**] suggested that these are chlorohydrins containing 5-chloro-6-hydroxy substituents similar to withanolide C [39]. The ^13^C NMR data of **17**–**19** also showed some resemblance to those of physachenolide A (**21**) [38] suggesting their structural relationships (Table 4). Based on its HRMS and NMR data, **17** was determined to have the molecular formula C_30_H_41_ClO_9_. The ^1^H NMR spectrum of **17** (Table 4) displayed signals due to five singlet methyl protons (*δ*_H_ 1.36, 1.41, 1.88, 1.92, and 2.07), two olefinic protons characteristic of the 2,3-en-1-one moiety [*δ*_H_ 5.89 (dd, *J* = 10.0, 2.4 Hz, H-2), 6.62 (br dd, *J* = 10.0, 3.6 Hz, H-3)], protons of two oxygenated methines [*δ*_H_ 4.06 (br s, H-6), 4.90 (t, *J* = 8.4 Hz, H-22)], and an oxygenated methylene [*δ*_H_ 4.43 (s, H_2_-18)]. The ^13^C NMR spectrum of **17** (Table 4) assigned with the help of HSQC (see Supplementary Data, Figure S60) and HMBC (see Supplementary Data, Figures S43 and S60) data indicated that it has a structure closely related to that of physachenolide A (**21**) [38], but with minor chemical shift differences in the carbon signals of ring A/B moieties, especially C-1, C-4, C-5, and C-6 (see Table 4). These differences were suspected to be due to the presence of different substituents at C-5 and C-6 of **17** (chlorine and hydroxy) compared to **21** (dihydroxy). The ^13^C NMR chemical shifts of C-5, and C-6 of **17** [*δ*_C_ 79.5 (C-5) and 75.0 (C-6)] are consistent with those of withanolide C [*δ*_C_ 80.4 (C-5) and 74.7 (C-6)] [39] and physanicandrolide C [*δ*_C_ 80.9 (C-5) and 74.5 (C-6)] [40], suggesting the presence of 5α-Cl, 6β-OH substituents in **17**. The NOESY correlations of H-7*β* [*δ*_H_ 2.54 (m)]/H-6 [*δ*_H_ 4.06 (br s)] and H-7*α* [*δ*_H_ 1.52 (m)]/H-6 in **17** (see Supplementary Data, Figure S61) together with the appearance of H-6 as a broad singlet in its ^1^H NMR spectrum, confirmed the equatorial orientation of H-6 (i.e., *β*-orientation of 6-OH) as in physachenolide A (**21**) [38]. The ECD spectrum of **17** exhibited a positive Cotton effect at 252 nm and a negative cotton effect at 336 nm (see Supplementary Data, Figure S59) establishing the *R* configuration of C-22 [35,36], and *trans*-linkage of rings A/B (and hence *α* configuration of Cl-5) [39], respectively. Thus, the structure of **17** was elucidated as 5*α*-chloro-6*β*-hydroxy-5,6-dihydrophysachenolide D [(20*S*,22*R*)-18-acetoxy-5α-chloro-6*β*,14*α*,17*β*,20*β*-tetrahydroxy-1-oxo-witha-2,24-dienolide].

The molecular formula of compound **18** was determined to be C_32_H_43_ClO_11_ by the analysis of its HRESIMS and NMR data. The ^1^H NMR spectrum of **18** (Table 4) resembled that of 5*α*-chloro-6*β*-hydroxy-5,6-dihydrophysachenolide D (**17**), but exhibited an additional oxygenated methine signal [*δ*_H_ 5.20 (t, *J* = 8.8 Hz)], two acetyl methyl signals [*δ*_H_ 2.08 (s) and 2.09 (s)] besides the typical signals for H-2, H-3, H-22, H_2_-18, and the methyl groups. These data together with the difference in molecular formulae between **18** (C_32_H_43_ClO_11_) and **17** (C_30_H_41_ClO_9_) suggested that **18** may be an acetoxylated analogue of **17**. The ^13^C NMR spectrum of **18** (Table 4), assigned with the help of HSQC (see Supplementary Data, Figure S47) and HMBC data (see Supplementary Data, Figures S48 and S60), also suggested structural similarities between **18** and **17** except for the carbon signals of ring D, especially C-17 and C-15. The up-field shift of C-17 [Δ = *δ*(**10**) − *δ*(**9**) = −3.5 ppm, *γ*-effect] and down-field shift of C-16 [Δ = *δ*(**10**) − *δ*(**9**) = 4.6 ppm, *β*-effect] placed the acetoxy substituent at C-15. The orientation of this OAc group was determined to be *α* by the large coupling constant for H-15 [*δ*_H_ 5.20 (t, *J* = 8.8 Hz)] in its ^1^H NMR spectrum, which is identical with that of 15*α*-acetoxyphysachenolide D (**4**) [27]. The NOESY correlations of H-7*β* [*δ*_H_ 2.61 (m)]/H-6 [*δ*_H_ 3.97 (br s)] and H-7*α* [*δ*_H_ 1.54 (m)]/H-6 (see Supplementary Data, Figure S49), together with the appearance of H-6 as a broad singlet in its ^1^H NMR spectrum, confirmed the orientation of OH-6 of **18** as *β*, same as that of **17**. The ECD spectrum of **18** exhibited a positive Cotton effect at 252 nm and a negative cotton effect at 335 (see Supplementary Data, Figure S59) establishing the *R* configuration of C-22 and the *trans*-linkage of rings A/B [35,36]. On the basis of the foregoing evidence, the structure of withanolide **18** was elucidated as 15*α*-acetoxy-5*α*-chloro-6β-hydroxy-5,6-dihydrophysachenolide D [(20*S*,22*R*)-15*α*,18-diacetoxy-5α-chloro-6*β*,14*α*,17*β*,20*β*-tetrahydroxy-1-oxo-witha-2,24-dienolide].

The molecular formula of compound **19** was deduced to be C_30_H_41_ClO_10_ from its HRESIMS and NMR data. The ^1^H NMR spectrum of **19** (Table 4) displayed signals due protons of an *α*,*β*-unsaturated ketone moiety [*δ*_H_ 5.83 (dd, *J* = 10.0, 2.0 Hz, H-2), 6.75 (ddd, *J* = 10.0, 4.8, 2.0 Hz, H-3)], two oxygenated methylenes [*δ*_H_ 4.40 (s, H_2_-18), 4.39 (d, *J* = 14.0 Hz, H-28), 4.23 (d, *J* = 14.0 Hz, H-28)], two oxygenated methines [*δ*_H_ 3.93 (t, *J* = 2.9 Hz, H-6), 4.90 (m, H-22)], an acetate [2.12 (s)], and three singlet methyls [*δ*_H_ 1.38 (s), 1.40 (s), 1.89 (s)]. The ^13^C NMR spectrum of **19** (Table 4) assigned with the help of HSQC (see Supplementary Data, Figure S52) and HMBC data (see Supplementary Data, Figures S53 and S60) closely resembled that of 5*α*-chloro-6*β*-hydroxy-5,6-dihydrophysachenolide D (**17**), except for the signal assigned to C-28 which appeared at 62.0 ppm compared to that of **17** at 20.7 ppm. The above NMR data together with the difference in molecular formulae of **19** (C_30_H_41_ClO_10_) and **17** (C_30_H_41_ClO_9_) suggested that **19** could be a C-28 hydroxylated analogue of **17**. The presence of OH-28 was confirmed by the HMBC correlations of H_3_-27 [*δ*_H_ 1.89 (s)]/C-26 [*δ*_C_ 169.2], H_2_-28/C-25 [*δ*_C_ 122.5], and H_2_-28/C-23 [*δ*_C_ 30.3] (see Supplementary Data, Figure S60). The small coupling constant of H-6 [*δ*_H_ 3.93 (t, *J* = 2.9 Hz) indicated the orientation of OH-6 to be *β* similar to those of **17** and **18**. The ECD spectrum of **19** exhibited a positive Cotton effect at 255 nm and a negative Cotton effect at 336 nm (see Supplementary Data, Figure S59) establishing the *R* configuration of C-22 and the *trans*-linkage of rings A/B [35,36]. Based on foregoing evidence, the structure of this withanolide was elucidated as 28-hydroxy-5*α*-chloro-6*β*-hydroxy-5,6-dihydrophysachenolide D [(20*S*,22*R*)-18-acetoxy-5α-chloro-6*β*,14*α*,17*β*,20*β*,28-pentahydroxy-1-oxo-witha-2,24-dienolide] (**19**).

Withanolide **20** was determined to have the molecular formula, C_31_H_44_O_10_, based on its HRESIMS and NMR data. Its ^1^H NMR spectrum (Table 4) displayed signals typical of a withanolide, including those due to two olefinic protons of the 2,3-en-1-one moiety [*δ*_H_ 5.73 (dd, *J* = 10.0, 2.8 Hz, H-2), 6.48 (ddd, *J* = 10.0, 5.2, 2.0 Hz, H-3)], two oxygenated methines [*δ*_H_ 3.86 (brs, H-6), 4.84 (dd, *J* = 13.6, 3.2 Hz, H-22)], an oxygenated methylene [*δ*_H_ 4.37 (d, *J* = 11.2 Hz, H-18), 4.30 (d, *J* = 11.2 Hz, H-18)], an acetyl [*δ*_H_ 2.03 (s)], and four singlet methyls [*δ*_H_ 1.22 (s), 1.31 (s), 1.82 (s), 1.88 (s)]. A signal due to an OCH_3_ group [*δ*_H_ 2.93 (s)] rare in withanolides was also encountered, indicating that **20** is a methoxylated withanolide. Comparison of the ^13^C NMR data of **20** (Table 4) with those of physachenolide A (**21**) [38] revealed that the major differences are for C-4, C-5, and C-6 signals of ring A suggesting that one of the OH groups at C-5/C-6 of **21** has undergone methylation to a OCH_3_ group. It was also found that the ^13^C NMR signals due to C-4 and C-6 of **20** have shifted up-field by 7.6 and 5.2 ppm (*β*-effect), respectively, and C-5 has shifted down-field by 4.0 ppm (*α*-effect) compared to those of **21**, locating this OCH_3_ substituent at C-5. The presence of CH_3_O-5 in **20** was further supported by the HMBC correlation of CH_3_O/C-5 (see Supplementary Data, Figure S60). The orientation of OH-6 was determined as β by the small coupling constant of H-6 similar those of **17**–**19** and **21**. The ECD spectrum of **20** exhibited a positive Cotton effect at 257 nm and a negative Cotton effect at 335 nm (see Supplementary Data, Figure S59) establishing the 22*R* configuration and the *trans*-linkage of rings A/B (and hence α-orientation of CH_3_O-5) [35,36]. The structure of **20** was thus elucidated as physachenolide-A-5-methyl ether [(20*S*,22*R*)-18-acetoxy-6*β*,14*α*,17*β*,20*β*-tetrahydroxy-5*α*-methoxy-1-oxo -witha-2,24-dienolide].

Comparison of the spectroscopic data with those reported led to the identification of the remaining seventeen withanolides as physachenolide A (**21**) [38], physachenolide D (**3**) [38], 15*α*-hydroxyphysachenolide D (**22**) [18], 15*α*-acetoxyphysachenolide D (**4**) [27], orizabolide (**23**) [41], 15*α*-acetoxy-28-hydroxyphysachenolide D (**24**) [19], physacoztolide G (**25**) [27], 28-*O*-*β*-d-glucopyranosyl-physachenolide D (**26**) [27], physachenolide C (**8**) [38], 15*α*-acetoxyphysachenolide C (**27**) [18], 15*α*-hydroxyphysachenolide C (**28**) [29], 18-deacetylphysachenolide C (**29**) [29], physacoztolide H (**30**) [27], withanolide E (**5**) [42], withaperuvin L (**31**) [43], physacoztolide D (**32**) [28], and 18-acetoxy-17-*epi*-withanolide K (**33**) [19]. This constitutes the first report of the natural occurrence of 15*α*-hydroxyphysachenolide C (**28**) and 18-deacetylphysachenolide C (**29**).

A small number of chlorinated withanolides have previously been encountered in plants of Solanaceae as minor metabolites and many of these occur as 5,6-chlorohydrins containing 5*α*-chloro-6*β*-hydroxy substituents [25,44,45,46,47,48]. It has been suggested that the chlorine atom present in these 5,6-chlorohydrins may originate from NaCl present in the plant [2]. However, the occurrence of corresponding 5*β*,6*β*-epoxides as major matabolites in their source plants (as in *P. costomatl*) suggests that 5,6-chlorohydrins of withanolides may be possible artifacts formed from their corresponding 5*β*,6*β*-epoxides during the extraction of these plants and/or during the isolation of withanolides. The possibility of formation of withanolide chlorohydrins during the isolation process has previously been suggested [49] for which a probable mechanism involving acid catalyzed opening of the 5*β*,6*β*-epoxy moiety to generate 5,6-chlorohydrins has been proposed [25]. To test this, we exposed the major withanolide of *P. coztomatl*, physachenolide C (**8**), to 0.5% methanolic HCl at 25 °C for 30 min (TLC control). The investigation of the crude product mixture by HPLC suggested that under these mildly acidic conditions, the 5*β*,6*β*-epoxide ring of physachenolide C (**8**) underwent an acid-catalyzed ring opening to afford the corresponding 5,6-chlorohydrin [5*α*-chloro-6*β*-hydroxyphysachenolide C (**17**)], 5*α*-methoxy-6*β*-hydroxy analogue [physachenolide A-5-methyl ether (**19**)] and 5*α*,6*β*-dihydroxy analogue [physachenolide A (**21**)] (see Supplementary Data, Figure S62), all of which were encountered in *P. coztomatl*. Additional experiments to investigate whether these withanolides are genuine plant metabolites or artifacts are currently in progress.

### 2.2. Biological Activities of Withanolides from P. coztomatl

We have previously discovered that some 17*β*-hydroxywithanolides, including physachenolide C (**8**), were capable of selectively inhibiting the proliferation of prostate cancer cells at nanoMolar concentrations without affecting many other cancer cells and normal human fibroblast cells [19]. In this study, withanolides **3**–**5** and **8**–**33** obtained from aeroponically grown *P. crassifolia* were evaluated for their cytotoxic activity against a panel of four human prostate cancer (PC) cell lines, LNCaP and VCaP (androgen-sensitive PC), DU-145 and PC-3 (androgen-independent PC), human renal adenocarcinoma (ACHN) cell line, and normal human fibroblast cells, WI-38. Of those tested, withanolides **8**, **10**, **13**, **15**, **17**, and **18** showed >50% inhibition against at least one of the cancer cell lines at 5.0 μM concentration. Significantly, all those showing promising activity were 18-acetoxy-17*β*-hydroxywithanolides and these were then evaluated for their IC_50_s (concentrations required to inhibit 50% of the cells). The IC_50_ data obtained are depicted in Table 5.

**Figure 3:**
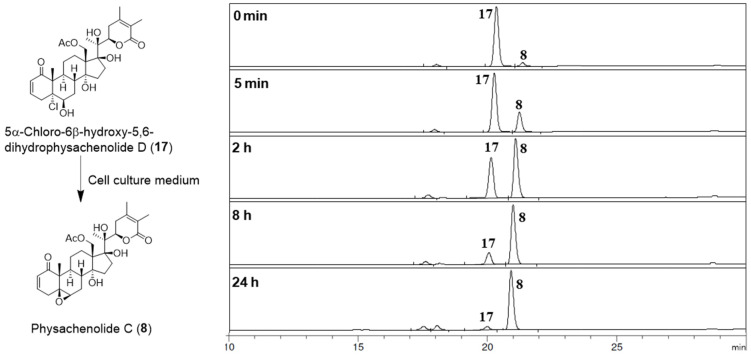
The HPLC analysis of 5α-chloro-6β-hydroxy-5,6-dihydrophysachenolide D (**17**) incubated with the cell culture medium (DMEM) showing facile and complete conversion of it to physachenolide C (**8**).

It is noteworthy that 5*α*-chloro-6*β*-hydroxy-5,6-dihdrophysachenolide D (**17**) containing a *trans*-fused A/B-ring system exhibited cytotoxic activities very close to those of physachenolide C (**8**) bearing a *cis*-fused A/B-ring system, against all the cell lines tested (Table 5). This is somewhat surprising as it contradicts our previous finding that the *cis*-fused A/B-ring conformation (as in **8**) is important for the cytotoxic activity of 17*β*-hydroxywithanolides [33]. This unexpected potent activity of 5*α*-chloro-6*β*-hydroxy-5,6-dihydrophysachenolide D (**17**) and other withanolide 5,6-chlorohydrins may be attributed to the possible conversion of these to their corresponding 5*β*,6*β*-epoxides in the cell culture medium. To test this, **17** was incubated with the cell culture medium (DMEM) used for the cytotoxicity assays with LNCaP and ACHN cell lines and under the conditions used for the assay (37 °C in a 5% CO_2_ incubator). The analysis of the incubation mixture by HPLC at intervals of 0 min, 5 min, 2 h, 8 h, and 24 h, suggested that its conversion to physachenolide C (**8**) is facile and almost complete in 24 h (Figure 3). Since the cytotoxicity assay involves incubation of the test compound for 72 h in the cell culture medium, it is very likely that the unexpected activity observed for 5*α*-chloro-6*β*-hydroxywithanolides is due to the conversion of these into their corresponding 5*β*,6*β*-epoxywithanolides.

## 3. Materials and Methods

### 3.1. General Methods and Materials

Optical rotations were measured at 25 °C with a JASCO Dip-370 digital polarimeter using MeOH as solvent. UV spectra were recorded in MeOH using a Shimadzu UV-1601 UV-Vis spectrometer. ECD spectra were measured with JASCO J-810 circular dichroism spectropolarimeter. 1D and 2D NMR spectra were recorded on a Bruker Avance III 400 NMR instrument at 400 MHz for ^1^H NMR and 100 MHz for ^13^C NMR. Chemical shift values (δ) are given in parts per million (ppm), and the coupling constants are in Hz. High-resolution MS were recorded on an Agilent G6224A TOF mass spectrometer. Normal phase column chromatography was performed using Baker silica gel 40 μm flash chromatography packing (J. T. Baker) and reversed-phase chromatography was carried out using BAKERBOND C_18_ 40 μm preparative LC packing (J. T. Baker). Analytical and preparative thin-layer chromatography (TLC) were performed on pre-coated 0.20 mm thickness plates of silica gel 60 F_254_ (Merck) and RP-18 F_254_ (Merck). HPLC purifications were carried out using 10 mm × 250 mm Phenomenex Luna 5 μm C-18 column (3 mL/min flow rate) with a Waters Delta Prep system consisting of a PDA 996 detector. MM2 energy minimizations of possible conformations of compounds were performed using Chem3D 15.0 from Perkin Elmer Inc. (Waltham, MA, USA).

The cell culture media used for the bioassays are: RPMI medium with 10% FBS, 1% glutamax, and 100 U/mL penicillin, and 100 μg/mL streptomycin for PC-3 cells; EMEM medium with 10% FBS, 1% glutamax, and 100 U/mL penicillin, and 100 μg/mL streptomycin for DU-145 and WI-38 cells; DMEM medium with 10% FBS and 100 U/mL penicillin, and 100 μg/mL streptomycin for VCaP cells; RPMI medium with 5% FCS, 2 mM L-glutamine, 1× nonessential amino acids, 1 mM sodium pyruvate, 100 U/mL penicillin, 100 μg/mL streptomycin, 10mM HEPES, and 5 × 10^−5^ M 2-mercaptoethanol for LNCaP and ACHN cells.

### 3.2. Aeroponic Cultivation and Harvesting of P. coztomatl

The seeds of *P. coztomatl* obtained from Trade Wind Fruit (P.O. Box 1102, Windsor, CA 95492, USA) were germinated in 1.0 inch Grodan rock-wool cubes in a Barnstead Lab-Line growth chamber kept at 28 °C under 16 h of fluorescent lighting and maintaining 25–50% humidity. After ca. 4 weeks in the growth chamber, seedlings with an aerial length of ca. 5.0 cm were transplanted to aeroponic culture boxes for further growth, as described previously for *Withania somnifera* and *Physalis crassifolia* [17,18]. Aerial parts of aeroponically grown plants were harvested when fruits were almost mature (ca. 3 months under aeroponic growth conditions). Harvested plant materials were dried in the shade, powdered, and stored at 5 °C prior to extraction.

### 3.3. Extraction, Isolation and Identification of Withanolides

Dried and powdered aerial parts of *P. coztomatl* (200.0 g) were extracted with MeOH (3.0 L) in an ultrasonic bath at 25 °C for 2 h, and then allowed to stand for overnight. After filtration, the resulting filtrate was concentrated under reduced pressure at 40 °C to afford the crude extract (45.0 g). The crude extract (45.0 g) was subjected to solvent–solvent partitioning between hexanes and 80% aqueous MeOH, and the resulting 80% aqueous MeOH layer was diluted with H_2_O to give 50% aqueous MeOH solution, which was further extracted with CHCl_3_ to afford the CHCl_3_ extract. These were concentrated to afford hexanes (3.2 g) and CHCl_3_ extracts (3.5 g). The 50% aq. MeOH layer obtained above was passed through a column of HP-20SS (Supelco, 200.0 g), washed with MeOH and concentrated yielding the 50% aq. MeOH fraction (0.28 g), which showed a TLC profile similar to the CHCl_3_ fraction. Thus, the combined CHCl_3_ and 50% aq. MeOH fractions (3.78 g) was subjected to column chromatography (CC) on RP C_18_ (200.0 g) and eluted with 600.0 mL each of 50%, 60%, 70%, 80%, 90% aq. MeOH and finally with MeOH to afford eleven fractions, A–K: A (155.9 mg) eluted with 50% aq. MeOH; B (163.9 mg) with 50% aq. MeOH; C (289.0 mg) with 60% aq. MeOH; D (160.0 mg) with 60% aq. MeOH; E (384.5mg) with 70% aq. MeOH; F (65.6 mg) with 70% aq. MeOH; G (105.3 mg) with 80% aq. MeOH; H (223.0 mg) with 80% aq. MeOH; I (208.3 mg) with 90% aq. MeOH; J (1007.3 mg) with 90% aq. MeOH; K (450.1 mg) with MeOH. Fraction C on further fractionation by silica gel (25.0 g) CC and eluting with 200.0 mL each of 95:5, 90:10, and 80:20 CHCl_3_/MeOH, and further purification of the resulting fractions by RP C_18_ HPLC or prep TLC afforded **11** (2.5 mg, *R*_f_ = 0.7, SiO_2_ TLC, 9:1 EtOAc/MeOH), **17** (65.4 mg, *R*_f_ = 0.3, SiO_2_ TLC, 95:5 CHCl_3_/MeOH), **18** (27.7 mg, *R*_f_ = 0.4, SiO_2_ TLC, 95:5 CHCl_3_/MeOH), and **26** (11.3 mg, *t*_R_ = 16.5 min, 55% aq. MeOH). Fraction D was fractionated by silica gel (25.0 g) CC and eluting with 200.0 mL each of 95:5, 90:10, and 80:20 CHCl_3_/MeOH. Further purification of the resulting fractions by RP C_18_ HPLC or prep TLC afforded **9** (6.0 mg, *R*_f_ = 0.4, SiO_2_ TLC, 8:2 EtOAc/MeOH), **11** (3.5 mg, *R*_f_ = 0.5, SiO_2_ TLC, 8:2 EtOAc/MeOH), **13** (7.0 mg, *t*_R_ = 22.0 min, 55% aq. MeOH), **15** (2.6 mg, *t*_R_ = 24.4 min, 55% aq. MeOH), **19** (3.4 mg, *t*_R_ = 16.3 min, 55% aq. MeOH), **21** (2.8 mg, *t*_R_ = 66.0 min, 47% aq. MeOH), **25** (18.9 mg, *t*_R_ = 72.3 min, 47% aq. MeOH), **29** (3.3 mg, *t*_R_ = 16.3 min, 55% aq. MeOH), and **32** (3.0 mg, *t*_R_ = 17.8 min, 55% aq. MeOH). Fraction E was fractionated by silica gel (25.0 g) CC and eluting with 200 mL each of 95:5, 90:10, and 80:20 CHCl_3_/MeOH, followed by further purification by RP C_18_ HPLC or preparative TLC to afford an additional amount of **9** (3.4 mg, *R*_f_ = 0.5, SiO_2_ TLC, 94:6 EtOAc/MeOH) together with **10** (1.7 mg, *R*_f_ = 0.3, SiO_2_ TLC, 9:1 CHCl_3_/MeOH), **12** (4.5 mg, *R*_f_ = 0.6, SiO_2_ TLC, 9:1 CHCl_3_/MeOH), **16** (1.0 mg, *R*_f_ = 0.6, RP C_18_ TLC, 65% aq. MeOH), **20** (1.8 mg, *t*_R_ = 41.2 min, 55% aq. MeOH), **21** (1.4 mg, *t*_R_ = 21.0 min, 55% aq. MeOH), **23** (33.6 mg, *R*_f_ = 0.6, SiO_2_ TLC, 9:1 CHCl_3_/MeOH), **24** (4.1 mg, *t*_R_ = 25.4 min, 55% aq. MeOH), **25** (4.1 mg, *t*_R_ = 25.4 min, 55% aq. MeOH), **8** (93.8 mg, *t*_R_ = 12.4 min, 65% aq. MeOH), **27** (35.7 mg, *t*_R_ = 20.5 min, 60% aq. MeOH), **28** (2.1 mg, *R*_f_ = 0.3, SiO_2_ TLC, 95:5 CHCl_3_/MeOH), **30** (4.3 mg, *t*_R_ = 31.1 min, 55% aq. MeOH), and **31** (2.6 mg, *R*_f_ = 0.3, SiO_2_ TLC, 94:6 EtOAc/MeOH). Fraction F was further fractionated by silica gel (20.0 g) CC and eluting with 200 mL each of 95:5 and 90:10 CHCl_3_/MeOH. Further purification of the fractions thus obtained by RP C_18_ HPLC yielded **14** (2.5 mg, *t*_R_ = 20.1 min, 65% aq. MeOH), **22** (1.1 mg, *t*_R_ = 22.9 min, 60% aq. MeOH), **4** (1.7 mg, *t*_R_ = 17.8 min, 62% aq. MeOH), and **8** (3.1 mg, *t*_R_ = 12.4 min, 65% aq. MeOH). Fraction G was further fractionated by silica gel (25.0 g) CC and eluting with 250 mL each of 98:2 and 96:4 CHCl_3_/MeOH. Purification of the resulting fractions by RP C_18_ HPLC afforded **3** (47.0 mg, *t*_R_ = 28.0 min, 62% aq. MeOH), **4** (7.0 mg, *t*_R_ = 28.0 min, 62% aq. MeOH) and **5** (2.0 mg, *t*_R_ = 24.2 min, 62% aq. MeOH). Fraction H was fractionated by silica gel (25.0 g) CC and eluting with 250 mL each of 98:2, 96:4, 94:6, and 92:8 CHCl_3_/MeOH. The resulting sub-fractions were further purified by RP C_18_ HPLC to afford **3** (1.5 mg, *R*_f_ = 0.4, SiO_2_ TLC, 9:1 CHCl_3_/MeOH) and **33** (0.9 mg, *t*_R_ = 16.7 min, 70% aq. MeOH).

Physacoztolide I (**9**): amorphous, colorless solid; [*α*]${}_{D}^{25}$ + 32.6 (*c* 0.34, MeOH); UV (MeOH) *λ*_max_ (log *ε*) 224 (4.09) nm; ECD (MeOH) *λ*_max_ (Δ*ε*) 338 (−2.36), 256 (4.25); ^1^H and ^13^C NMR data, see Table 2; positive HRESIMS *m/z* 711.2986 [M + Na]^+^ (calcd. for C_36_H_48_O_13_Na, 711.2993).

Physacoztolide J (**10**): amorphous, colorless solid; [*α*]${}_{D}^{25}$ + 46.4 (*c* 0.25, MeOH); UV (MeOH) *λ*_max_ (log *ε*) 224 (4.21) nm; ECD (MeOH) *λ*_max_ (Δ*ε*) 338 (−2.92), 257 (4.74); ^1^H and ^13^C NMR data, see Table 2; positive HRESIMS *m/z* 695.3037 [M + Na]^+^ (calcd. for C_36_H_48_O_12_Na, 695.3038).

Physacoztolide K (**11**): amorphous, colorless solid; [*α*]${}_{D}^{25}$ + 46.4 (*c* 0.25, MeOH); UV (MeOH) *λ*_max_ (log *ε*) 224 (4.21) nm; ECD (MeOH) *λ*_max_ (Δ*ε*) 338 (−2.92), 256 (2.38); ^1^H and ^13^C NMR data, see Table 2; positive HRESIMS *m/z* 567.2539 [M + Na]^+^ (calcd. for C_30_H_40_O_9_Na, 567.2570).

Physacoztolide L (**12**): amorphous, colorless solid; [*α*]${}_{D}^{25}$ − 4.0 (*c* 0.10, MeOH); UV (MeOH) *λ*_max_ (log *ε*) 223 (3.77) nm; ECD (MeOH) *λ*_max_ (Δ*ε*) 338 (−2.37), 256 (1.37); ^1^H and ^13^C NMR data, see Table 2; positive HRESIMS *m/z* 569.2727 [M + Na]^+^ (calcd. for C_30_H_42_O_9_Na, 569.2727).

28-Hydroxyphysachenolide C (**13**): amorphous, colorless solid; [*α*]${}_{D}^{25}$ + 97.8 (*c* 0.30, MeOH); UV (MeOH) *λ*_max_ (log *ε*) 224 (4.09) nm; ECD (MeOH) *λ*_max_ (Δ*ε*) 342 (1.29), 258 (2.59); ^1^H and ^13^C NMR data, see Table 3; positive HRESIMS *m/z* 583.2520 [M + Na]^+^ (calcd. for C_30_H_40_O_10_Na, 583.2519).

15α-Acetoxy-28-hydroxyphysachenolide C (**14**)**:** amorphous, colorless solid; [*α*]${}_{D}^{25}$ + 116.1 (*c* 0.27, MeOH); UV (MeOH) *λ*_max_ (log *ε*) 224 (4.16) nm; ECD (MeOH) *λ*_max_ (Δ*ε*) 341 (1.25), 258 (2.86); ^1^H and ^13^C NMR data, see Table 3; positive HRESIMS *m/z* 641.2579 [M + Na]^+^ (calcd. for C_32_H_42_O_12_Na, 641.2574).

28-Oxophysachenolide C (**15**): amorphous, colorless solid; [*α*]${}_{D}^{25}$ + 107.2 (*c* 0.34, MeOH); UV (MeOH) *λ*_max_ (log *ε*) 225 (4.13) nm; ECD (MeOH) *λ*_max_ (Δ*ε*) 341 (1.37), 258 (3.06); ^1^H and ^13^C NMR data, see Table 3; positive HRESIMS *m/z* 581.2355 [M + Na]^+^ (calcd. for C_30_H_38_O_10_Na, 581.2363).

Physacoztolide M (**16**): amorphous, colorless solid; [*α*]${}_{D}^{25}$ + 46.4 (*c* 0.25, MeOH); UV (MeOH) *λ*_max_ (log *ε*) 225 (4.11) nm; ECD (MeOH) *λ*_max_ (Δ*ε*) 340 (−2.71), 256 (5.01); ^1^H and ^13^C NMR data, see Table 3; positive HRESIMS *m/z* 653.2937 [M + Na]^+^ (calcd. for C_34_H_46_O_11_Na, 653.2938).

5*α*-Chloro-6*β*-hydroxy-5,6-dihydrophysachenolide D (**17**): amorphous, colorless solid; [*α*]${}_{D}^{25}$ + 58.5 (*c* 0.34, MeOH); UV (MeOH) *λ*_max_ (log *ε*) 227 (4.10) nm; ECD (MeOH) *λ*_max_ (Δ*ε*) 336 (−1.24), 252 (3.82); ^1^H and ^13^C NMR data, see Table 4; positive HRESIMS *m/z* 603.2349 [M + Na]^+^ (calcd. for C_30_H_41_ClO_9_Na, 603.2337).

15α-Acetoxy-5*α*-chloro-6*β*-hydroxy-5,6-dihydrophysachenolide D (**18**): amorphous, colorless solid; [*α*]${}_{D}^{25}$ + 74.4 (*c* 0.17, MeOH); UV (MeOH) *λ*_max_ (log *ε*) 227 (4.05) nm; ECD (MeOH) *λ*_max_ (Δ*ε*) 335 (−1.18), 252 (3.41); ^1^H and ^13^C NMR data, see Table 4; positive HRESIMS *m/z* 661.2437 [M + Na]^+^ (calcd. for C_32_H_43_ClO_11_Na, 661.2392).

28-Hydroxy-5*α*-chloro-6*β*-hydroxy-5,6-dihydrophysachenolide D (**19**): amorphous, colorless solid; [*α*]${}_{D}^{25}$ + 52.7 (*c* 0.10, MeOH); UV (MeOH) *λ*_max_ (log *ε*) 227 (4.05) nm; ECD (MeOH) *λ*_max_ (Δ*ε*) 335 (−1.33), 255 (2.82); ^1^H and ^13^C NMR data, see Table 4; positive HRESIMS *m/z* 619.2292 [M +Na]^+^ (calcd. for C_30_H_41_ClO_10_Na, 619.2286).

Physachenolide A-5-methyl ether (**20**): amorphous, colorless solid; [*α*]${}_{D}^{25}$ + 59.8 (*c* 0.45, MeOH); UV (MeOH) *λ*_max_ (log *ε*) 225 (4.00) nm; ECD (MeOH) *λ*_max_ (Δ*ε*) 335 (−0.60), 257 (1.70); ^1^H and ^13^C NMR data, see Table 4; positive HRESIMS *m/z* 599.2836 [M + Na]^+^ (calcd. for C_31_H_44_O_10_Na, 599.2832).

### 3.4. Acid Hydrolysis of Glycosides ***9***, ***10***, and ***16***

To a solution of each glycoside (**9**, **10** or **16**, 0.5 mg) in MeOH (0.5 mL) was added 2N HCl solution (0.5 mL). The mixture was heated at 100 °C. After 1 h (TLC control), the reaction mixtures were concentrated and the residues thus obtained were chromatographed over a column of silica gel (0.5 g) using CHCl_3_/MeOH (8:2) as the eluent. Fractions containing the sugar were collected based on their TLC profiles, concentrated, dissolved in water for qualitative measurement of [*α*]_D_.

### 3.5. Cytotoxicity Assay

A tetrazolium dye-based colorometric (MTT) assay was used for evaluating cytotoxicity of the compounds against cancer cell lines, LNCaP (androgen-sensitive prostate adenocarcinoma), PC-3 (androgen-insensitive prostate adenocarcinoma), DU-145 (androgen-insensitive prostate adenocarcinoma), VCaP (androgen-sensitive metastatic prostate cancer), and ACHN (renal carcinoma), and normal human lung fibroblast cells, WI-38. The cells were plated at 1000–4000 cells/well (depending on the cell growth rate) in 96-well flat-bottomed microplates. After incubation at 37 °C for 24 h in an atmosphere of 5% CO_2_, serial dilutions of compounds in DMSO were added to triplicate wells so that the final DMSO concentration in each well is <0.2%. Doxorubicin and DMSO were used as positive and negative controls, respectively. After incubation for 72 h at 37 °C in an atmosphere of 5% CO_2_, MTT solution (2 mg/mL, 25.0 μL) was added to each well, and continued to incubate for 3–4 h at 37 °C. The media were removed and 100 μL/well of DMSO was added before data acquisition using a microplate reader at 570 nm.

### 3.6. Conversion of 5α-Chloro-6β-hydroxy-5,6-dihydrophysachenolide D (***17***) to Physachenolide C (***8***)

A solution 5*α*-chloro-6*β*-hydroxy-5,6-dihydrophysachenolide D (**17**) (0.2 mg) in DMSO (1.0 μL) was added to the RPMI medium (1.0 mL) used for the cytotoxicity assays with LNCaP and ACHN cells (see General Methods and Materials). The solution was kept at 37 °C in a 5% CO_2_ incubator, and 100.0 μL samples were withdrawn for HPLC analysis at 0 min, 5 min, 2 h, 8 h, and 24 h. The HPLC analysis was carried out on an Agilent HP 1100 HPLC system with a Phenomenex Spherisorb 5 μ ODS (2) 80A, 250 mm × 4.6 mm HPLC column (flow rate: 0.7 mL/min; MeOH-H_2_O gradient solvent system by increasing MeOH from 40% to 100% in 30 min; UV detection at 230 nm). The product formed was identified as physachenolide C (**8**) by its retention time and the peak enhancement method.

## 4. Conclusions

Withanolides constitute one of the most interesting classes of natural products due to their diversity of structures and biological activities. The work reported here further supports our previous findings that the application of the aeroponic technique for cultivation of plants of Solanaceae is a convenient, reproducible, and superior method for production and structural diversification of withanolides. Investigation of aeroponically grown *Physalis coztomatl* afforded 29 withanolides including 12 new withanolides (**9**–**20**), and 17 known withanolides (**3**–**5**, **8**, and **21**–**33**). Evaluation of these withanolides against a panel of prostate cancer (LNCaP, VCaP, DU-145, and PC-3) and renal carcinoma (ACHN) cell lines, and normal human foreskin fibroblast (WI-38) cells suggested that **8**, **13**, **15**, and **17**–**19** had potent and selective activity for prostate cancer cell lines. This work also resulted in the discovery that the potent cytotoxic activity of withanolide 5,6-chlorohydrins may be due to their facile conversion into the corresponding 5*β*,6*β*-epoxides in the cell culture medium used for the bioassay.

## Figures and Tables

**Table 1:** Comparison of the number of withanolides and % yields of major withanolides isolated from aeroponically grown and wild-crafted plants of some plants of Solanaceae.

Plant	Cultivation Method/Source	Number of Withanolides Isolated ^a^	Major Withanolides(% Yield) ^b^	Refs and Notes
*Withania somnifera*	Aeroponics	14	Withaferin A (**1**) (0.42)	[15,17,23]
			2,3-Dihydrowithaferin A-3β-O-sulfate (**2**) (0.51)	
	Wild-crafted (chemotype I)	7	Withaferin A (**1**) (0.23)	[24]
*Physalis crassifolia*	Aeroponics	18	Physachenolide D (**3**) (0.30)	[18]
			15α-Acetoxyphysachenolide D (**4**) (0.03)	
	Wild-crafted	5	Physachenolide D (**3**) (0.01)	[19]
			15α-Acetoxyphysachenolide D (**4**) (0.005)	
*Physalis peruviana*	Aeroponics	25	Withanolide E (**5**) (0.18)	[20]
			4β-Hydroxywithanolide E (**6**) (0.15)	
	Wild-crafted	17	Withanolide E (**5**) ^c^	[25]
			4β-Hydroxywithanolide E (**6**) (0.008)	
*Physalis philadelphica*	Aeroponics	11	Ixocarpalactone B (**7**) (0.10)	[21]
	Wild-crafted	7	Ixocarpalactone B (**7**) (0.02)	[26]
*Physalis coztomatl*	Aeroponics	29	Physachenolide C (**8**) (0.05)	This study
			Physachenolide D (**3**) (0.02)	
	Wild-crafted	8	Physachenolide C (**8**) ^d^	[27]
			Physachenolide D (**3**) (0.02)	

^a^ The number of withanolides for wild-crafted/soil-grown plant refers to the referenced study reporting highest yield(s) of the major withanolide(s). ^b^ Since **2** is a prodrug of **1** [15], total % yield of withaferin A (**1**) in aeroponically-grown plant is 0.93. ^c^ Yield not reported. ^d^ Not encountered in wild-crafted plants [27,28].

**Table 2:** ^1^H and ^13^C NMR data for **9**–**12** in CDCl_3_.

Position	9 ^a^		10 ^a^		11		12	
	*δ*_H_	*δ*_C_	*δ*_H_	*δ*_C_	*δ*_H_	*δ*_C_	*δ*_H_	*δ*_C_
1		204.4		204.7		204.6		203.9
2	5.83 (br d, 9.6)	127.8	5.84 (br d, 10.0)	127.8	5.84 (dd, 10.0, 2.0)	127.7	5.85 (dd, 10.0, 2.0)	127.9
3	6.77 (br d, 9.6)	145.7	6.77 (ddd, 10.0, 4.8, 2.8)	145.4	6.78 (ddd, 10.0, 4.8, 2.4)	145.9	6.76 (ddd, 10.0, 4.8, 2.4)	145.3
4	3.28 (m)	33.5	3.28 (m)	33.5	3.27 (br d, 21.2)	33.5	3.27 (br d, 21.6)	33.4
	2.83 (m)		2.83 (dd, 21.6, 4.8)		2.83 (dd, 21.2, 2.4)		2.82 (dd, 21.6, 4.8)	
5		135.2		136.1		135.0		135.3
6	5.57 (br s)	124.9	5.57 (d, 4.8)	124.4	5.57 (br s)	124.7	5.57 (d, 5.6)	124.4
7	2.22 (m)	25.5	2.00 (m)	30.5	2.08 (m)	25.3	2.10 (m)	25.4
	1.82 (m)		1.62 (m)		1.81 (m)		1.81 (m)	
8	1.85 (m)	35.1	1.69 (m)	31.9	1.82 (m)	36.2	1.80 (m)	36.4
9	2.37 (m)	36.0	1.76 (m)	43.4	2.07 (m)	36.1	2.15 (m)	36.1
10		50.8		50.6		50.6		50.8
11	2.31 (m)	22.0	2.34 (m)	23.3	2.15 (m)	22.3	2.21 (m)	22.0
	1.51 (m)		1.52 (m)		1.34 (m)		1.34 (m)	
12	2.34 (m)	24.8	2.64 (m)	25.3	2.09 (m)	27.2	1.96 (m)	27.1
	1.95 (m)		2.55 (m)		1.81 (m)		1.91 (m)	
13		56.3		50.6		50.5		50.6
14		83.4	1.76 (m)	57.6		83.3		82.7
15	2.43 (m)	41.2	2.09 (m)	31.9	1.59 (dd, 12.2, 9.2)	32.1	1.61 (m)	32.4
	2.17 (m)				1.40 (m)		1.39 (m)	
16	5.82 (br s)	126.7	5.74 (br s)	128.3	2.00 (m)	21.2	1.86, (m)	20.7
					1.88, (m)			
17		151.1		153.0	2.70 (t, 9.2)	49.3	2.73 (t, 9.7)	49.9
18	4.59 (d, 10.4)	67.3	4.56 (d, 11.2)	66.6	4.40 (d, 11.6)	62.9	4.89, (d, 11.6)	62.9
	3.99 (d, 10.4)		4.02 (d, 11.2)		3.98 (d, 11.6)		3.70, (d, 11.6)	
19	1.22 (s)	18.7	1.23 (s)	18.9	1.20 (s)	18.9	1.22 (s)	18.8
20		74.4		74.7		76.1		76.5
21	1.28 (s)	24.8	1.30 (s)	26.6	1.43 (s)	23.4	1.40 (s)	20.9
22	4.49 (m)	80.8	4.45 (m)	81.5	4.23 (d, 7.8)	85.8	3.60 (d, 8.4)	75.9
23	2.50–2.78 (m)	25.1	2.38 (m)	32.3	4.35 (d, 7.8)	66.9	4.06 (dd, 8.4, 8.0)	81.1
			1.78 (m)					
24		148.2		147.6		156.7	2.32 (m)	51.0
25		123.4		123.5		124.5	2.29 (m)	37.0
26		165.9		165.8		164.6		177.6
27	1.85 (s)	12.3	1.86 (s)	12.3	4.34 (s)	57.3	1.28 (d, 6.8)	14.1
28	4.46 (m)	67.7	4.45 (m)	68.0	2.06 (s)	15.4	3.83 (dd, 11.2, 2.0)	63.3
							3.64, (dd, 11.2, 7.6)	
OAc-18	2.07 (s)	21.3	2.08 (s)	21.3	2.07 (s)	21.2	2.14 (s)	21.2
		171.4		171.8		170.7		169.7
Glc-1′	4.27 (d, 6.0)	102.5	4.30 (d, 7.2)	102.5				
Glc-2′	3.38 (m)	73.3	3.39 (m)	73.4				
Glc-3′	3.26 (m)	75.8	3.28 (m)	75.8				
Glc-4′	3.55 (m)	69.4	3.60 (m)	69.8				
Glc-5′	3.47 (m)	76.4	3.51 (m)	76.4				
Glc-6′	3.81 (m)	61.2	3.82 (brs)	61.5				

^a^ CDCl_3_/CD_3_OD (100:1) was used as the solvent.

**Table 3:** ^1^H and ^13^C NMR data for **13**–**16**.

Position	13 ^a^		14 ^a^		15 ^b^		16 ^c^	
	*δ*_H_	*δ*_C_	*δ*_H_	*δ*_C_	*δ*_H_	*δ*_C_	*δ*_H_	*δ*_C_
1		205.4		205.4		203.1		205.0
2	5.99 (dd, 10.0, 2.8)	130.0	5.98 (dd, 10.0, 2.8)	129.9	5.98 (dd, 10.0, 2.8)	129.6	5.79 (dd, 10.0, 2.0)	127.7
3	6.97 (ddd, 10.0, 6.0, 2.4)	147.1	6.97 (ddd, 10.0, 6.0, 2.4)	147.1	6.80 (ddd,10.0, 6.4, 2.0)	144.1	6.74 (ddd, 10.0, 4.8, 2.4)	145.9
4	2.96 (dt, 18.8, 2.4)	33.8	2.96 (dt, 18.8, 2.4)	33.8	2.93 (dt, 18.4, 2.4)	32.8	3.23 (m)	33.4
	1.94 (m)		1.94 (m)		1.85 (m)		2.79 (dd, 21.6, 4.8)	
5		63.4		63.2		62.0		134.8
6	3.23 (br s)	65.0	3.20 (d, 2.0)	65.0	3.15 (br s)	63.9	5.57 (d, 5.6)	125.3
7	1.95 (m)	27.7	2.05 (m)	27.4	1.94 (m)	26.3	2.20 (m)	25.3
	1.87 (m)		1.93 (m)				1.79 (m)	
8	1.92 (m)	35.5	2.65 (m)	35.9	1.87 (m)	34.2	1.87 (m)	34.4
9	1.83 (m)	38.7	1.89 (m)	38.6	1.89 (m)	36.8	2.28 (m)	35.8
10		49.7		49.8		48.4		50.6
11	2.03 (m)	24.5	2.05 (m)	24.5	2.06 (m)	22.8	2.19 (m)	22.2
	1.38 (m)		1.40 (m)		1.34 (m)		1.55 (m)	
12	2.15 (m)	26.6	2.24 (m)	26.6	2.18 (m)	25.5	2.20 (m)	28.3
	1.89 (m)		1.85 (m)		1.68 (m)		1.47 (m)	
13		58.6		58.6		57.5		52.2
14		83.0		81.3		81.5		84.5
15	1.62 (m)	33.8	5.06 (dd, 9.2, 8.4)	77.5	1.66 (m)	32.9	2.29 (m)	39.7
	1.55 (m)				1.59 (m)		2.14 (m)	
16	2.56 (m)	37.8	2.36 (m)	43.8	2.64 (m)	37.8	5.78 (br s)	124.5
	1.65 (m)		2.24 (m)		1.58 (m)			
17		88.9		85.8		87.9		155.9
18	4.36 (d, 11.2)	65.9	4.46 (d, 11.2)	65.4	4.40 (d, 11.6)	64.7	1.11 (s)	22.3
	4.30 (d, 11.2)		4.32 (d, 11.2)		4.28 (d, 11.6)			
19	1.19 (s)	15.3	1.20 (s)	15.4	1.20 (s)	14.8	1.18 (s)	18.6
20		79.8		80.0		79.2		74.6
21	1.39 (s)	19.0	1.35 (s)	19.1	1.43 (s)	19.0	1.26 (s)	22.4
22	4.86 (dd, 13.6, 3.2)	83.7	4.86 (dd, 13.6, 3.2)	83.6	4.90 (dd, 13.6, 3.2)	81.3	4.37 (t, 8.0)	81.1
23	3.22 (dd, 18.8, 2.8)	30.3	3.18 (dd, 20.4, 2.0)	30.3	3.14 (m)	24.5	2.63 (m)	25.0
	2.38 (m)		2.37 (m)		2.30, (m)			
24		154.9		154.9		142.3		149.0
25		122.4		122.4		138.1		122.8
26		169.1		169.0		165.6		166.2
27	1.88 (s)	12.1	1.87 (s)	12.1	2.35 (br s)	11.5	1.82 (s)	11.9
28	4.36 (d, 13.6)	61.9	4.37 (d, 14.0)	61.8	10.30 (s)	190.2	4.42 (br s)	67.7
	4.24 (d, 13.6)		4.20 (d, 14.0)					
OAc-18	2.13 (s)	21.3	2.13 (s)	21.3	2.15 (s)	21.1		
		173.5		173.1		170.8		
OAc-15			2.06 (s)	21.4				
				172.6				
Glc-1′							4.23 (d, 8.0)	102.5
Glc-2′							3.26 (m)	73.3
Glc-3′							3.23 (m)	75.9
Glc-4′							3.39 (m)	69.8
Glc-5′							3.37 (m)	76.4
Glc-6′							3.79 (dd, 12.0, 2.8)	61.5
							3.71 (dd, 12.0, 4.4)	

^a^ CD_3_OD was used as the solvent. ^b^ CDCl_3_ was used as the solvent. ^c^ CDCl_3_/CD_3_OD (100:1) was used as the solvent.

**Table 4:** ^1^H and ^13^C NMR data for **17**–**20** in CDCl_3_.

Position	17		18		19 ^a^		20 ^b^		21 ^b,c^
	*δ*_H_	*δ*_C_	*δ*_H_	*δ*_C_	*δ*_H_	*δ*_C_	*δ*_H_	*δ*_C_	*δ*_C_
1		201.3		201.0		204.1		204.8	205.3
2	5.89 (dd, 10.0, 2.4)	128.6	5.89 (dd, 10.0, 2.4)	128.5	5.83 (dd, 10.0, 2.0)	129.2	5.73 (dd, 10.0, 2.8)	129.0	127.8
3	6.62 (br dd, 10.0, 3.6)	141.2	6.63 (ddd, 10.0, 4.8, 2.4)	141.5	6.75 (ddd, 10.0, 4.8, 2.0)	144.1	6.48 (ddd, 10.0, 5.2, 2.0)	139.6	142.2
4	3.49 (br d, 20.0)	37.2	3.53 (dt, 20.4, 2.4)	37.2	3.56 (br d, 20.0)	38.7	2.95 (dt, 18.8, 2.4)	27.4	35.0
	2.49 (m)		2.47 (dd, 20.4, 8.0)		2.49 (dd, 20.0, 4.8)		2.28 (m)		
5		79.5		79.0		82.3		81.3	77.3
6	4.06 (br s)	75.0	3.97 (br s)	74.6	3.93 (t, 2.9)	75.6	3.86 (brs)	68.4	73.6
7	2.54 (m)	29.7	2.61 (m)	28.8	2.38 (m)	30.4	1.98 (m)	29.4	28.5
	1.52 (m)		1.54 (m)		1.54 (m)		1.37 (m)		
8	2.13 (m)	34.6	2.82 (dt, 4.0, 12.0)	34.8	2.26 (m)	35.9	2.02 (m)	33.9	33.4
9	2.76 (m)	34.9	2.37 (dt, 4.0, 12.0)	34.8	2.67 (m)	36.4	2.61 (m)	33.6	33.9
10		53.0		52.5		54.5		52.7	51.8
11	2.50 (m)	22.4	2.54 (m)	22.5	2.39 (m)	24.0	2.35 (m)	22.6	22.6
	1.22 (m)		1.24 (m)		1.24 (m)		1.18 (m)		
12	2.40 (m)	26.2	2.46 (m)	26.1	2.28 (m)	27.2	2.30 (m)	26.1	26.1
	1.81 (m)		1.86 (m)		1.94 (m)		1.75 (m)		
13		57.8		58.0		58.9		57.5	57.4
14		81.9		80.0		83.8		82.0	82.6
15	1.68 (m)	32.9	5.20 (t, 8.8)	75.8	1.58–1.71 (m)	33.6	1.63 (m)	32.6	32.4
	1.59 (m)						1.51 (m)		
16	2.71(m)	37.9	2.53 (m)	42.5	2.60 m	37.8	2.61 (m)	37.4	37.0
	1.55 m		2.26 (m)		1.68 m		1.47 (m)		
17		88.2		84.7		89.1		87.9	87.7
18	4.43 (s)	65.5	4.79 (d, 11.6)	64.9	4.40 (s)	66.4	4.37 (d, 11.2)	65.5	65.3
			4.22 (d, 11.6)				4.30 (d, 11.2)		
19	1.36 (s)	16.1	1.36 (s)	16.5	1.38 (s)	17.0	1.22 s	15.4	15.5
20		78.9		79.4		79.9		78.2	78.1
21	1.41 (s)	19.3	1.38 (s)	19.2	1.40 (s)	19.2	1.31 s	18.4	18.1
22	4.90 (t, 8.4)	79.7	4.91 (br d, 8.0)	79.7	4.90 (m)	84.0	4.84 (dd, 13.6, 3.2)	80.8	81.0
23	2.53 (m)	33.8	2.50 (m)	33.8	2.40 (m)	30.3	2.58 (m)	33.7	33.6
							2.44 (m)		
24		149.7		149.9		154.9		150.6	151.0
25		121.8		121.8		122.5		121.4	121.2
26		165.7		165.7		169.2		167.2	167.5
27	1.88 (s)	12.4	1.88 (s)	12.4	1.89 (s)	12.1	1.82 s	12.2	12.0
28	1.92 (s)	20.7	1.92 (s)	20.7	4.39 (d, 14.0)	62.0	1.88 s	20.6	20.5
					4.23 (d, 14.0)				
OAc-18	2.07 (s)	21.3	2.09 (s)	21.4	2.12 (s)	21.3	2.03 s	21.3	21.2
		170.4		171.4		173.6		171.1	171.2
OAc-15			2.08 (s)	21.8					
				172.2					
OMe							2.93 s	49.6	

^a^ CD_3_OD was used as the solvent. ^b^ CDCl_3_/CD_3_OD (100:1) was used as the solvent. ^c 13^C NMR data obtained in CDCl_3_/CD_3_OD (100:1) for physachenolide A (**21**) are included for the purpose of comparison as the reported ^13^C NMR data for **21** were for CDCl_3_/DMSO-d_6_ [38].

**Table 5:** Cytotoxicity data of withanolides from *Physalis coztomatl* against a panel of selected tumor cell lines and normal cells ^a^.

Compound	Cell Line ^b^										
	LNCaP		DU-145		PC-3		VCaP		ACHN		WI-38
	Activity	SI ^c^	Activity	SI ^c^	Activity	SI ^c^	Activity	SI ^c^	Activity	SI ^c^	
**8**	0.03 ± 0.01	15.0	0.26 ± 0.01	1.7	0.06 ± 0.01	7.5	0.03 ± 0.01	15.0	1.02 ± 0.20	0.4	0.45 ± 0.11
**13**	2.78 ± 0.66	>1.8	>5.0		2.90 ± 0.32	>1.7	1.11 ± 0.19	>4.5	>5.0		>5.0
**15**	1.04 ± 0.18	3.6	2.67 ± 0.15	1.4	1.18 ± 0.21	3.2	0.82 ± 0.11	4.6	3.98 ± 0.10	0.9	3.77 ± 0.06
**17**	0.03 ± 0.01	17.0	0.67 ± 0.08	0.8	0.09 ± 0.01	5.7	0.08 ± 0.01	6.4	1.73 ± 0.18	0.3	0.51 ± 0.03
**18**	0.64 ± 0.16	>7.8	4.53 ± 0.55	>1.1	0.86 ± 0.19	>5.8	0.27 ± 0.08	>18.5	>5.0		>5.0
**19**	1.98 ± 0.44	>2.5	>5.0		2.67 ± 0.21	>1.9	1.26 ± 0.22	4.0	>5.0		>5.0
Doxorubicin	0.11 ± 0.02		0.04 ± 0.01		0.34 ± 0.05		0.67 ± 0.06		0.05 ± 0.01		0.80 ± 0.08

^a^ Results are expressed as IC_50_ values in μM. Doxorubicin and DMSO were used as positive and negative controls. ^b^ Key: LNCaP = androgen-sensitive human prostate adenocarcinoma; DU-145 = androgen-independent human prostate cancer; PC-3 = androgen-independent human prostate cancer; VCaP = androgen-sensitive human prostate cancer; ACHN = human renal adenocarcinoma; WI-38 = normal human fibroblast cells; ^c^ SI = Selectivity Index (against normal cells, WI-38).

## Data Availability

The data presented in this study are available on request from the corresponding author.

## Supplementary Materials

The following supporting information can be downloaded online. Figure S1: Aeroponic cultivation of plants of Solanaceae; Figure S2: Aeroponic cultivation of *Withania somnifera*; Figure S3: Aeroponic cultivation of *Physalis crassifolia*; Figures S4–S58: 1D and 2D NMR spectra of withanolides **9**–**20**; Figure S59: CD spectra of compounds **9**–**20**; Figure S60: Key HMBC correlations of and key ^1^H–^1^H correlations of **11**, **12**, **19**, and **20**; Figure S61: Key NOESY correlations of **11**, **12**, **17**, and **18**; Figure S62: Investigation of products formed on exposure of physachenolide C (**8**) to mild acidic conditions by HPLC.

## Sample Availability

Samples of the major compounds isolated from *P. coztomatl* are available from the corresponding author.
