# Supplementary material for: Production and Structural Diversification of Withanolides by Aeroponic Cultivation of Plants of Solanaceae: Cytotoxic and Other Withanolides from Aeroponically Grown Physalis coztomatl
Source: Molecules. 2022 Jan 28;27(3):909. doi: 10.3390/molecules27030909 (PMC8838488; doi:10.3390/molecules27030909)

Supplementary Materials for

Production and Structural Diversification of Withanolides by Aeroponic Cultivation of Plants of Solanaceae: Cytotoxic and other Withanolides from Aeroponically Grown *Physalis coztomatl*

Ya-Ming Xu,[1] E. M. Kithsiri Wijeratne,[1] Manping X. Liu,[1] Li-Jiang Xuan,[2] Wen-Qiong Wang,[2] and A. A. Leslie Gunatilaka[1,*]

[1] Southwest Center for Natural Products Research, School of Natural Resources and the Environment, College of Agriculture and Life Sciences, University of Arizona, 250 E. Valencia Road, Tucson, Arizona 85706, United States; yamingx@arizona.edu; Kithsiri@cals.arizona.edu; manpingliu033@gmail.com; leslieg1@email.arizona.edu

[2] State Key Laboratory of Drug Research, Shanghai Institute of Materia Medica, Chinese Academy of Sciences, 501 Haike Road, Zhangjiang Hi-Tech Park, Shanghai 201203, P. R. China; ljxuan@simm.ac.cn; wenqiong1019@126.com

Table of Contents

**Figure S1.**	Aeroponic cultivation of plants of Solanaceae	**S4**
**Figure S2.**	Aeroponic cultivation of *Withania somnifera*	**S5**
**Figure S3.**	Aeroponic cultivation of *Physalis crassifolia*	**S6**
**Figure S4.**	^{1}H NMR spectrum (400 MHz) of physacoztolide I (**9**) in $CDCl_3/CD_3OD$ (100:1)	**S7**
**Figure S5.**	^{13}C NMR spectrum (100 MHz) of physacoztolide I (**9**) in $CDCl_3/CD_3OD$ (100:1)	**S7**
**Figure S6.**	HSQC spectrum (400 MHz) of physacoztolide I (**9**) in $CDCl_3/CD_3OD$ (100:1)	**S8**
**Figure S7.**	HMBC spectrum (400 MHz) of physacoztolide I (**9**) in $CDCl_3/CD_3OD$ (100:1)	**S8**
**Figure S8.**	^{1}H NMR spectrum (400 MHz) of physacoztolide J (**10**) in $CDCl_3/CD_3OD$ (100:1)	**S9**
**Figure S9.**	^{13}C NMR spectrum (100 MHz) of physacoztolide J (**10**) in $CDCl_3/CD_3OD$ (100:1)	**S9**
**Figure S10.**	HSQC spectrum (400 MHz) of physacoztolide J (**10**) in $CDCl_3/CD_3OD$ (100:1)	**S10**
**Figure S11.**	HMBC spectrum (400 MHz) of physacoztolide J (**10**) in $CDCl_3/CD_3OD$ (100:1)	**S10**
**Figure S12.**	^{1}H NMR spectrum (400 MHz) of physacoztolide K (**11**) in $CDCl_3$	**S11**
**Figure S13.**	^{13}C NMR spectrum (100 MHz) of physacoztolide K (**11**) in $CDCl_3$	**S11**
**Figure S14.**	HSQC spectrum (400 MHz) of physacoztolide K (**11**) in $CDCl_3$	**S12**
**Figure S15.**	HMBC spectrum (400 MHz) of physacoztolide K (**11**) in $CDCl_3$	**S12**
**Figure S16.**	^{1}H-^{1}H COSY spectrum (400 MHz) of physacoztolide K (**11**) in $CDCl_3$	**S13**
**Figure S17.**	^{1}H and 1D NOESY spectra (400 MHz) of physacoztolide K (**11**) in $CDCl_3$	**S13**
**Figure S18.**	^{1}H NMR spectrum (400 MHz) of physacoztolide L (**12**) in $CDCl_3$	**S14**
**Figure S19.**	^{13}C NMR spectrum (100 MHz) of physacoztolide L (**12**) in $CDCl_3$	**S14**
**Figure S20.**	HSQC spectrum (400 MHz) of physacoztolide L (**12**) in $CDCl_3$	**S15**
**Figure S21.**	HMBC spectrum (400 MHz) of physacoztolide L (**12**) in $CDCl_3$	**S15**
**Figure S22.**	^{1}H-^{1}H COSY spectrum (400 MHz) of physacoztolide L (**12**) in $CDCl_3$	**S16**
**Figure S23.**	^{1}H and 1D NOESY spectra (400 MHz) of physacoztolide L (**12**) in $CDCl_3$	**S16**
**Figure S24.**	^{1}H NMR spectrum (400 MHz) of 28-hydroxyphysachenolide C (**13**) in CD_3OD	**S17**
**Figure S25.**	^{13}C NMR spectrum (100 MHz) of 28-hydroxyphysachenolide C (**13**) in CD_3OD	**S17**
**Figure S26.**	HSQC spectrum (400 MHz) of 28-hydroxyphysachenolide C (**13**) in CD_3OD	**S18**
**Figure S27.**	HMBC spectrum (400 MHz) of 28-hydroxyphysachenolide C (**13**) in CD_3OD	**S18**
**Figure S28.**	^{1}H NMR spectrum (400 MHz) of 15α-acetoxy-28-hydroxyphysachenolide C (**14**) in CD_3OD	**S19**
**Figure S29.**	^{13}C NMR spectrum (100 MHz) of 15α-acetoxy-28-hydroxyphysachenolide C (**14**) in CD_3OD	**S19**
**Figure S30.**	HSQC spectrum (400 MHz) of 15α-acetoxy-28-hydroxyphysachenolide C (**14**) in CD_3OD	**S20**
**Figure S31.**	HMBC spectrum (400 MHz) of 15α-acetoxy-28-hydroxyphysachenolide C (**14**) in CD_3OD	**S20**
**Figure S32.**	^{1}H NMR spectrum (400 MHz) of 28-oxophysachenolide C (**15**) in $CDCl_3$	**S21**
**Figure S33.**	^{13}C NMR spectrum (100 MHz) of 28-oxophysachenolide C (**15**) in $CDCl_3$	**S21**
**Figure S34.**	HSQC spectrum (400 MHz) of 28-oxophysachenolide C (**15**) in $CDCl_3$	**S22**
**Figure S35.**	HMBC spectrum (400 MHz) of 28-oxophysachenolide C (**15**) in $CDCl_3$	**S22**
**Figure S36.**	^{1}H NMR spectrum (400 MHz) of physacoztolide M (**16**) in $CDCl_3/CD_3OD$ (100:1)	**S23**
**Figure S37.**	^{13}C NMR spectrum (100 MHz) of physacoztolide M (**16**) in $CDCl_3/CD_3OD$ (100:1)	**S23**
**Figure S38.**	HSQC spectrum (400 MHz) of physacoztolide M (**16**) in $CDCl_3/CD_3OD$ (100:1)	**S24**
**Figure S39.**	HMBC spectrum (400 MHz) of physacoztolide M (**16**) in $CDCl_3/CD_3OD$ (100:1)	**S24**
**Figure S40.**	^{1}H NMR spectrum (400 MHz) of 5*α*-chloro-6*β*-hydroxy-5,6-dihydrophysachenolide D (**17**) in $CDCl_3$	**S25**
**Figure S41.**	^{13}C NMR spectrum (100 MHz) of 5*α*-chloro-6*β*-hydroxy-5,6-dihydrophysachenolide D (**17**) in $CDCl_3$	**S25**
**Figure S42.**	HSQC spectrum (400 MHz) of 5*α*-chloro-6*β*-hydroxy-5,6-dihydrophysachenolide D (**17**) in $CDCl_3$	**S26**
**Figure S43.**	HMBC spectrum (400 MHz) of 5*α*-chloro-6*β*-hydroxy-5,6-dihydrophysachenolide D (**17**) in $CDCl_3$	**S26**

Figure S44. ^{1}H and 1D NOESY spectra (400 MHz) of 5*α*-chloro-6*β*-hydroxy-5,6-dihydrophysachenolide D (**17**) in $CDCl_3$ **S27**
Figure S45. ^{1}H NMR spectrum (400 MHz) of 15*α*-acetoxy-5*α*-chloro-6*β*-hydroxy-5,6-dihydrophysachenolide D (**18**) in $CDCl_3$ **S27**
Figure S46. ^{13}C NMR spectrum (100 MHz) 15*α*-acetoxy-5*α*-chloro-6*β*-hydroxy-5,6-dihydrophysachenolide D (**18**) in $CDCl_3$ **S28**
Figure S47. HSQC spectrum (400 MHz) 15*α*-acetoxy-5*α*-chloro-6*β*-hydroxy-5,6-dihydrophysachenolide D (**18**) in $CDCl_3$ **S28**
Figure S48. HMBC spectrum (400 MHz) 15*α*-acetoxy-5*α*-chloro-6*β*-hydroxy-5,6-dihydrophysachenolide D (**18**) in $CDCl_3$ **S29**
Figure S49. ^{1}H and 1D NOESY spectra (400 MHz) of 15*α*-acetoxy-5*α*-chloro-6*β*-hydroxy-5,6-dihydrophysachenolide D (**18**) in $CDCl_3$ **S29**
Figure S50. ^{1}H NMR spectrum (400 MHz) of 28-hydroxy-5*α*-chloro-6*β*-hydroxy-5,6-dihydrophysachenolide D (**19**) in $CDCl_3$ **S30**
Figure S50. ^{1}H NMR spectrum (400 MHz) of 28-hydroxy-5*α*-chloro-6*β*-hydroxy-5,6-dihydrophysachenolide D (**19**) in CD_3OD **S30**
Figure S51. ^{13}C NMR spectrum (100 MHz) of 28-hydroxy-5*α*-chloro-6*β*-hydroxy-5,6-dihydrophysachenolide D (**19**) in $CDCl_3$ **S31**
Figure S51. ^{13}C NMR spectrum (100 MHz) of 28-hydroxy-5*α*-chloro-6*β*-hydroxy-5,6-dihydrophysachenolide D (**19**) in CD_3OD **S31**
Figure S52. HSQC spectrum (400 MHz) of 28-hydroxy-5*α*-chloro-6*β*-hydroxy-5,6-dihydrophysachenolide D (**19**) in CD_3OD **S32**
Figure S53. HMBC spectrum (400 MHz) 28-hydroxy-5*α*-chloro-6*β*-hydroxy-5,6-dihydrophysachenolide D (**19**) in CD_3OD **S32**
Figure S54. ^{1}H-^{1}H COSY spectrum (400 MHz) of 28-hydroxy-5*α*-chloro-6*β*-hydroxy-5,6-dihydrophysachenolide D (**19**) in CD_3OD **S33**
Figure S55. ^{1}H NMR spectrum (400 MHz) of physachenolide A-5-methyl ether (**20**) in $CDCl_3/CD_3OD$ (100:1) **S33**
Figure S56. ^{13}C NMR spectrum (100 MHz) of physachenolide A-5-methyl ether (**20**) in $CDCl_3/CD_3OD$ (100:1) **S34**
Figure S57. HSQC spectrum (400 MHz) of physachenolide A-5-methyl ether (**20**) in $CDCl_3/CD_3OD$ (100:1) **S34**
Figure S58. HMBC spectrum (400 MHz) of physachenolide A-5-methyl ether (**20**) in $CDCl_3/CD_3OD$ (100:1) **S35**
Figure S59. ECD spectra of **9–20** **S36**
Figure S60. Key HMBC correlations of **9–20** and Key H-H COSY correlations of **11**, **12**, **19**, and **20** **S37**
Figure S61. Key NOESY correlations of **11**, **12**, **17,** and **18** **S38**
Figure S62. Investigation of products formed on exposure of physachenolide C (**8**) to mild acidic conditions by HPLC **S39**

Figure S1. Aeroponic cultivation of plants of Solanaceae. (**A**) Schematic of an aeroponic cultivation set-up (for details, see description below); (**B**) images showing *Withania somnifera* growing under aeroponic conditions in a greenhouse, and (**C**) roots of *W. somnifera* hanging inside the aeroponic chamber which are sprayed intermittently with the nutrient medium (see Table S1, below).

A **B** **C**

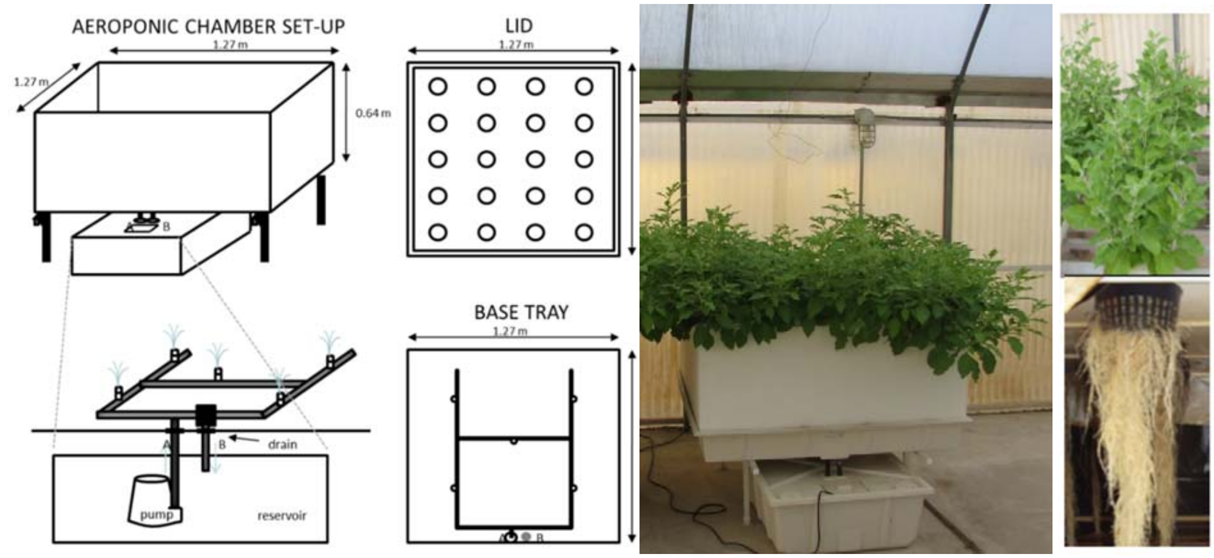

Procedure: For aeroponic cultivation, plants were grown on top of chambers which measured 1.27 m x 1.27 m x 0.64 m (W x L x H), and equipped with five nozzles powered by a pump to spray nutrient solution every 4 min for a period of 1 min (**Figure S1A**). A reservoir of 150 L of nutrient solution was maintained under each chamber accommodating 20 plants. This nutrient solution was prepared according to a modified Hoagland hydroponic recipe having a pH of 5.8 – 6.0 (**Table S1**).

Table S1. Constitution of the modified Hoagland's nutrient medium providing macro- and micro-nutrients for aeroponic plant cultivation.

Macronutrients	g/L	Required Elements	Micronutrients	g/L	Required Elements
$Ca(NO_3)_2$ $4H_2O$	0.579	Calcium/Nitrogen	H_3BO_3	0.003	Boron
KNO_3[a]	0.515	Potassium/Nitrogen	$CuSO_4$ 20%	0.003	Copper
KH_2PO_4	0.204	Potassium/Phosphorous	$MnSO_4$ H_20 20%	0.004	Manganese
K_2SO_4	0.193	Potassium/Sulfur	Na_2MoO_4 $2H_20$	0.001	Molybdenum
$MgSO_4$ $7H_2O$	0.600	Magnesium/Sulfur	$ZnSO_4$ $7H_2O$ 20%	0.004	Zinc
$CaCl_2$ $6H_20$	0.278	Calcium/Chlorine			
Fe-EDDHA 10%	0.030	Iron			

[a]optional supplement used during plant reproductive phase.

Figure S2. Aeroponic cultivation of *Withania somnifera*. (**A**) Aeroponically-grown *W. somnifera* (Solanaceae) showing improved plant growth compared to (**B**) soil-grown *W. somnifera* provided with the same nutrient medium and grown for the same length of time. (**C**) Comparison of leaf size of *W. somnifera* showing that the leaves of aeroponically grown plants are about five times as large as those of soil grown plants. (**D**) Unusual withanolides, 3α-(uracil-1-yl)-2,3-dihydrowithaferin A and 3β-(adenin-9-yl)-2,3-dihydrowithaferin, isolated from aeroponically-grown *W. somnifera*.

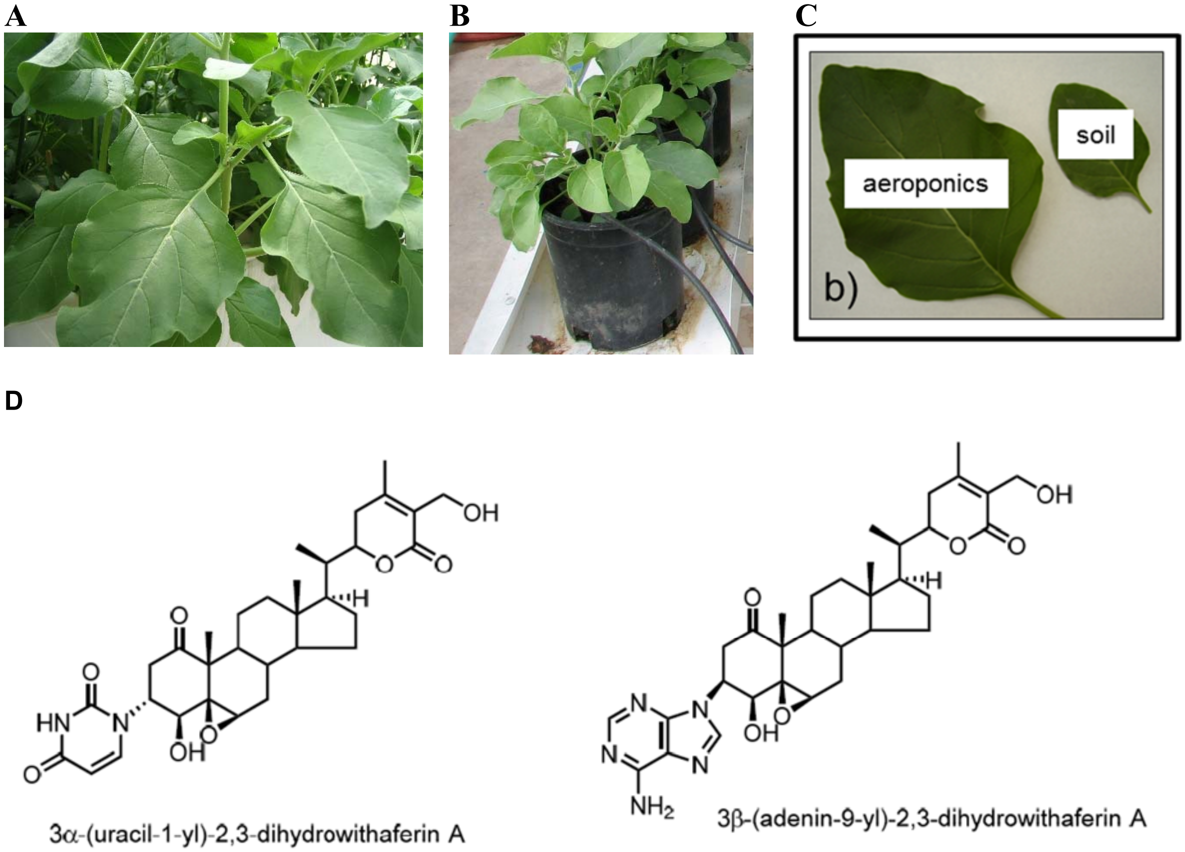

Figure S3. Aeroponic cultivation of *Physalis crassifolia*. (**A**) Aeroponically-grown *P. crassifolia* (Solanaceae) showing improved plant growth compared to (**B**) soil-grown *P. crassifolia* provided with the same nutrient medium and grown for the same length of time in the same greenhouse. (**C**) Comparison of HPLC profiles of withanolide containing extracts of soil-grown and aeroponically-grown *P. crassifolia* (red arrows indicate additional HPLC peaks present in aeroponically-grown plant). (**D**) Structures of withanolides occurring in wild-crafted/soil-grown *P. crassifolia*, and (**E**) structures of 14 additional withanolides, including 11 new withanolides (names in red color) together with 4 withanolides also encountered in wild-crafted soil-grown plant (names in blue color), and 3 previously known withanolides (names in black color) occurring in aeroponically-grown *P. crassifolia*.

A

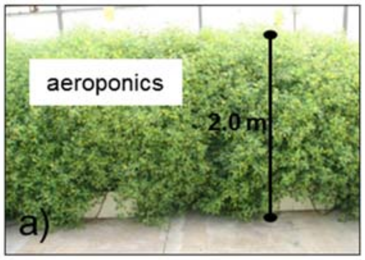

B

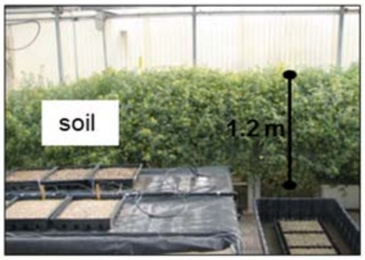

C

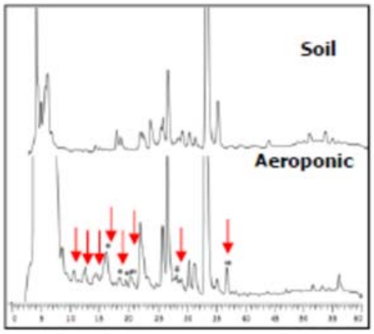

<u>HPLC analysis of extracts derived from P. crassifolia cultivated in soil and under aeroponic conditions:</u> Dried and powdered aerial parts of *P. crassifolia* (1.0 g) obtained from soil and aeroponic cultivation were separately extracted with MeOH (10 mL) under sonication in an ultrasonic bath for 2 h. After filtration, each extract was evaporated to dryness and the resulting residues were dissolved in MeOH (1.0 mL). The insoluble material was removed by centrifugation at 10,000 rpm for 5 min, and the supernatant was used directly for HPLC analysis.

D

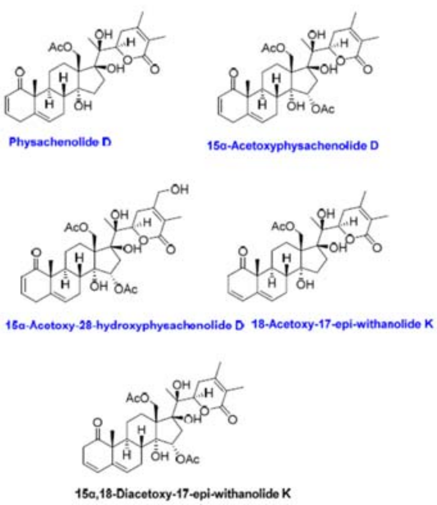

E

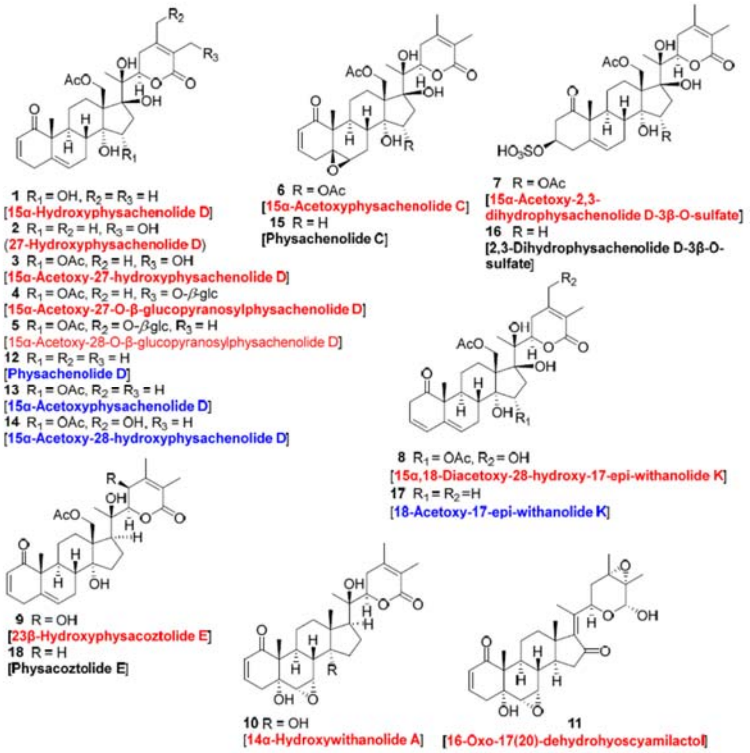

Figure S4. ^{1}H NMR spectrum (400 MHz) of physacoztolide I (**9**) in $CDCl_3/CD_3OD$ (100:1)

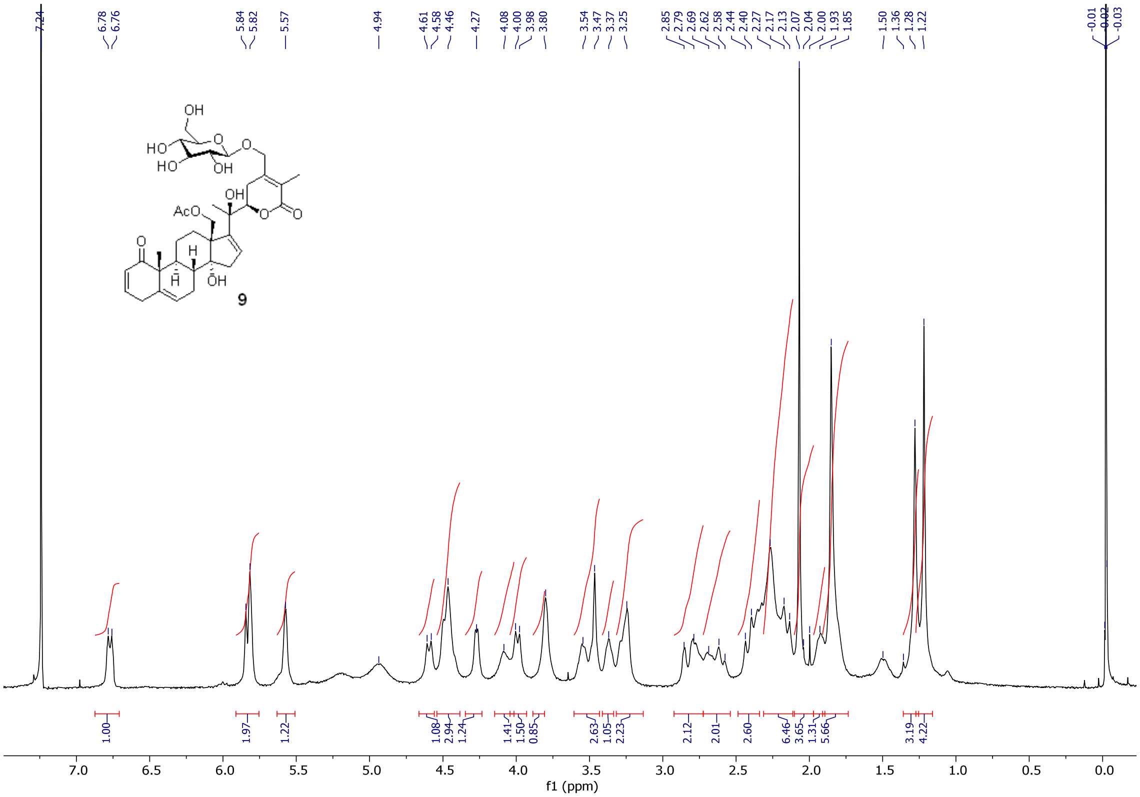

Figure S5. ^{13}C NMR spectrum (100 MHz) of physacoztolide I (**9**) in $CDCl_3/CD_3OD$ (100:1)

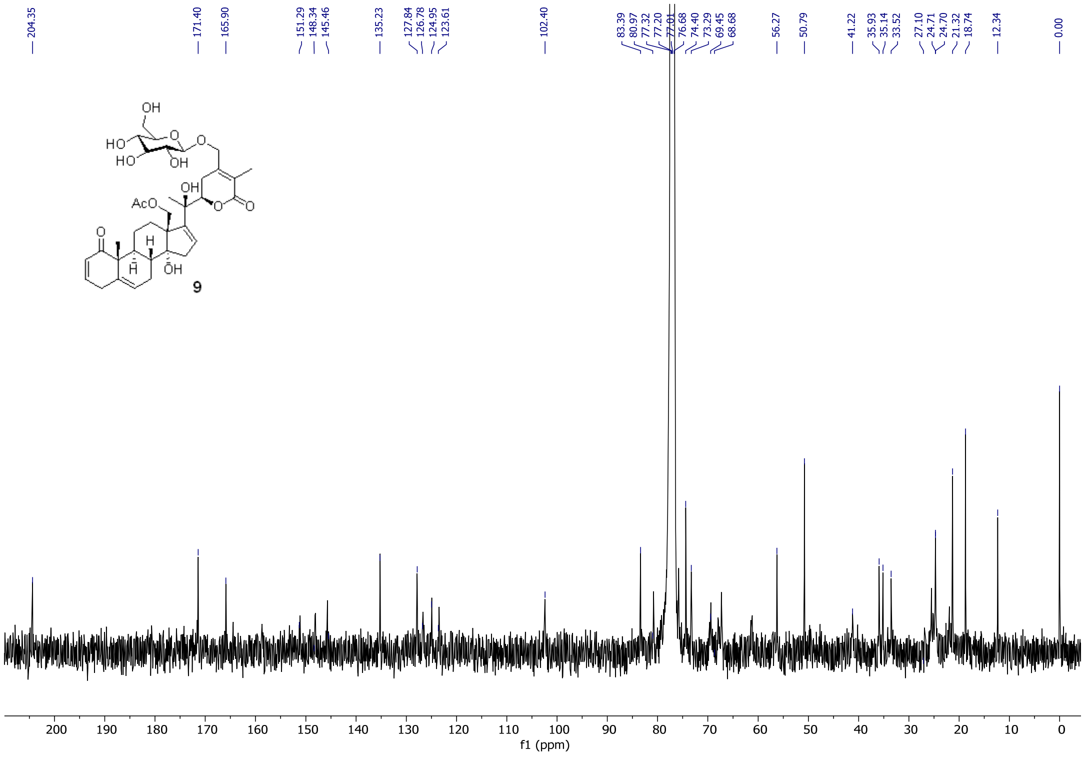

Figure S6. HSQC spectrum (400 MHz) of physacoztolide I (**9**) in $CDCl_3/CD_3OD$ (100:1)

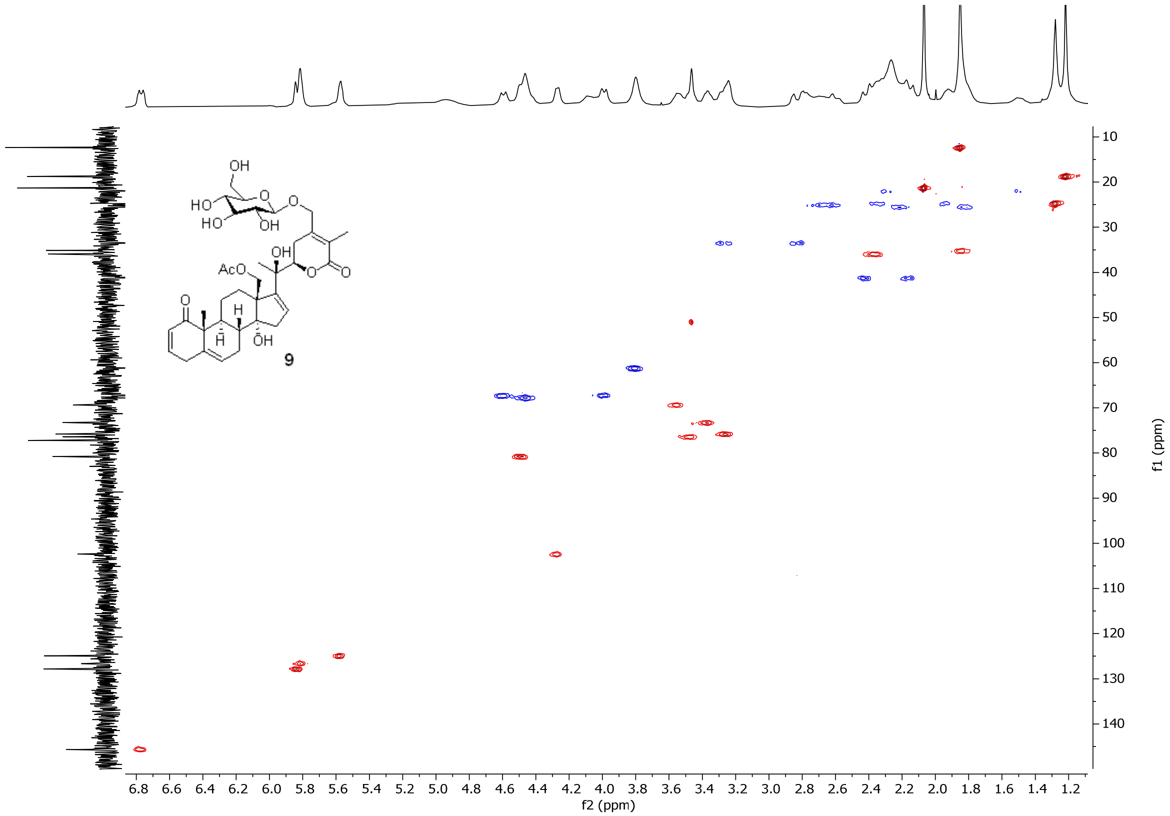

Figure S7. HMBC spectrum (400 MHz) of physacoztolide I (**9**) in $CDCl_3/CD_3OD$ (100:1)

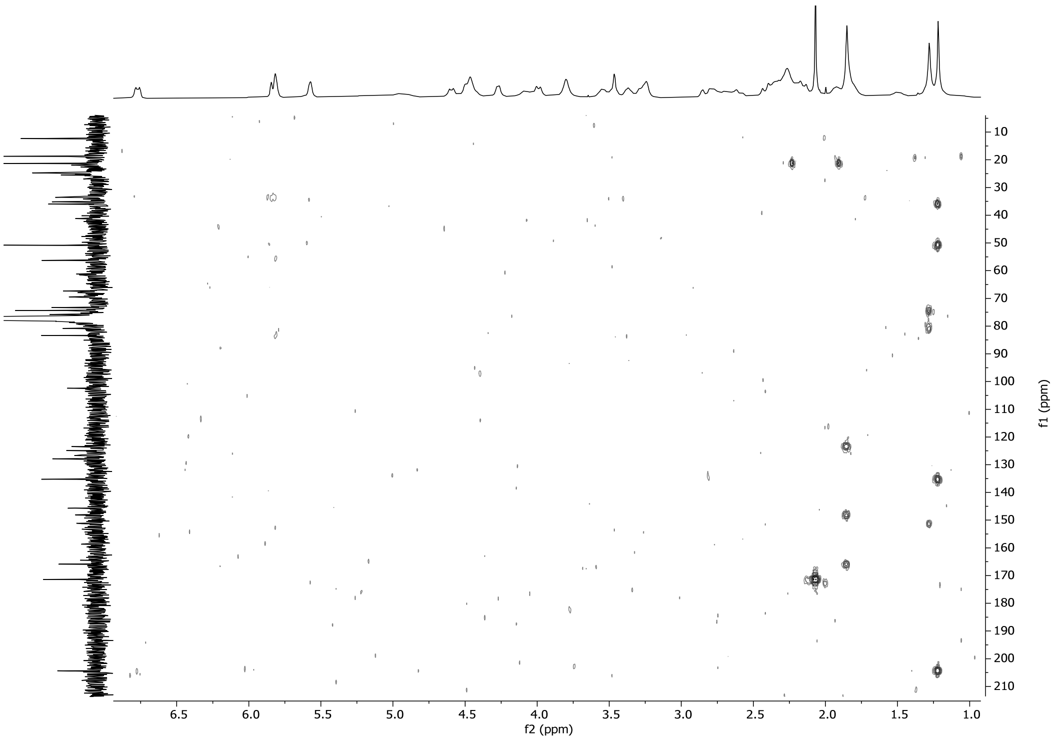

Figure S8. ^{1}H NMR spectrum (400 MHz) of physacoztolide J (**10**) in $CDCl_3/CD_3OD$ (100:1)

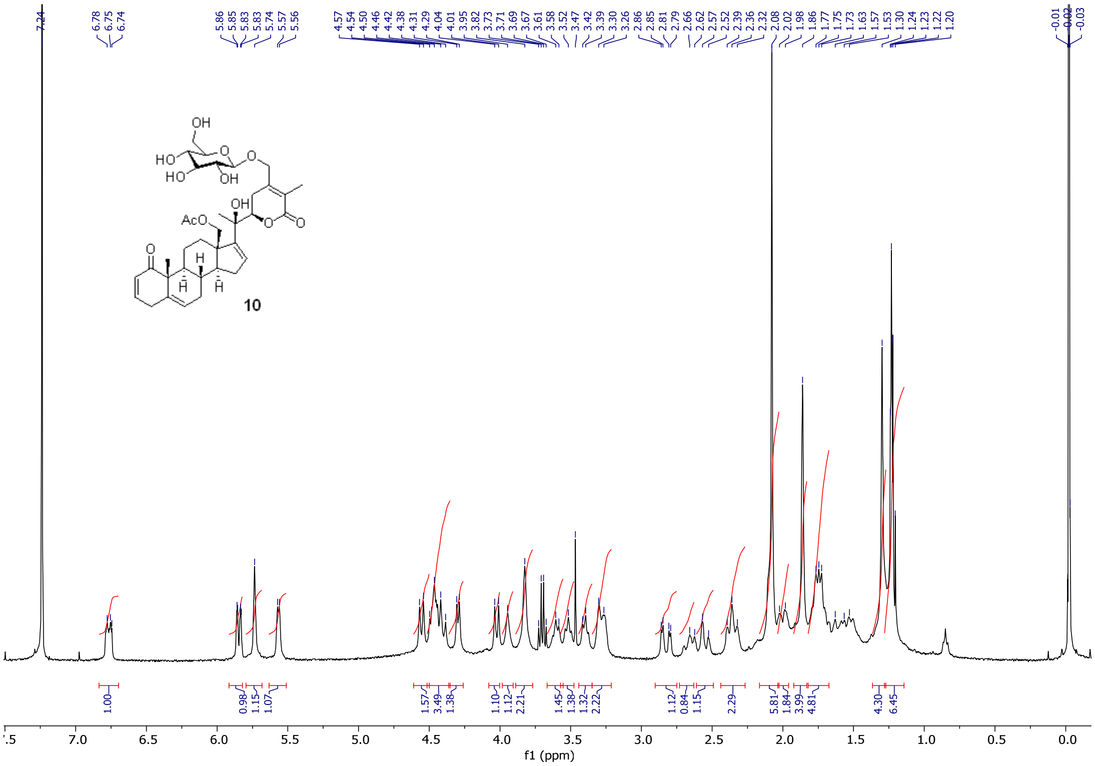

Figure S9. ^{13}C NMR spectrum (100 MHz) of physacoztolide J (**10**) in $CDCl_3/CD_3OD$ (100:1)

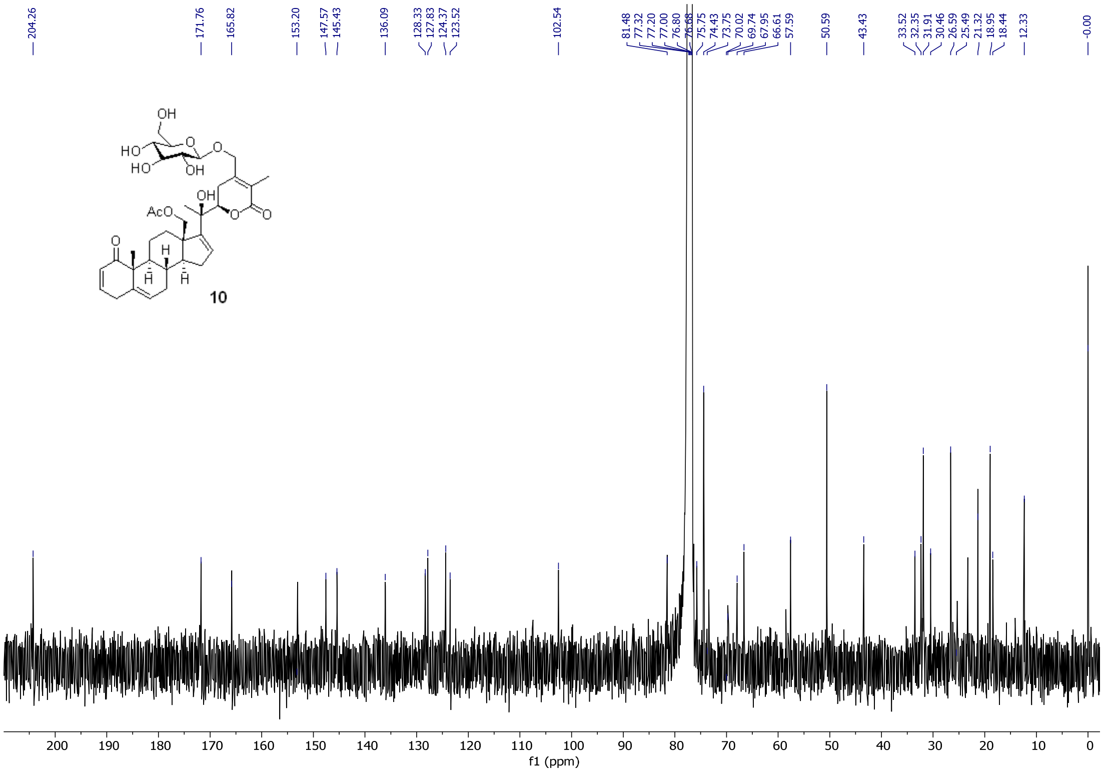

Figure S10. HSQC spectrum (400 MHz) of physacoztolide J (**10**) in $CDCl_3$/CD_3OD (100:1)

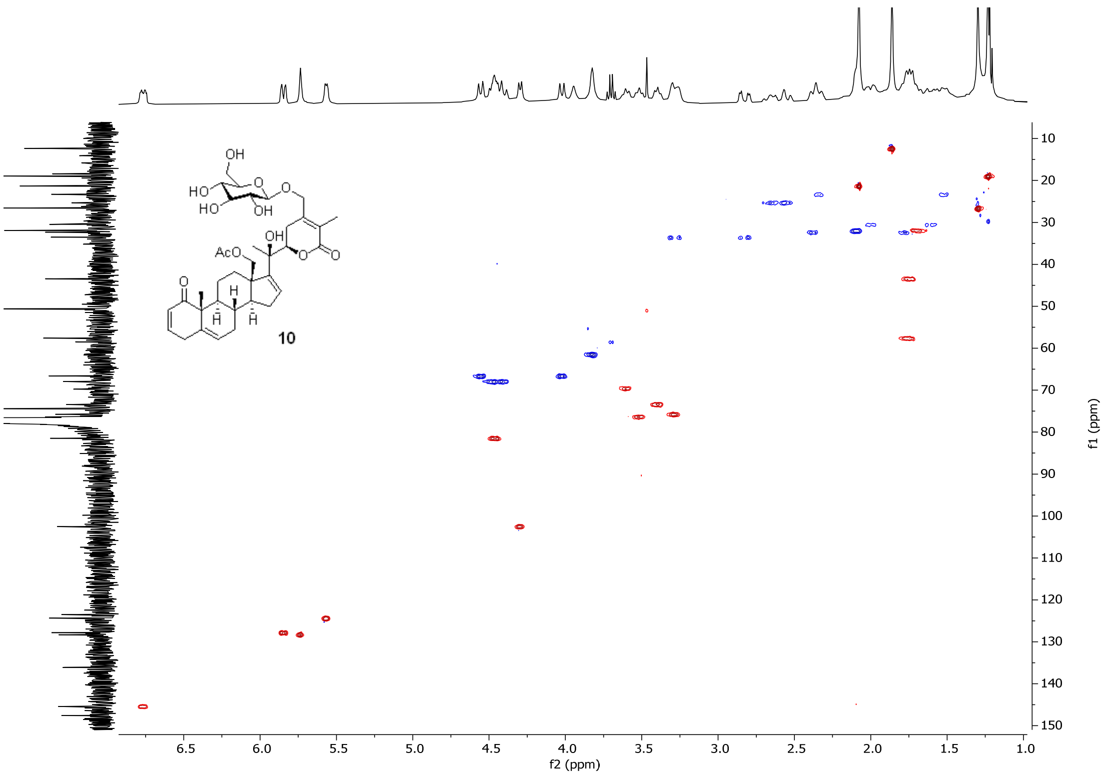

Figure S11. HMBC spectrum (400 MHz) of physacoztolide J (**10**) in $CDCl_3$/CD_3OD (100:1)

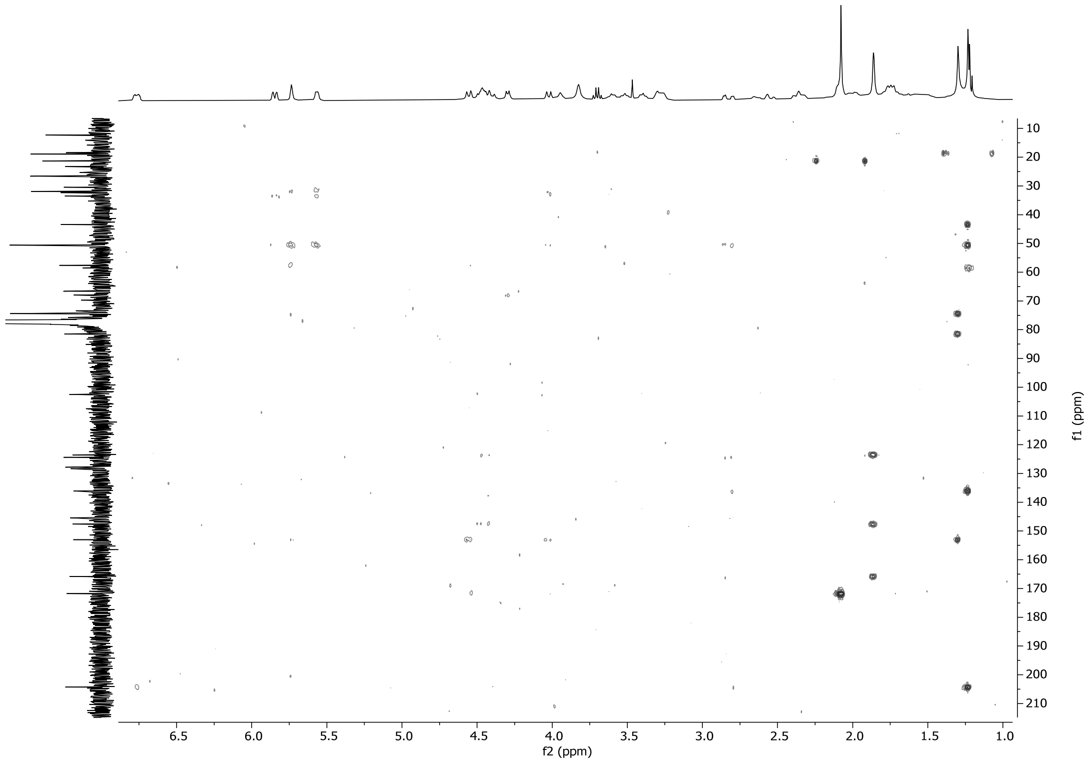

Figure S12. ^{1}H NMR spectrum (400 MHz) of physacoztolide K (**11**) in $CDCl_3$

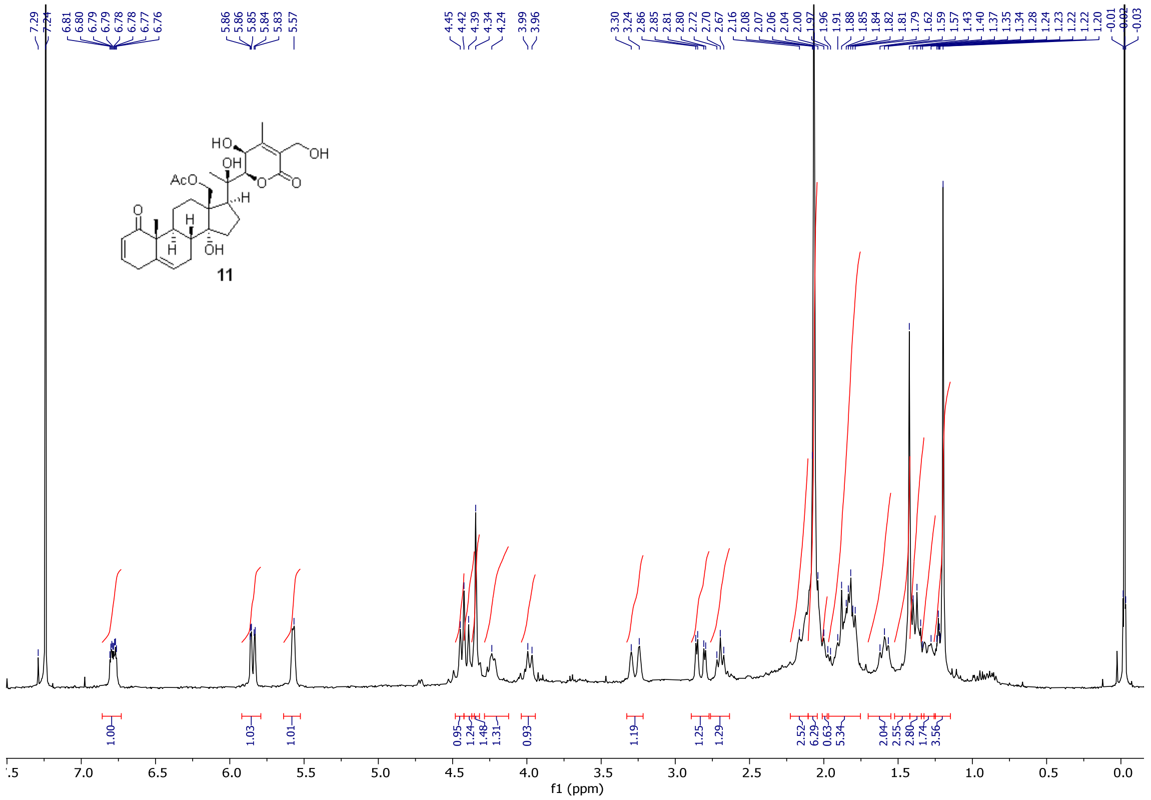

Figure S13. ^{13}C NMR spectrum (100 MHz) of physacoztolide K (**11**) in $CDCl_3$

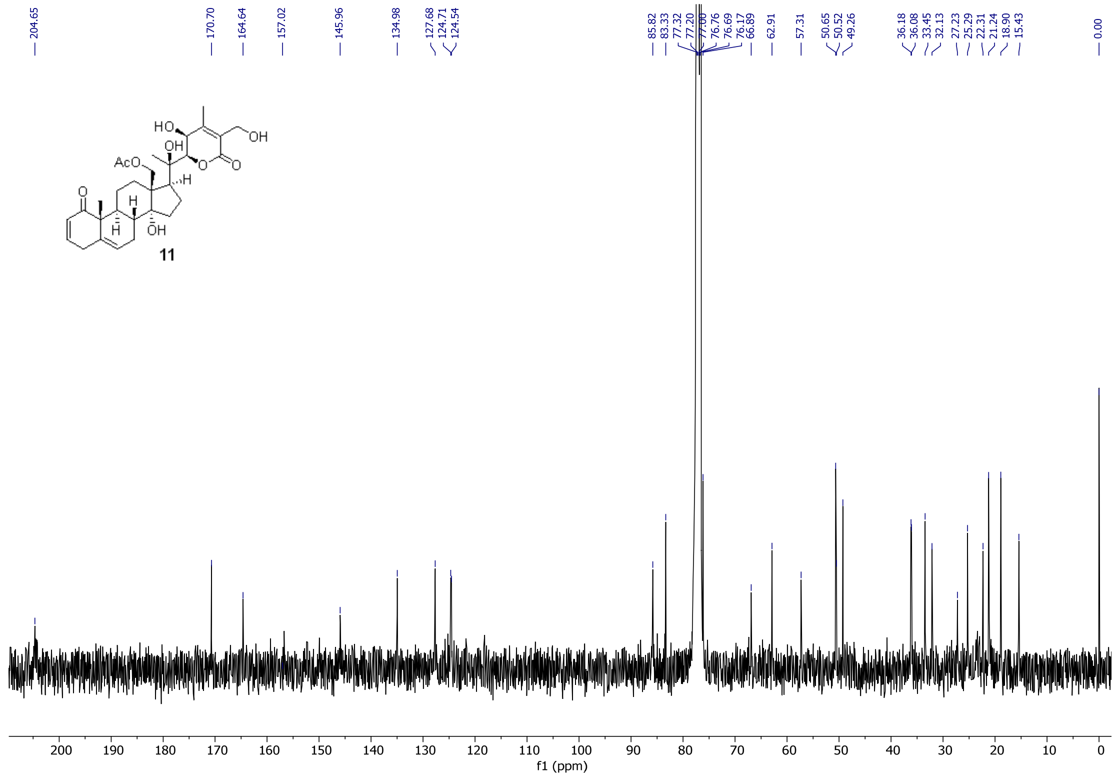

Figure S14. HSQC spectrum (400 MHz) of physacoztolide K (**11**) in $CDCl_3$

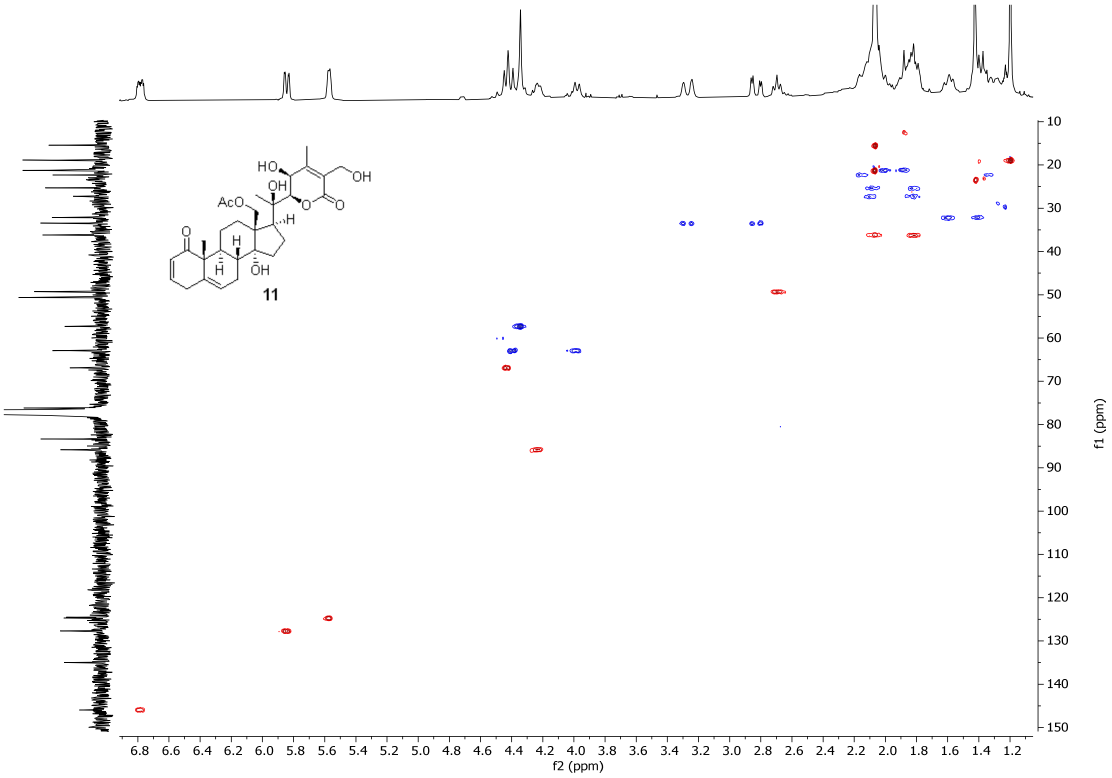

Figure S15. HMBC spectrum (400 MHz) of physacoztolide K (**11**) in $CDCl_3$

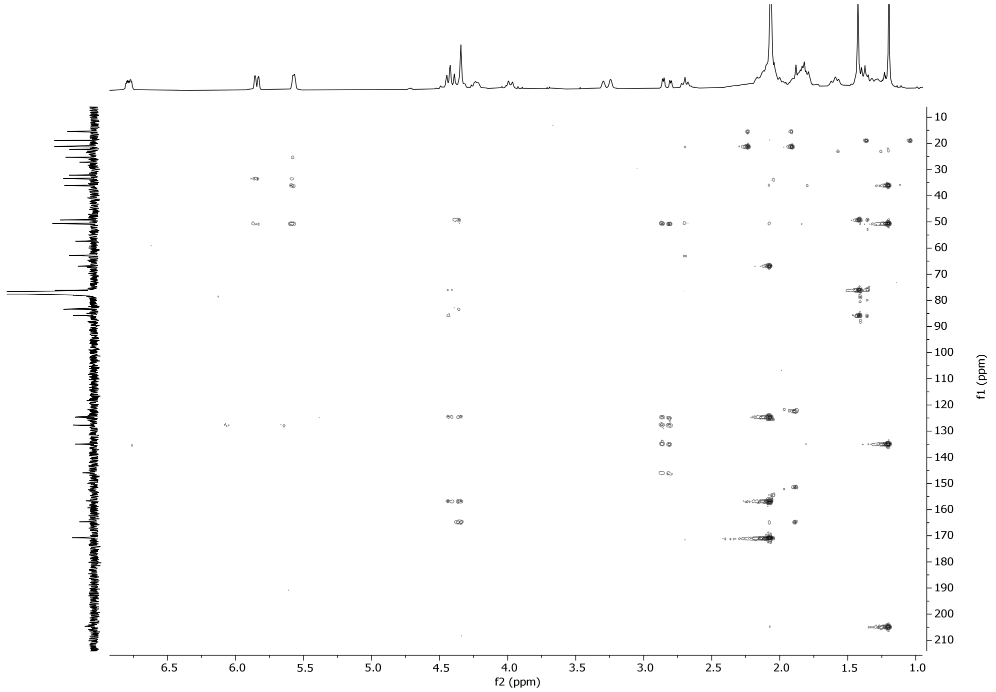

Figure S16. ^{1}H-^{1}H COSY spectrum (400 MHz) of physacoztolide K (**11**) in $CDCl_3$

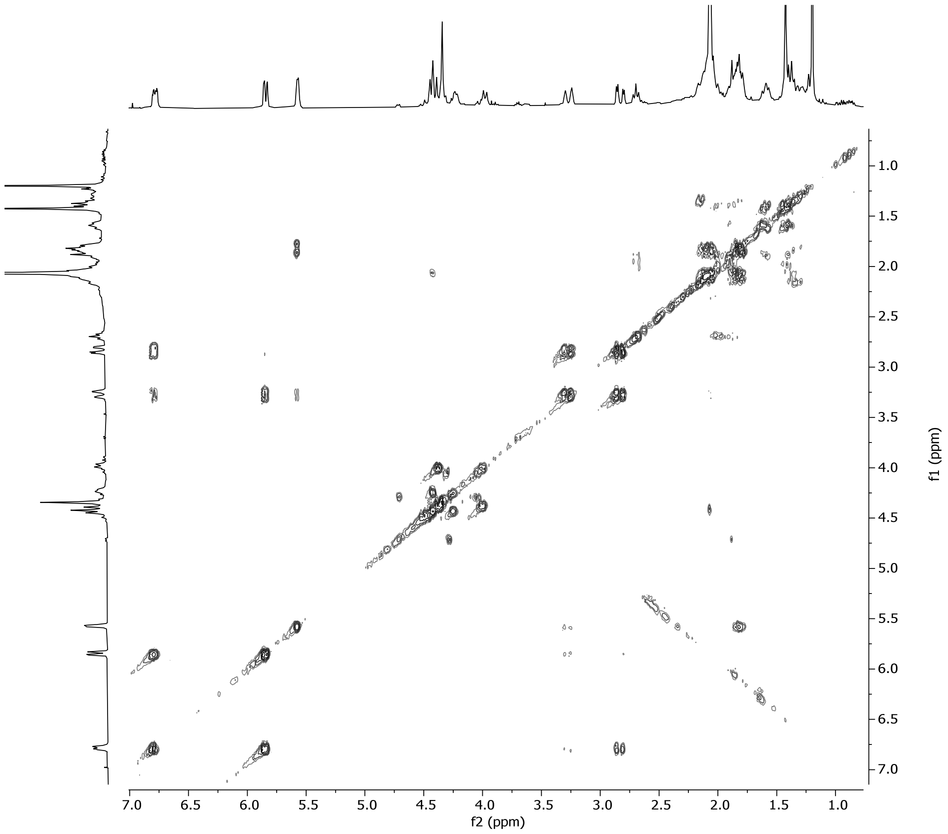

Figure S17. ^{1}H and 1D NOESY spectra (400 MHz) of physacoztolide K (**11**) in $CDCl_3$

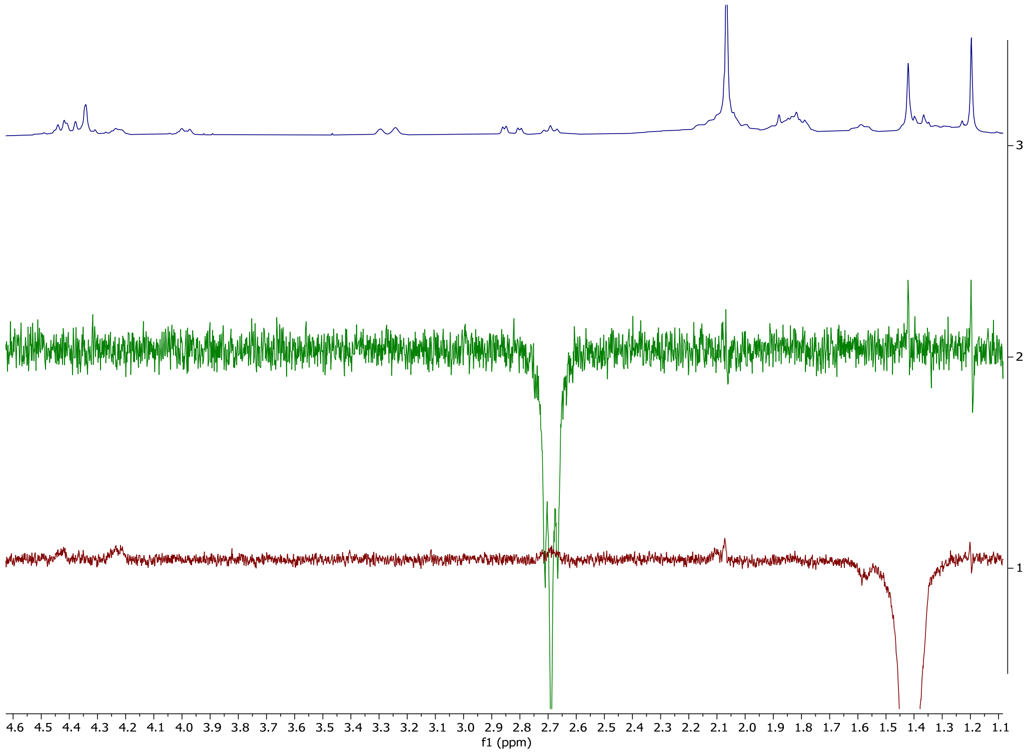

Figure S18. ^{1}H NMR spectrum (400 MHz) of physacoztolide L (**12**) in $CDCl_3$

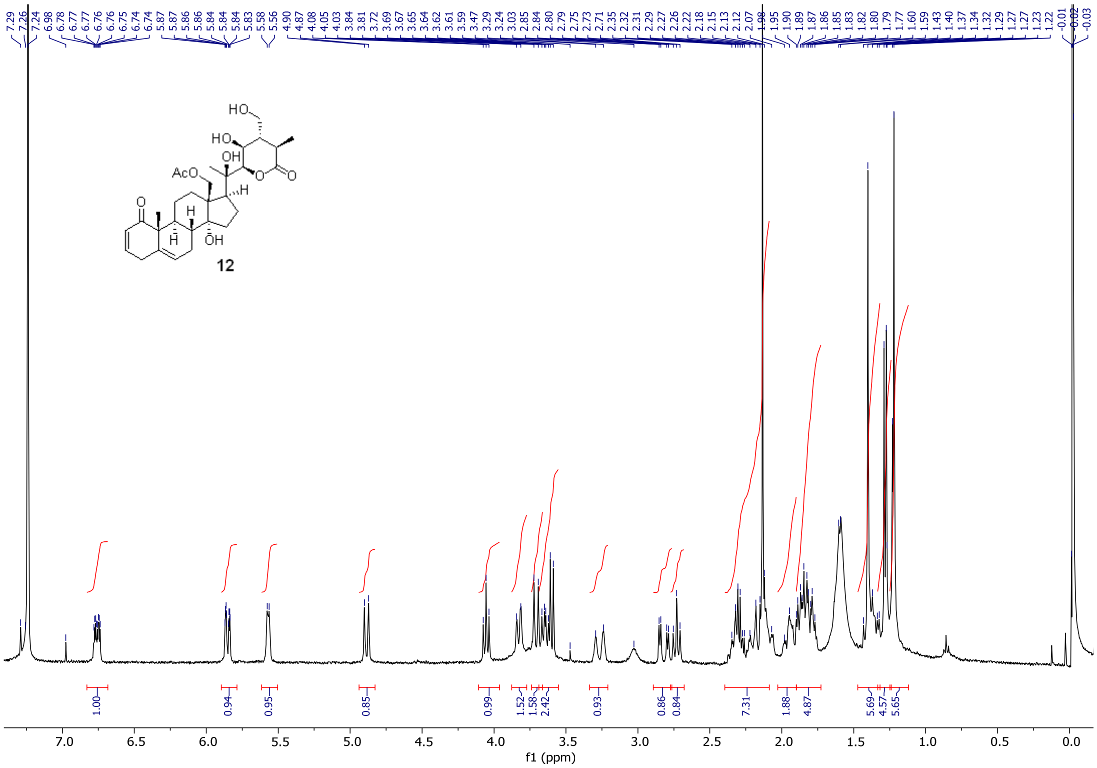

Figure S19. ^{13}C NMR spectrum (100 MHz) of physacoztolide L (**12**) in $CDCl_3$

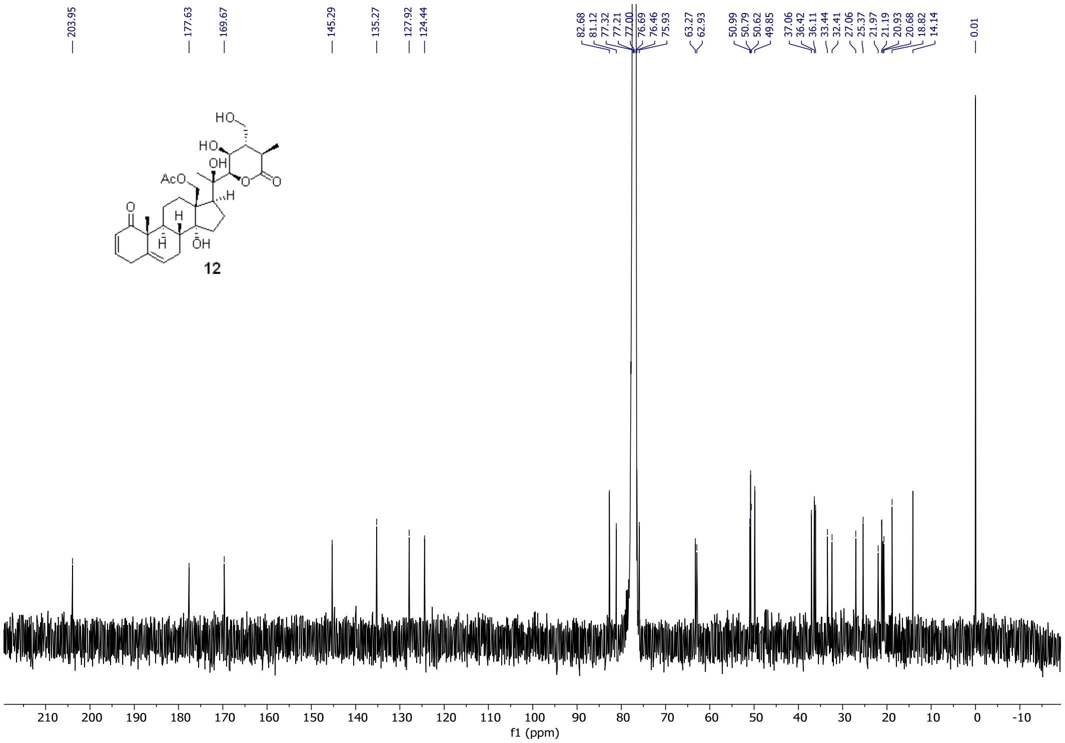

Figure S20. HSQC spectrum (400 MHz) of physacoztolide L (**12**) in $CDCl_3$

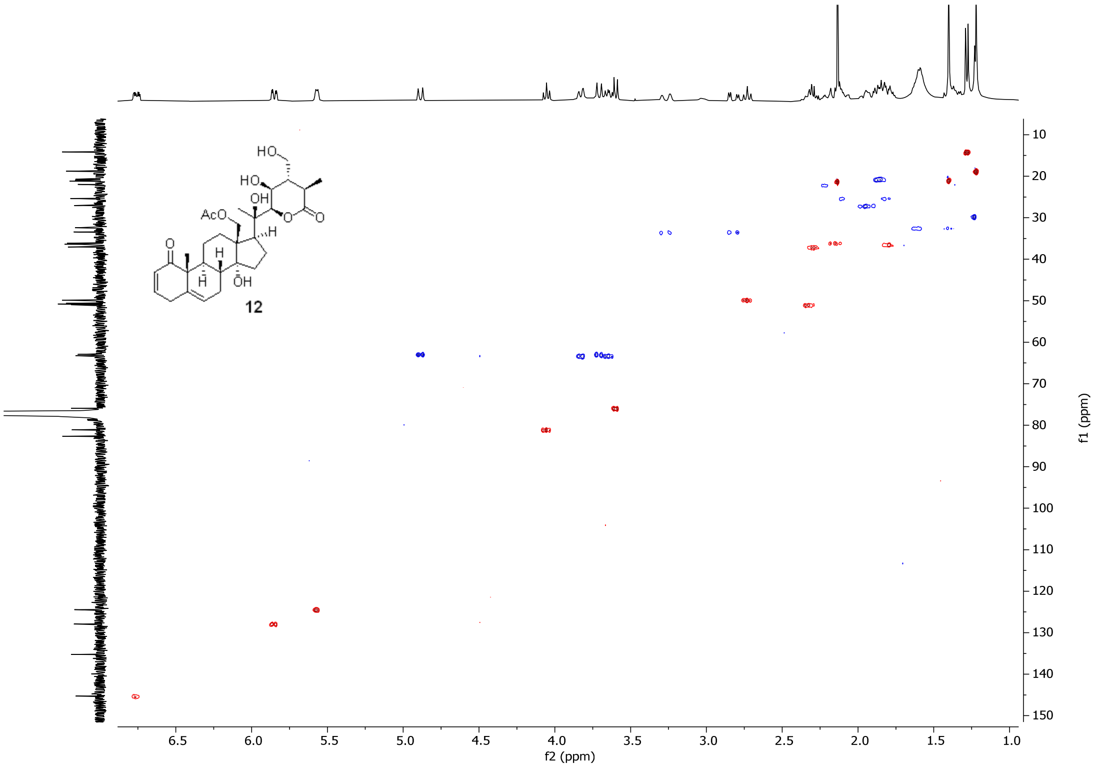

Figure S21. HMBC spectrum (400 MHz) of physacoztolide L (**12**) in $CDCl_3$

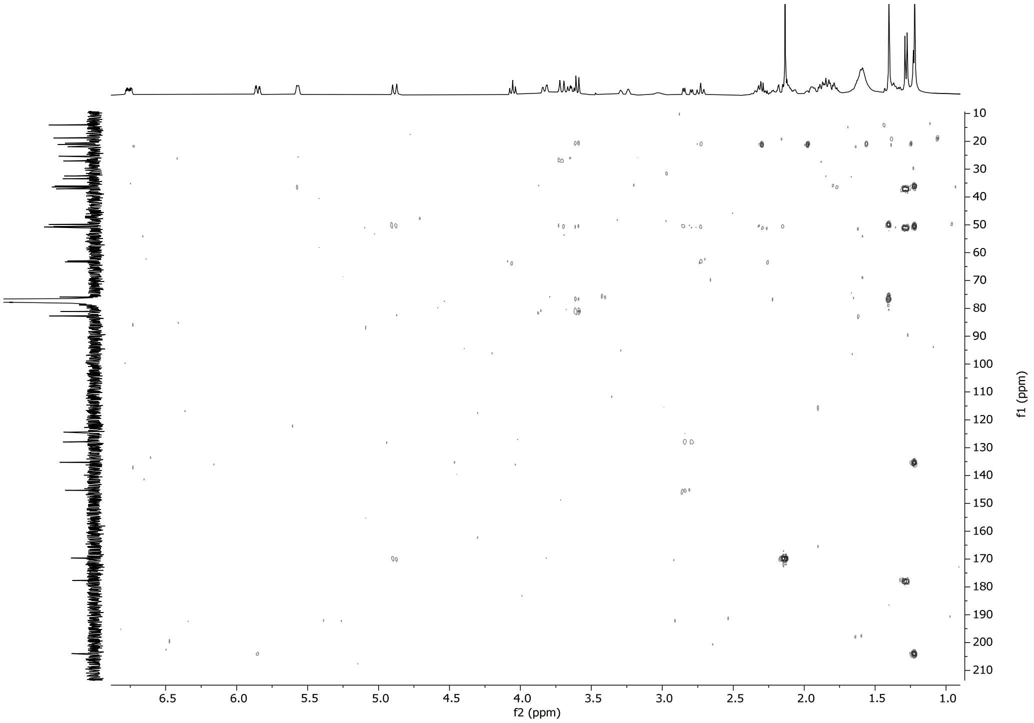

Figure S22. ^{1}H-^{1}H COSY spectrum (400 MHz) of physacoztolide L (**12**) in $CDCl_3$

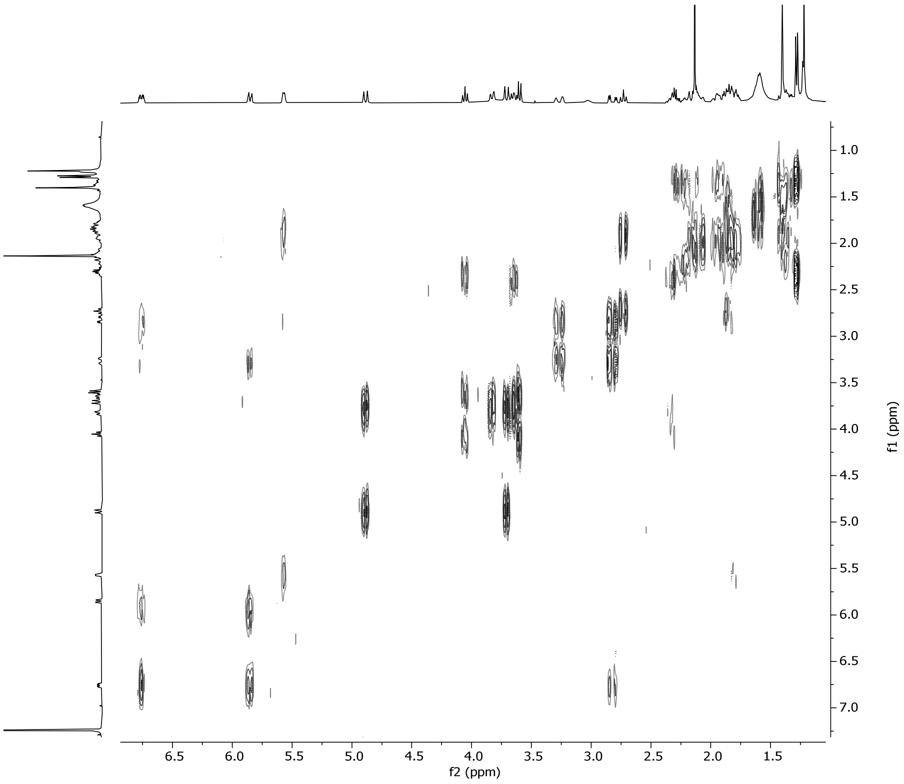

Figure S23. ^{1}H and 1D NOESY spectra (400 MHz) of physacoztolide L (**12**) in $CDCl_3$

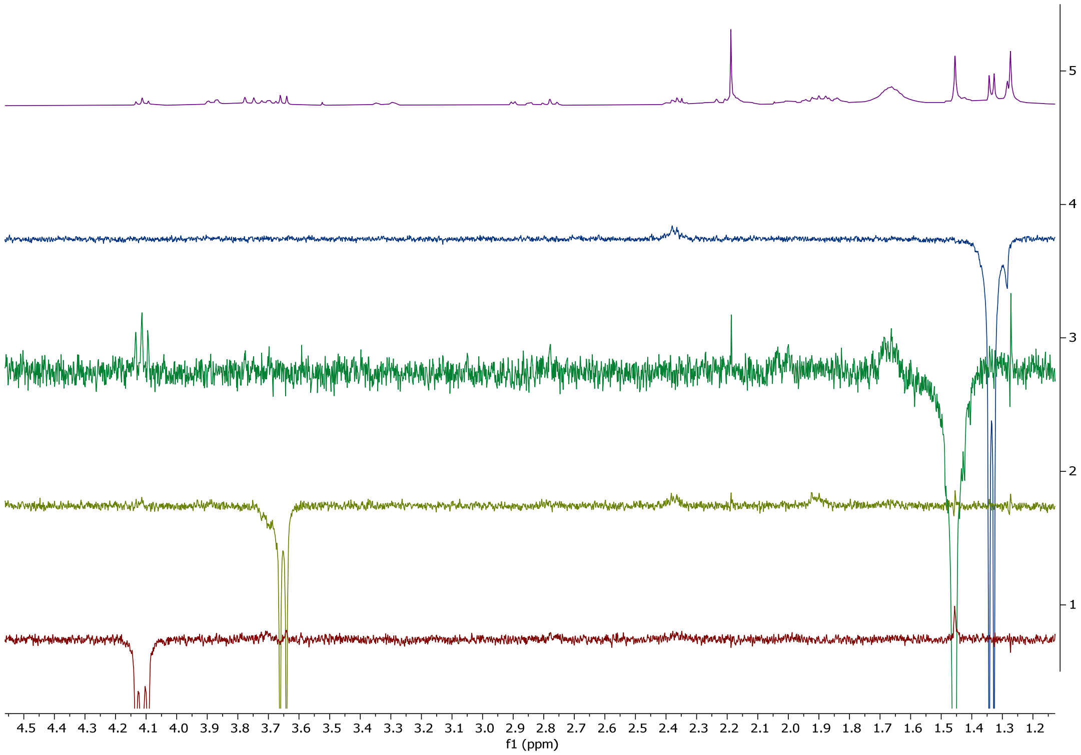

Figure S24. ^{1}H NMR spectrum (400 MHz) of 28-hydroxyphysachenolide C (**13**) in CD_3OD

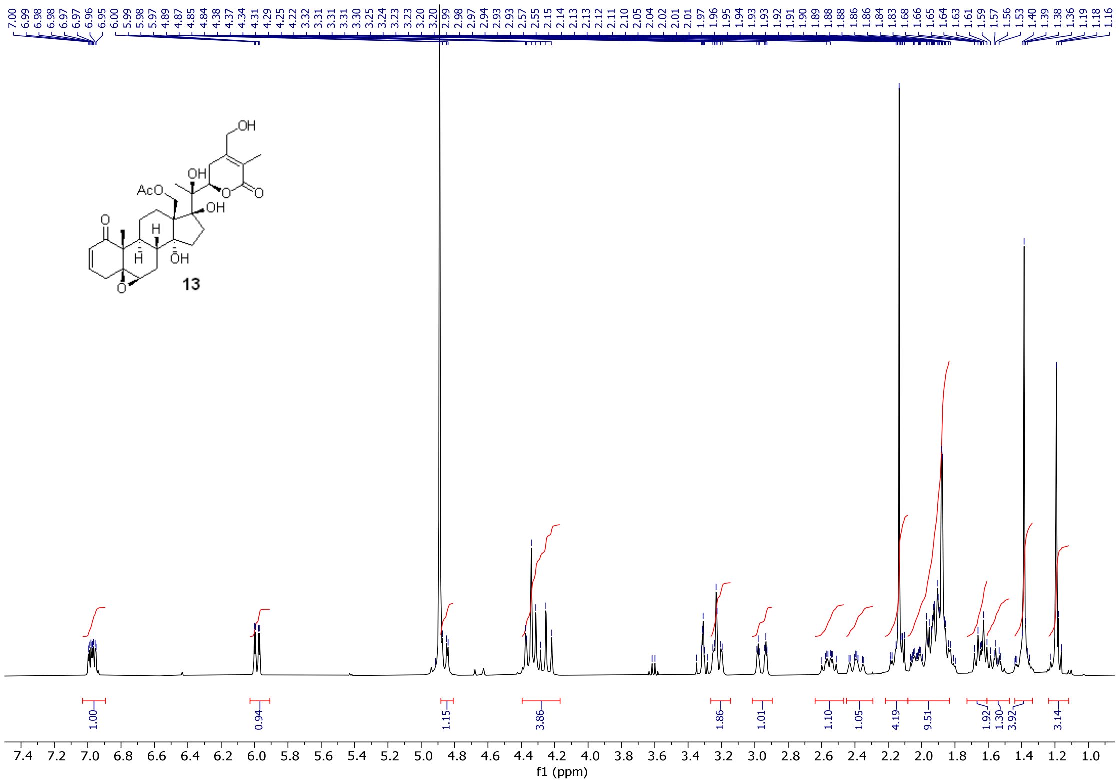

Figure S25. ^{13}C NMR spectrum (100 MHz) of 28-hydroxyphysachenolide C (**13**) in CD_3OD

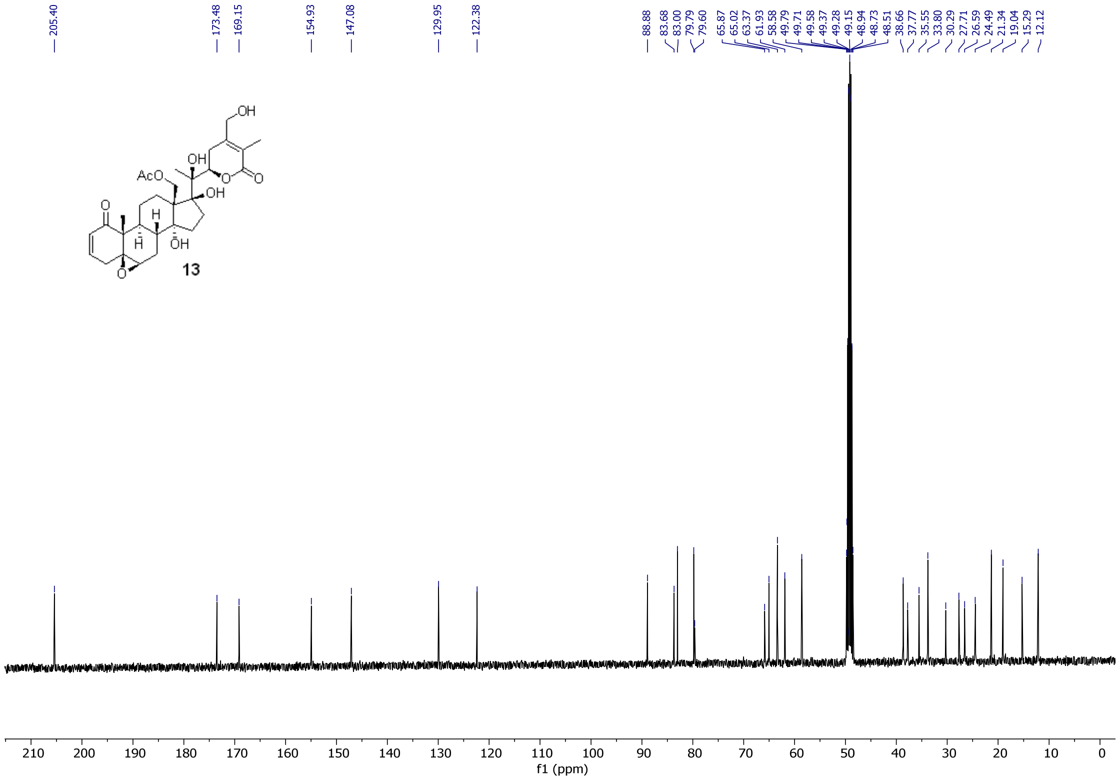

Figure S26. HSQC spectrum (400 MHz) of 28-hydroxyphysachenolide C (**13**) in CD_3OD

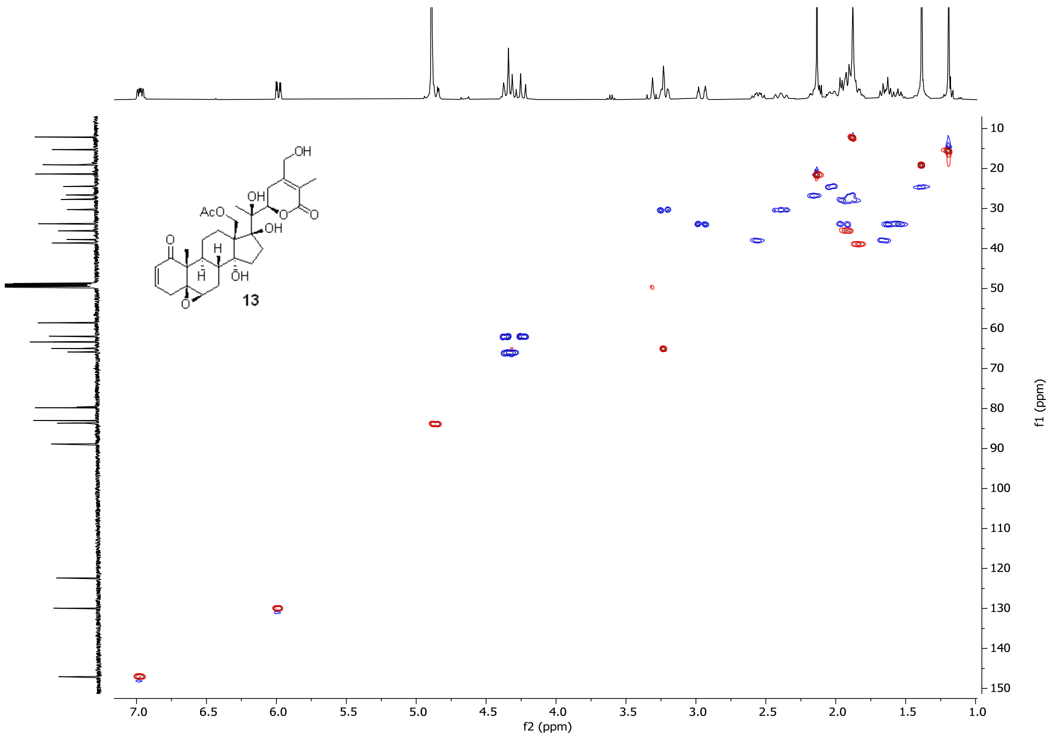

Figure S27. HMBC spectrum (400 MHz) of 28-hydroxyphysachenolide C (**13**) in CD_3OD

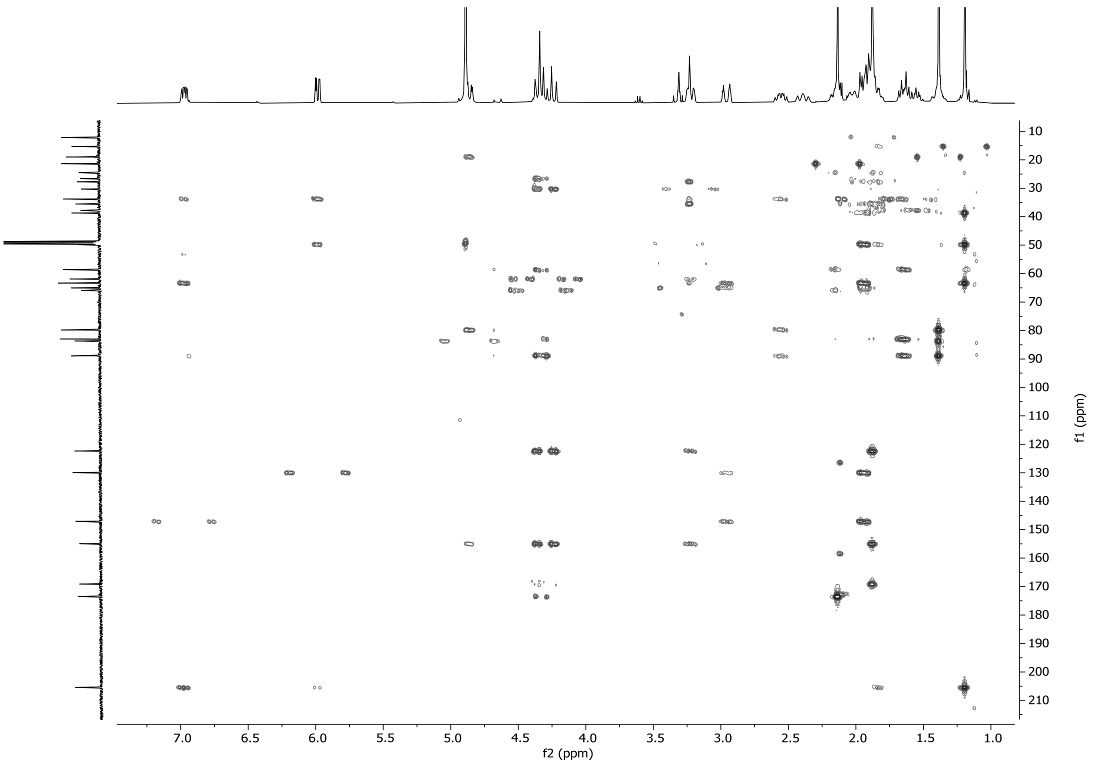

Figure S28. ^{1}H NMR spectrum (400 MHz) of 15α-acetoxy-28-hydroxyphysachenolide C (**14**) in CD_3OD

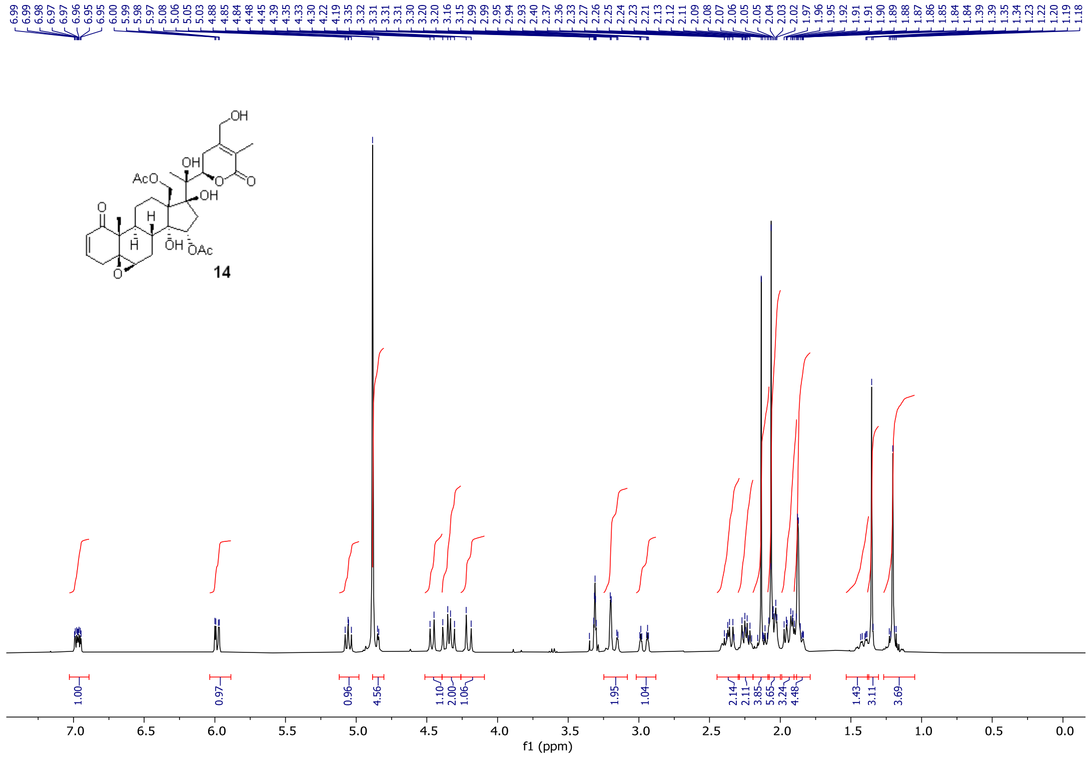

Figure S29. ^{13}C NMR spectrum (100 MHz) of 15α-acetoxy-28-hydroxyphysachenolide C (**14**) in CD_3OD

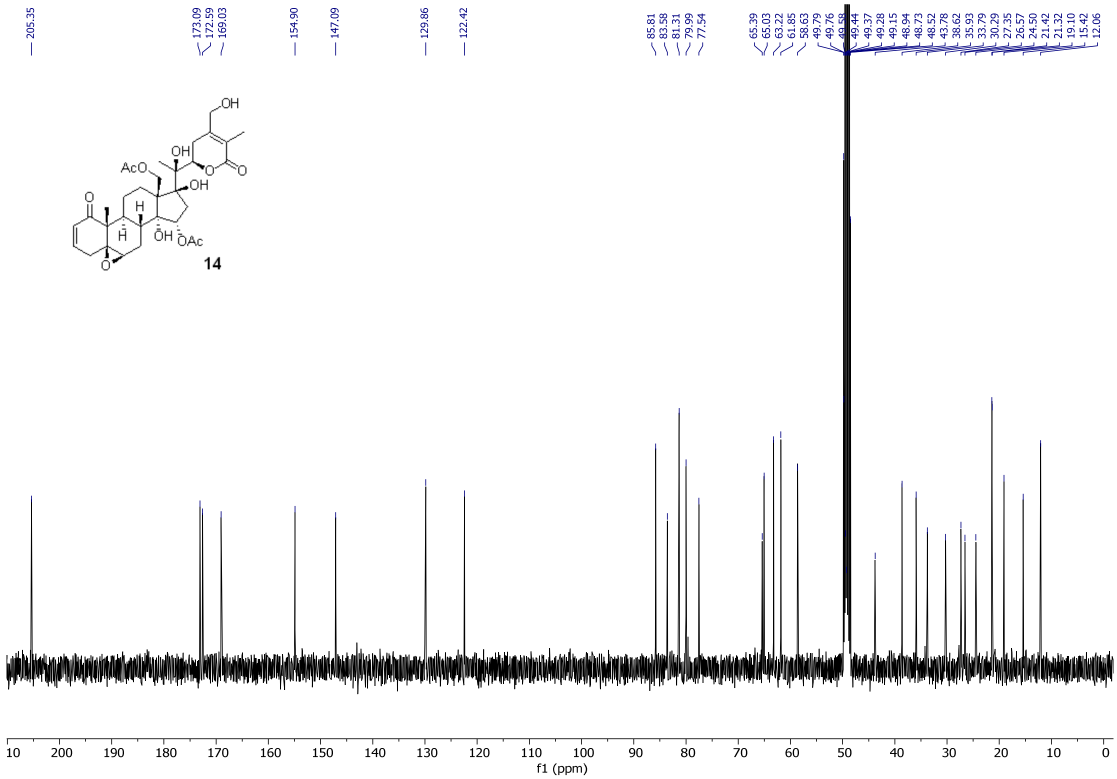

Figure S30. HSQC spectrum (400 MHz) of 15α-acetoxy-28-hydroxyphysachenolide C (**14**) in CD_3OD

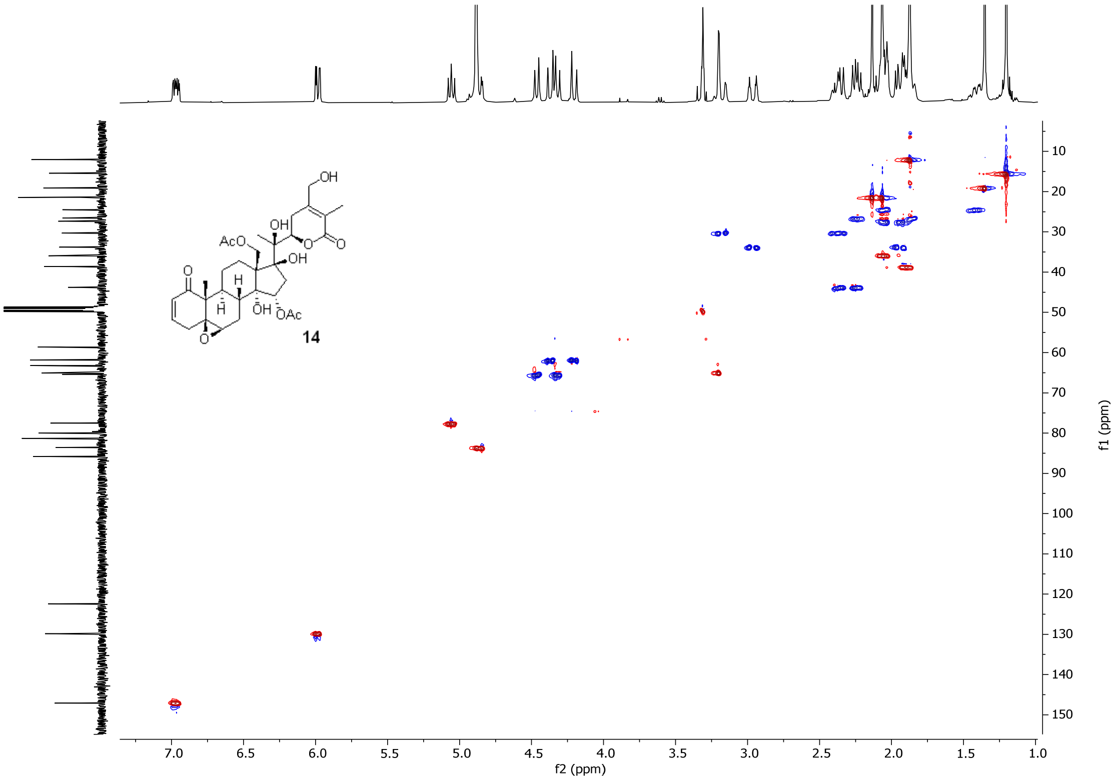

Figure S31. HMBC spectrum (400 MHz) of 15α-acetoxy-28-hydroxyphysachenolide C (**14**) in CD_3OD

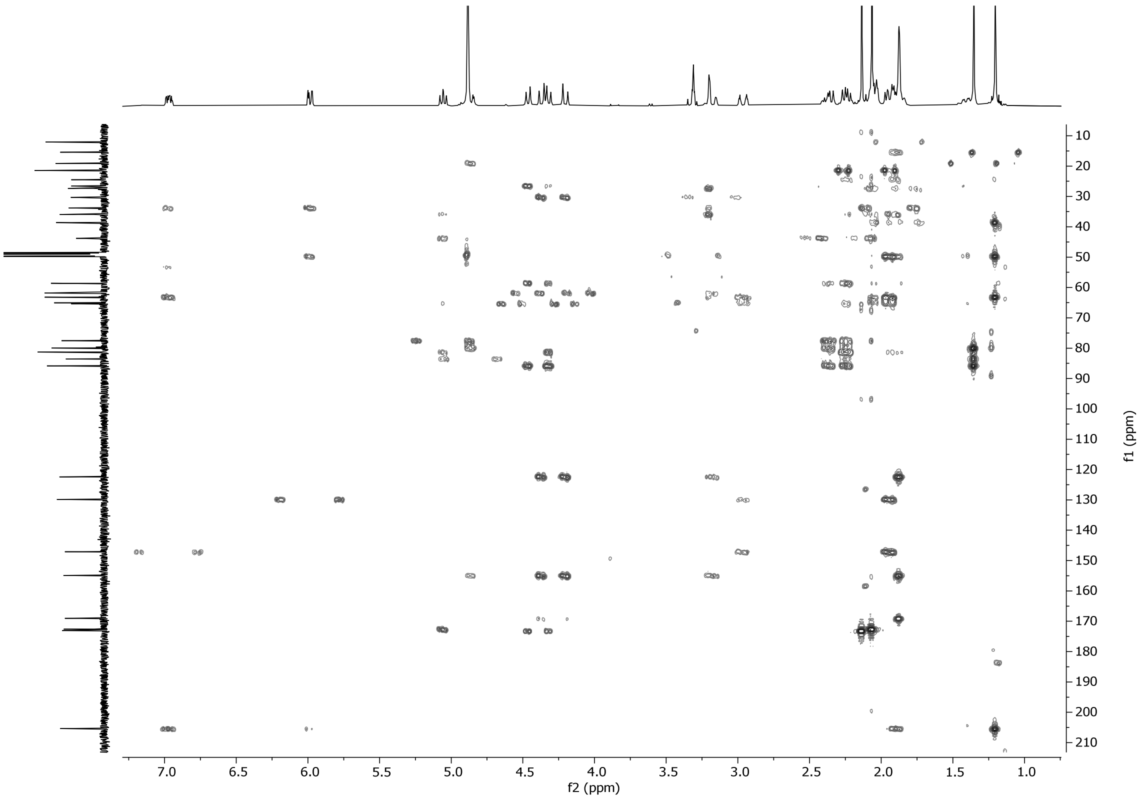

Figure S32. ^{1}H NMR spectrum (400 MHz) of 28-oxophysachenolide C (**15**) in $CDCl_3$

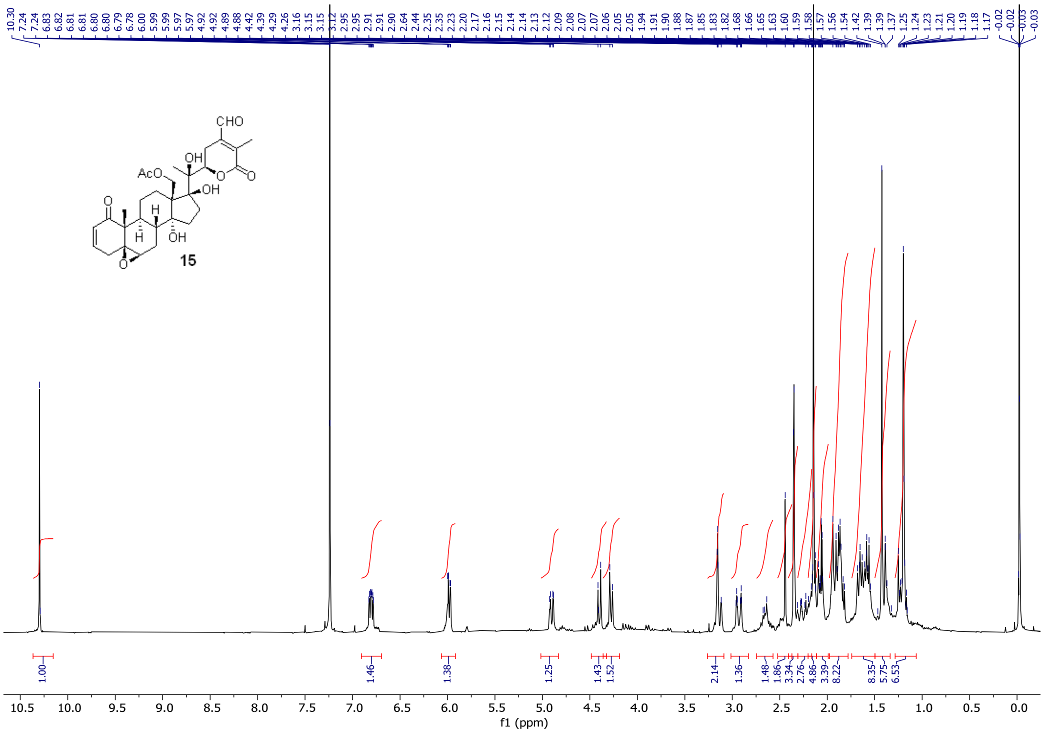

Figure S33. ^{13}C NMR spectrum (100 MHz) of 28-oxophysachenolide C (**15**) in $CDCl_3$

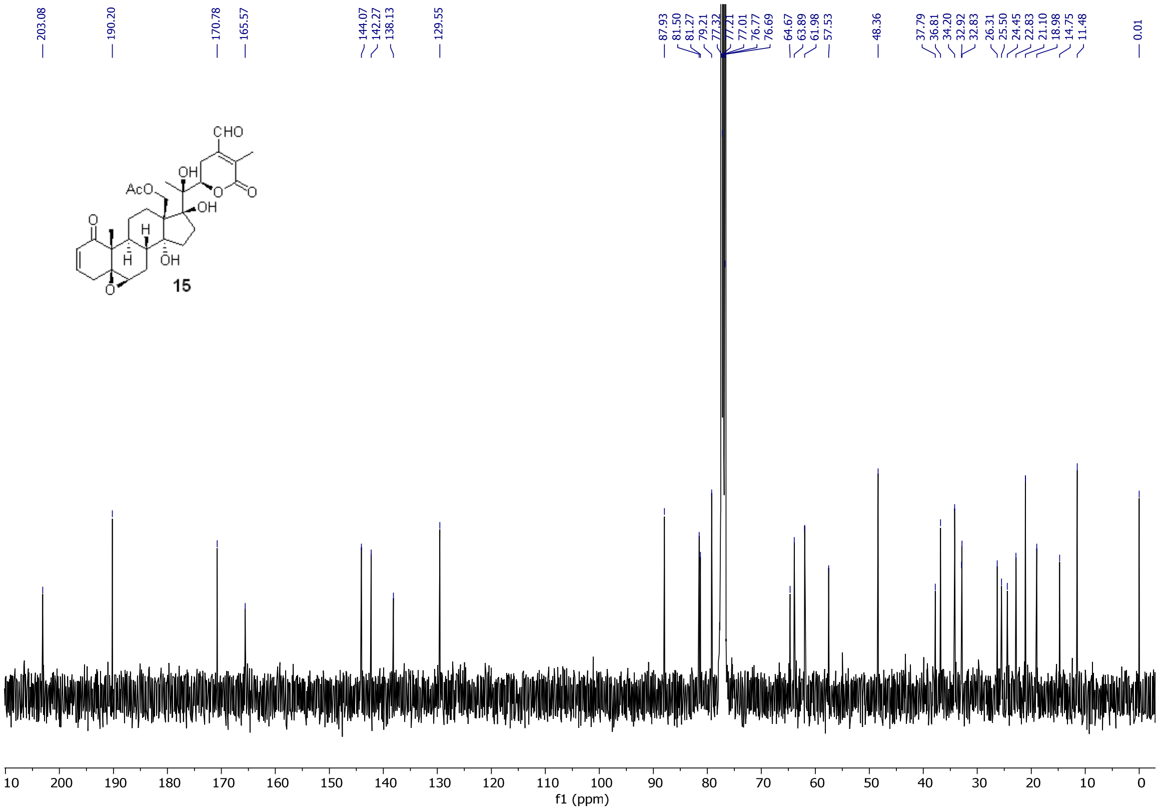

Figure S34. HSQC spectrum (400 MHz) of 28-oxophysachenolide C (**15**) in $CDCl_3$

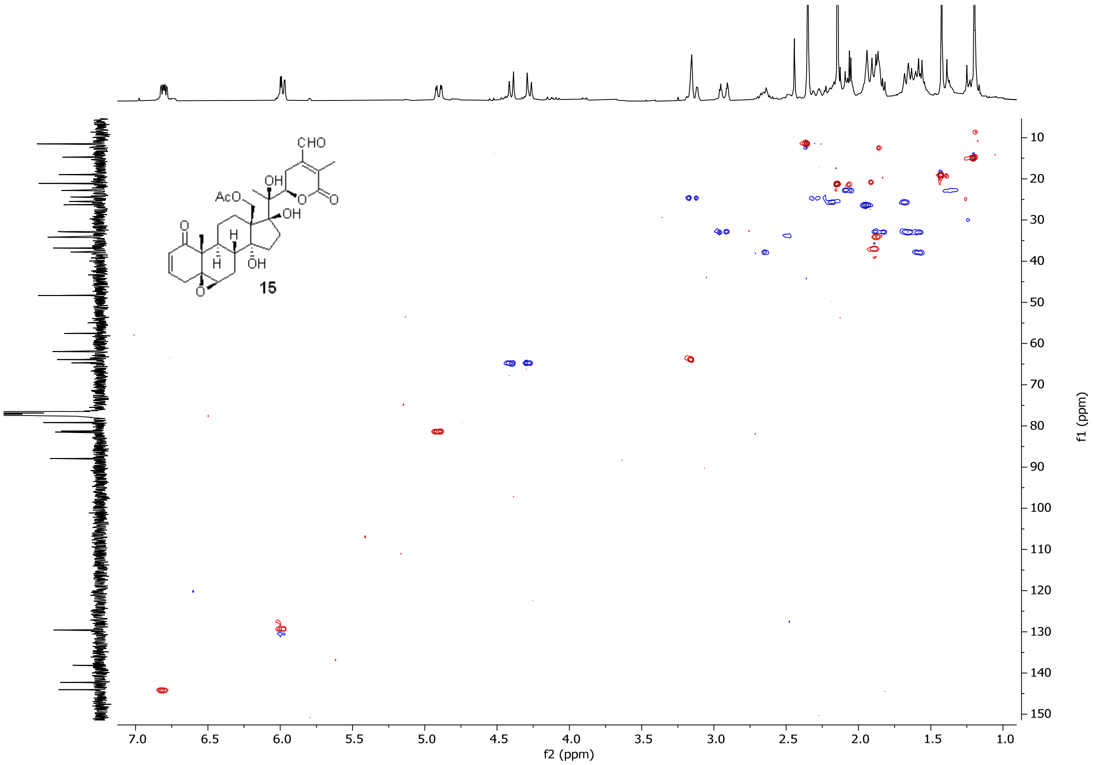

Figure S35. HMBC spectrum (400 MHz) of 28-oxophysachenolide C (**15**) in $CDCl_3$

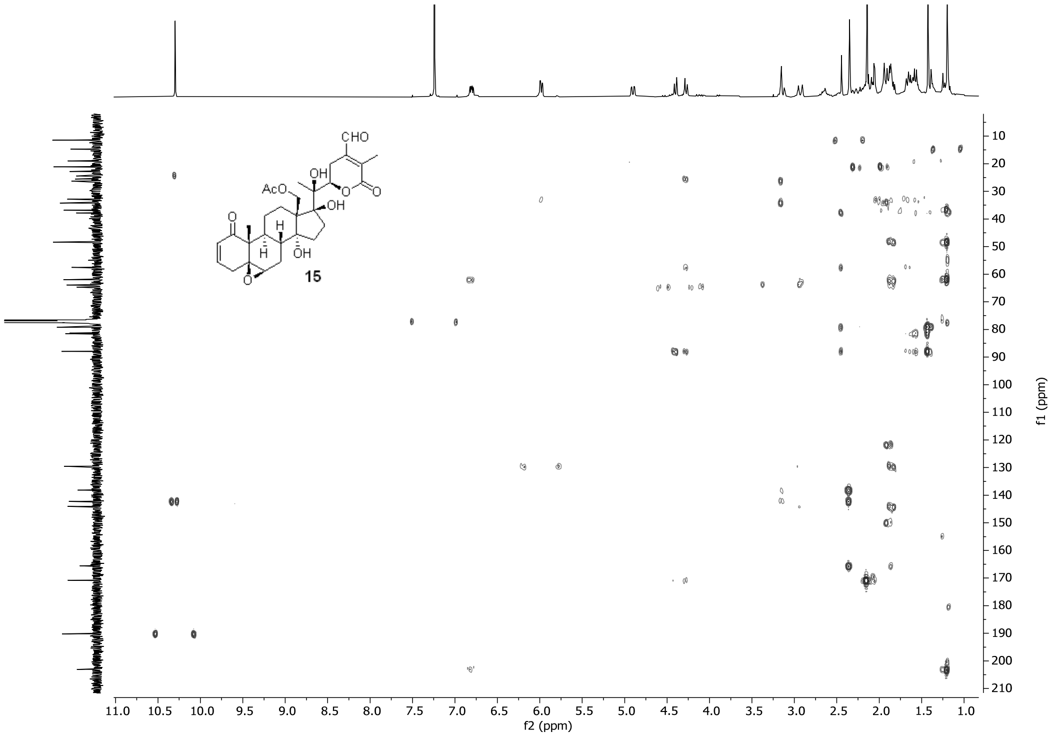

Figure S36. ^{1}H NMR spectrum (400 MHz) of physacoztolide M (**16**) in $CDCl_3/CD_3OD$ (100:1)

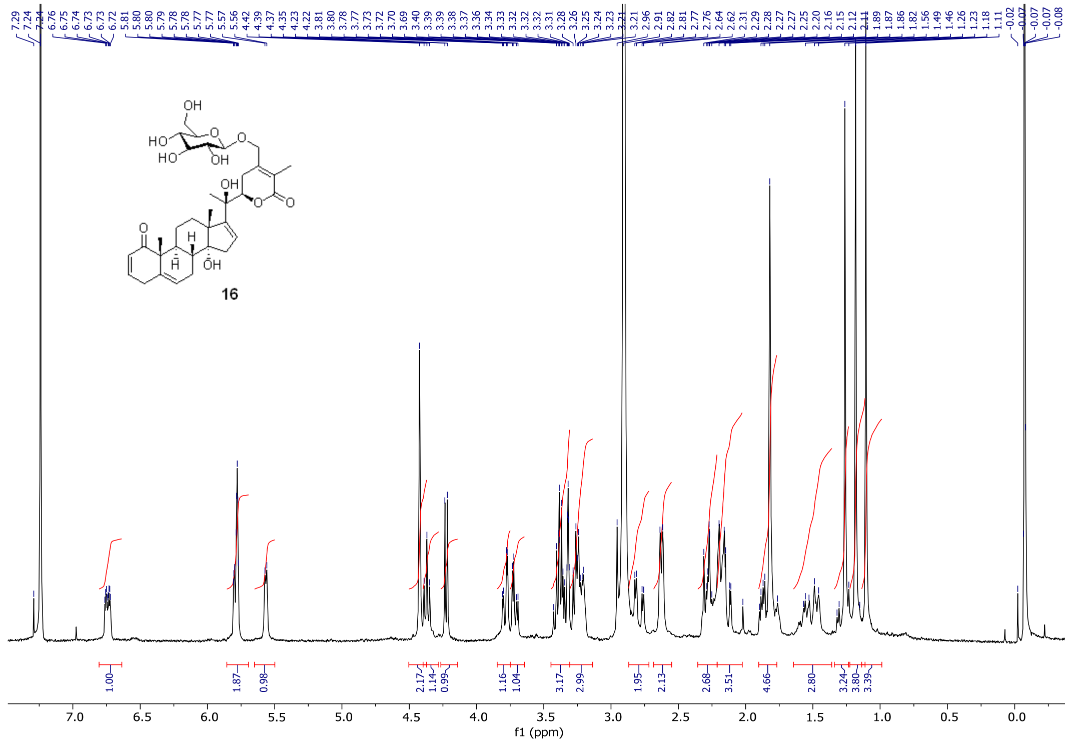

Figure S37. ^{13}C NMR spectrum (100 MHz) of physacoztolide M (**16**) in $CDCl_3/CD_3OD$ (100:1)

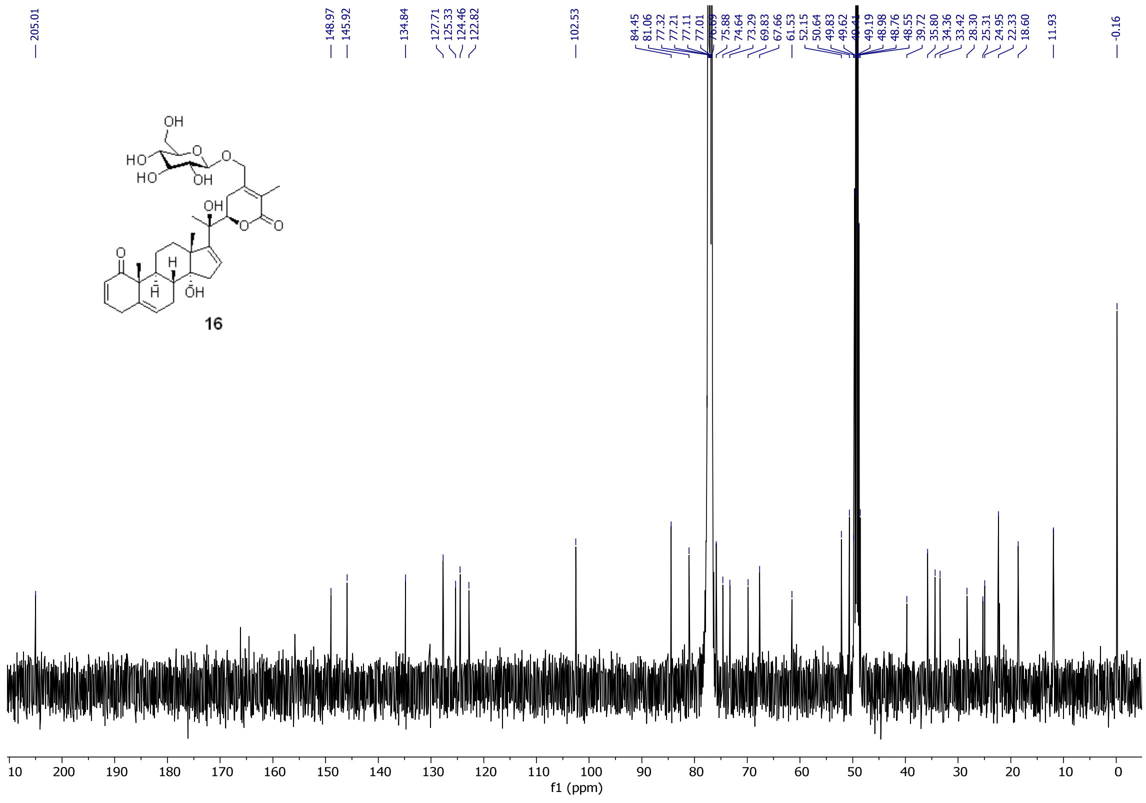

Figure S38. HSQC spectrum (400 MHz) of physacoztolide M (**16**) in $CDCl_3$/CD_3OD (100:1)

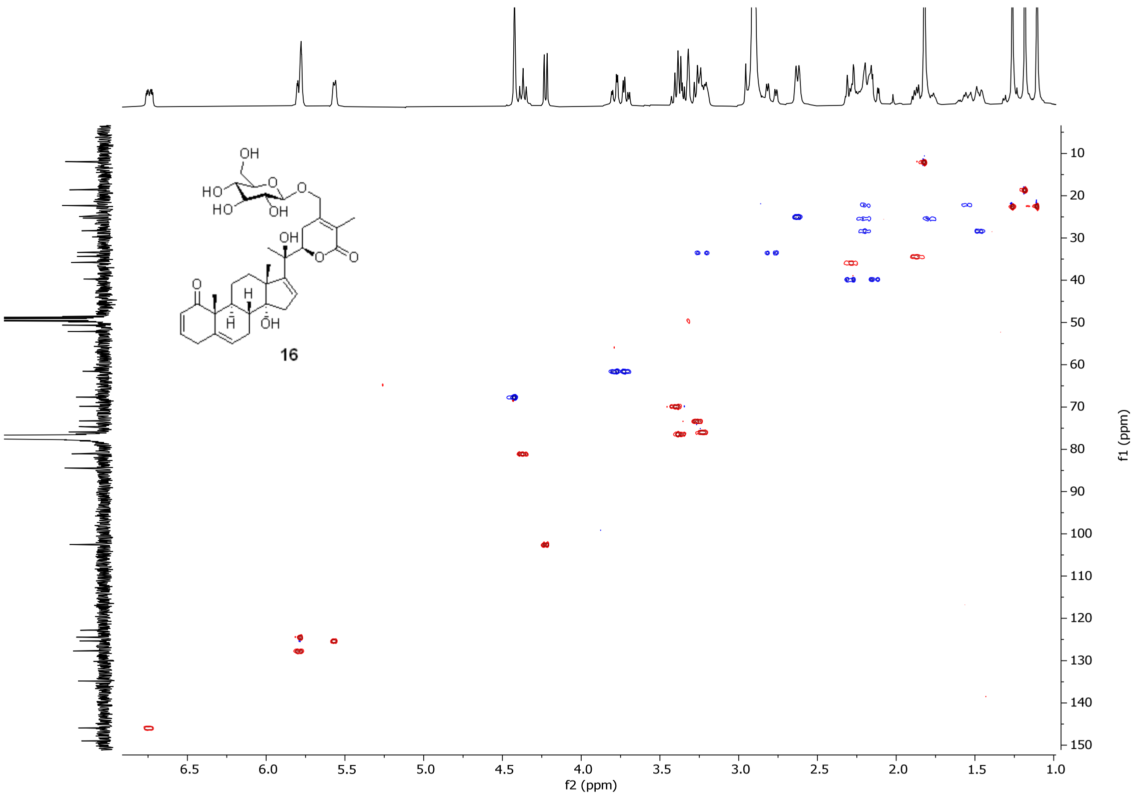

Figure S39. HMBC spectrum (400 MHz) of physacoztolide M (**16**) in $CDCl_3$/CD_3OD (100:1)

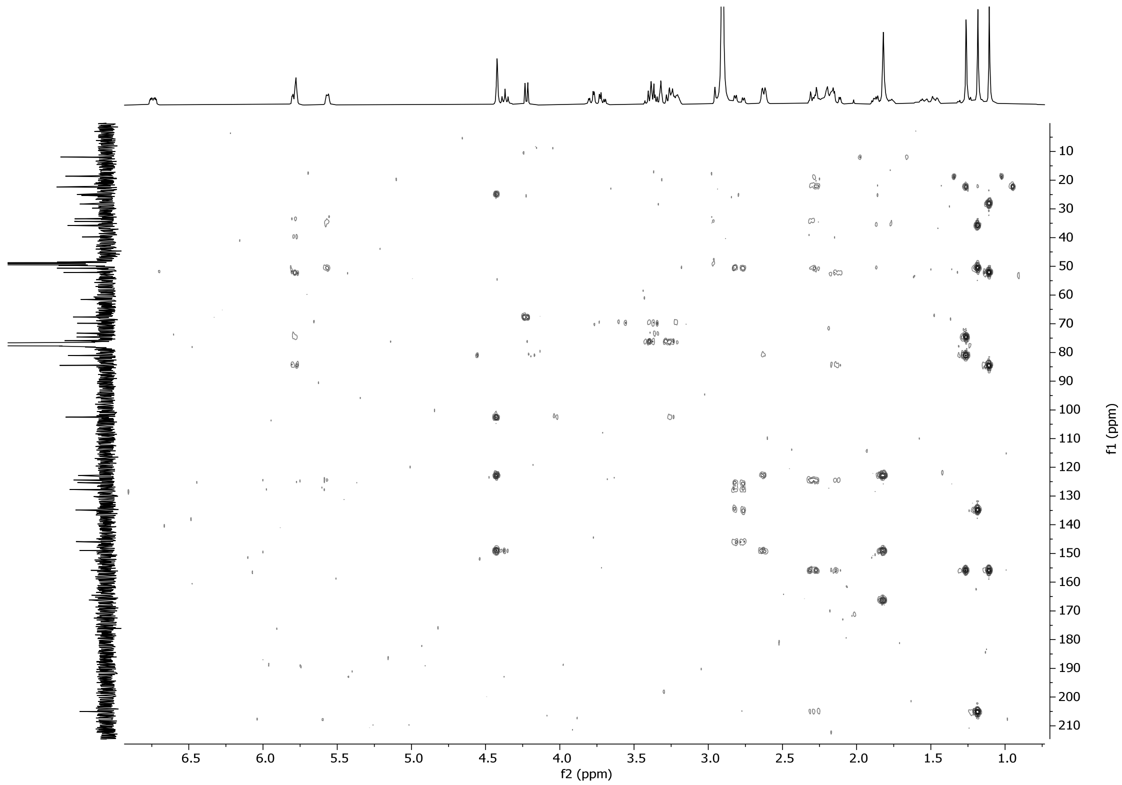

Figure S40. ¹H NMR spectrum (400 MHz) of 5α-chloro-6β-hydroxy-5,6-dihydrophysachenolide D (**17**) in $CDCl_3$

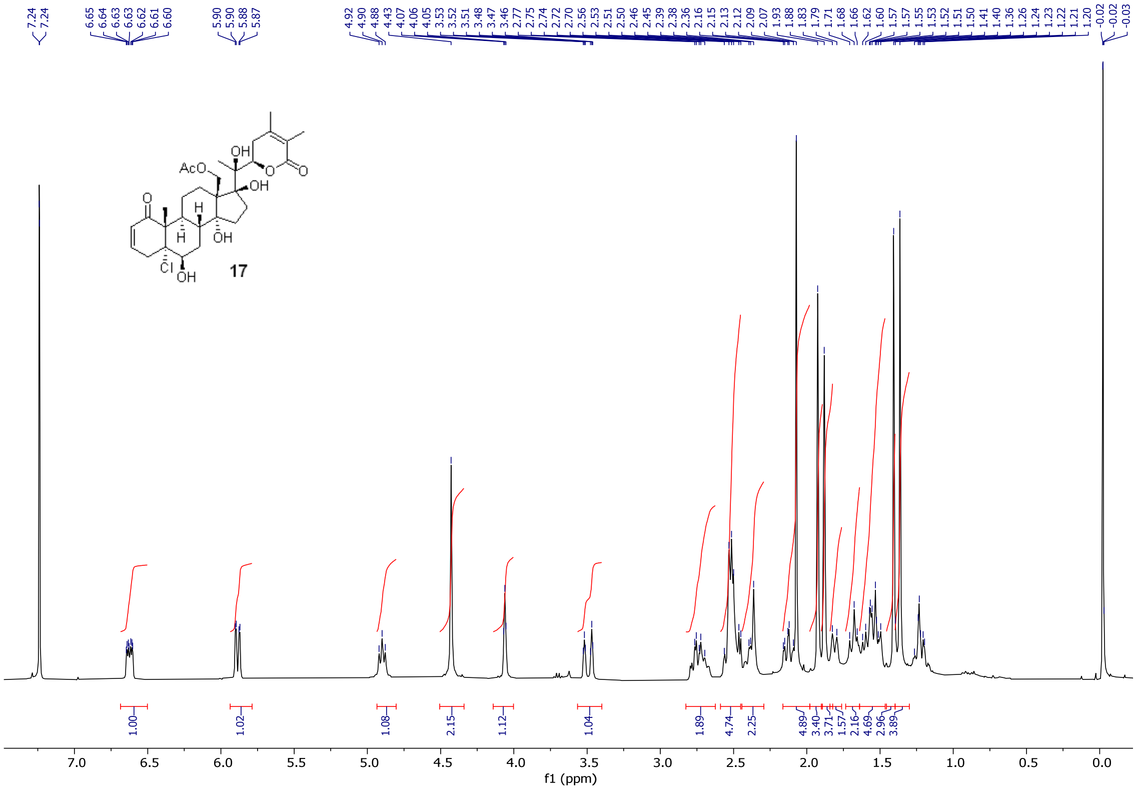

Figure S41. ¹³C NMR spectrum (100 MHz) of 5α-chloro-6β-hydroxy-5,6-dihydrophysachenolide D (**17**) in $CDCl_3$

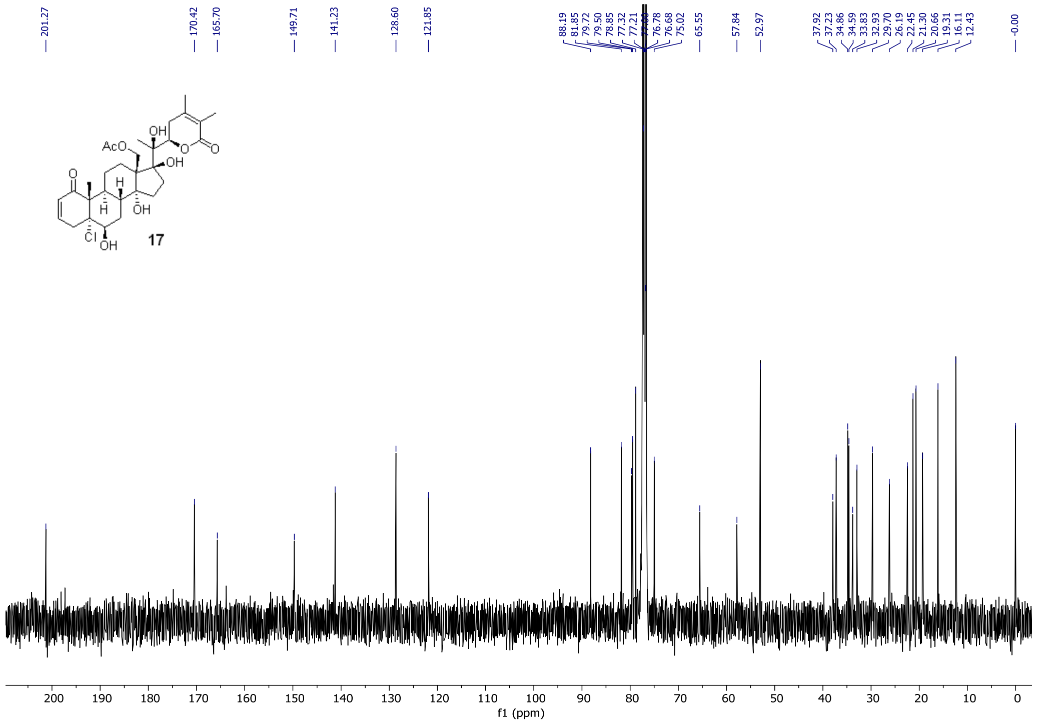

Figure S42. HSQC spectrum (400 MHz) of 5α-chloro-6β-hydroxy-5,6-dihydrophysachenolide D (**17**) in $CDCl_3$

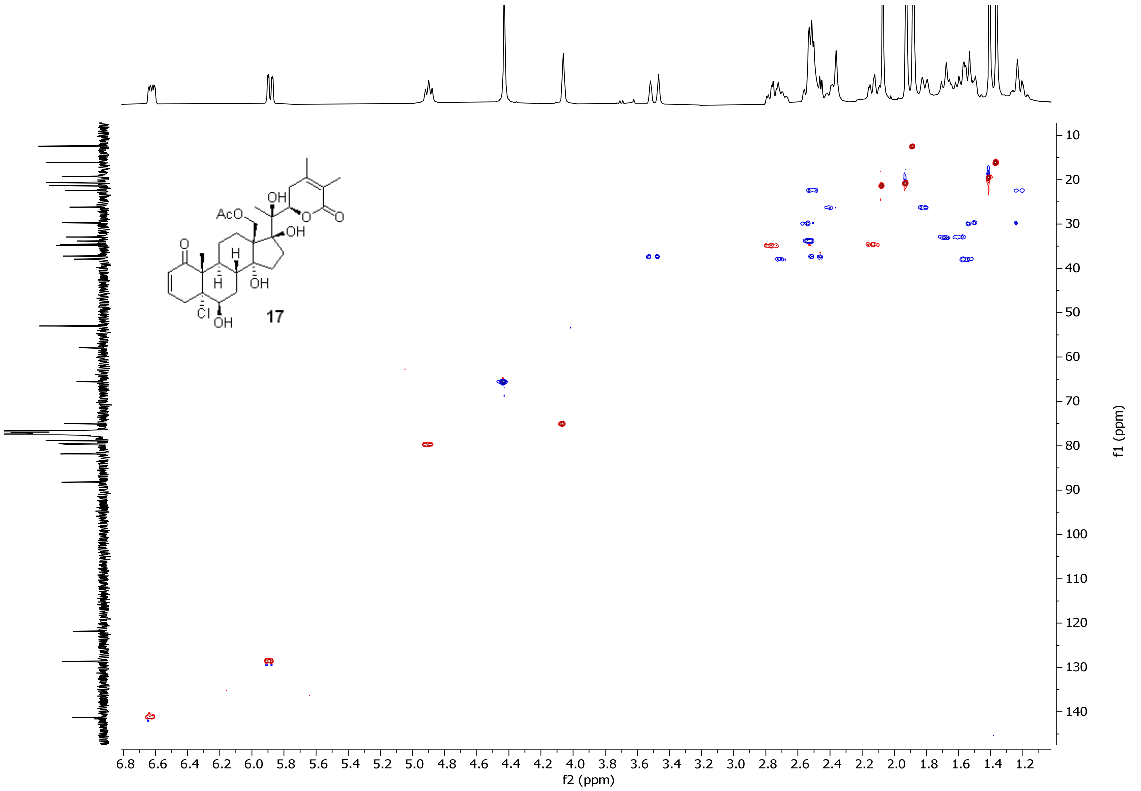

Figure S43. HMBC spectrum (400 MHz) of 5α-chloro-6β-hydroxy-5,6-dihydrophysachenolide D (**17**) in $CDCl_3$

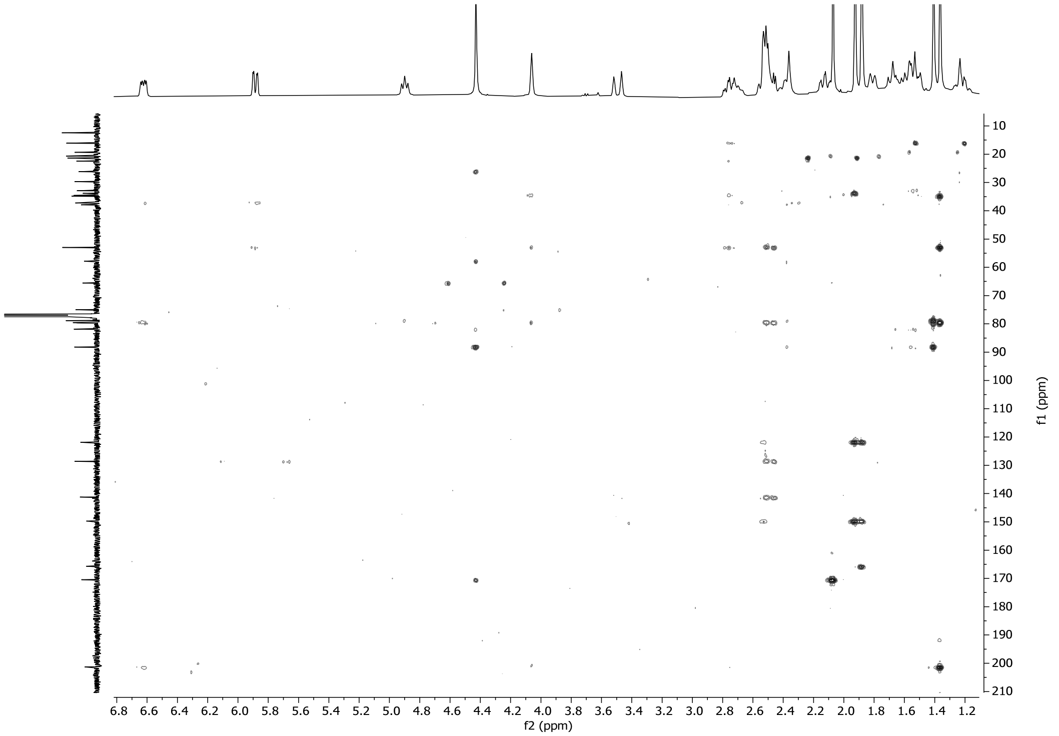

Figure S44. ^{1}H and 1D NOESY spectra (400 MHz) of 5*α*-chloro-6*β*-hydroxy-5,6-dihydrophysachenolide D (**17**) in $CDCl_3$

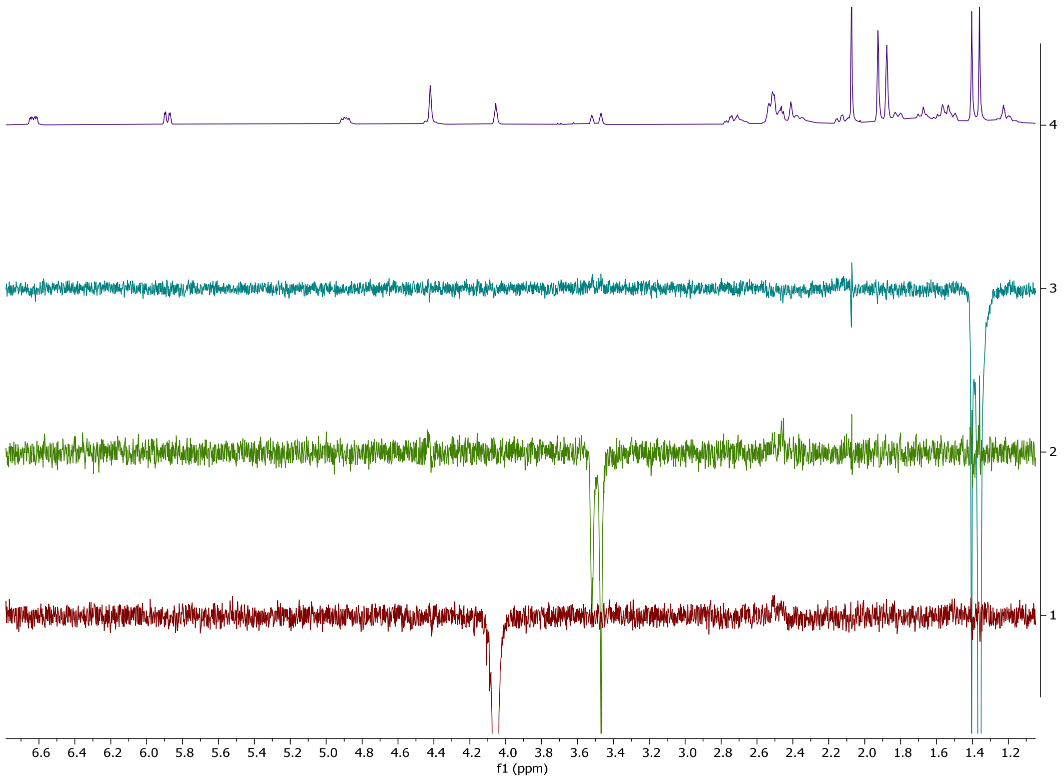

Figure S45. ^{1}H NMR spectrum (400 MHz) of 15*α*-acetoxy-5*α*-chloro-6*β*-hydroxyphysachenolide C (**18**) in $CDCl_3$

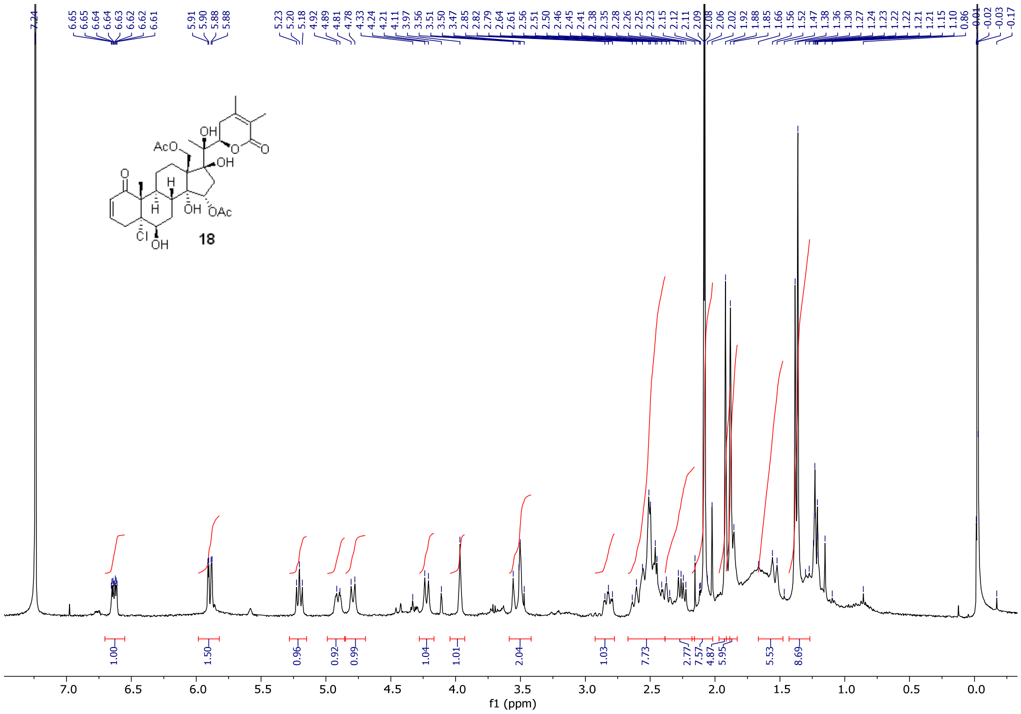

Figure S46. ^{13}C NMR spectrum (100 MHz) of 15*α*-acetoxy-5*α*-chloro-6*β*-hydroxy-5,6-dihydrophysachenolide D (**18**) in $CDCl_3$

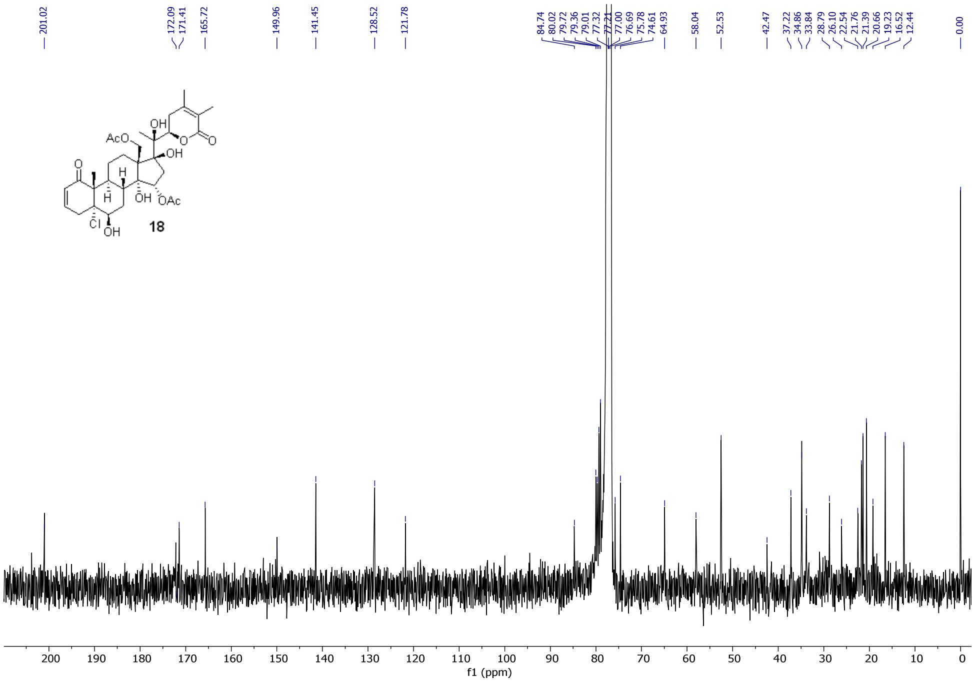

Figure S47. HSQC spectrum (400 MHz) of 15*α*-acetoxy-5*α*-chloro-6*β*-hydroxy-5,6-dihydrophysachenolide D (**18**) in $CDCl_3$

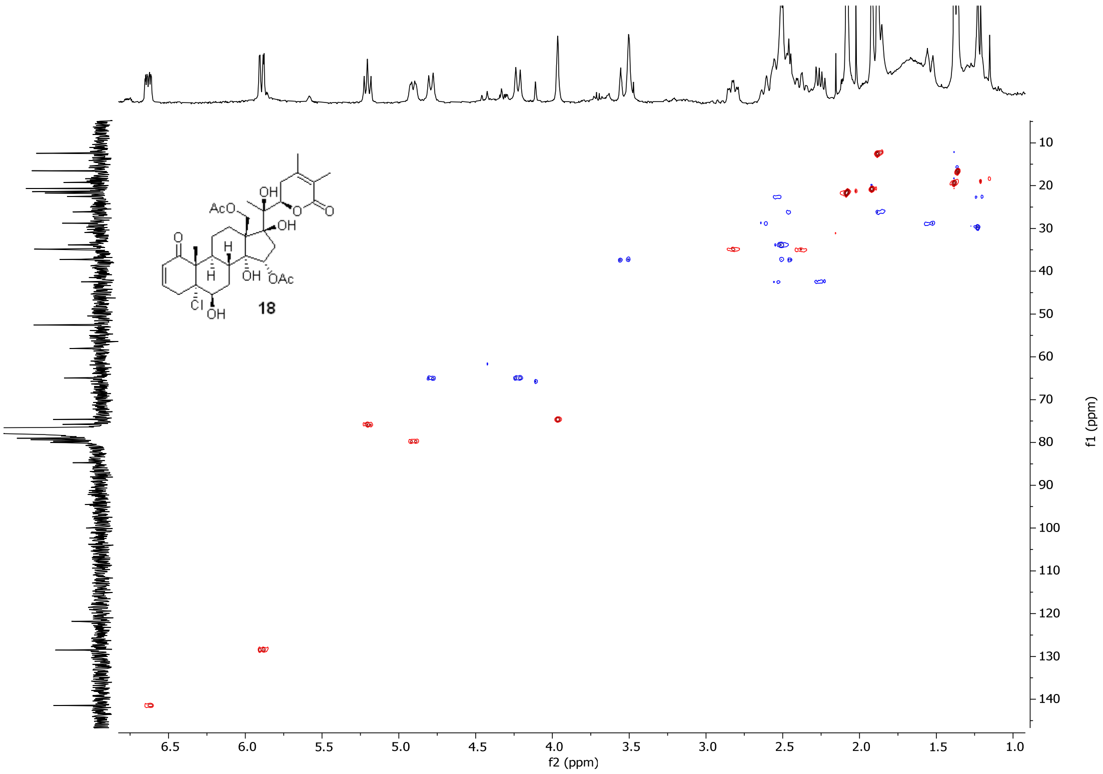

Figure S48. HMBC spectrum (400 MHz) of 15α-acetoxy-5α-chloro-6β-hydroxy-5,6-dihydrophysachenolide D (**18**) in $CDCl_3$

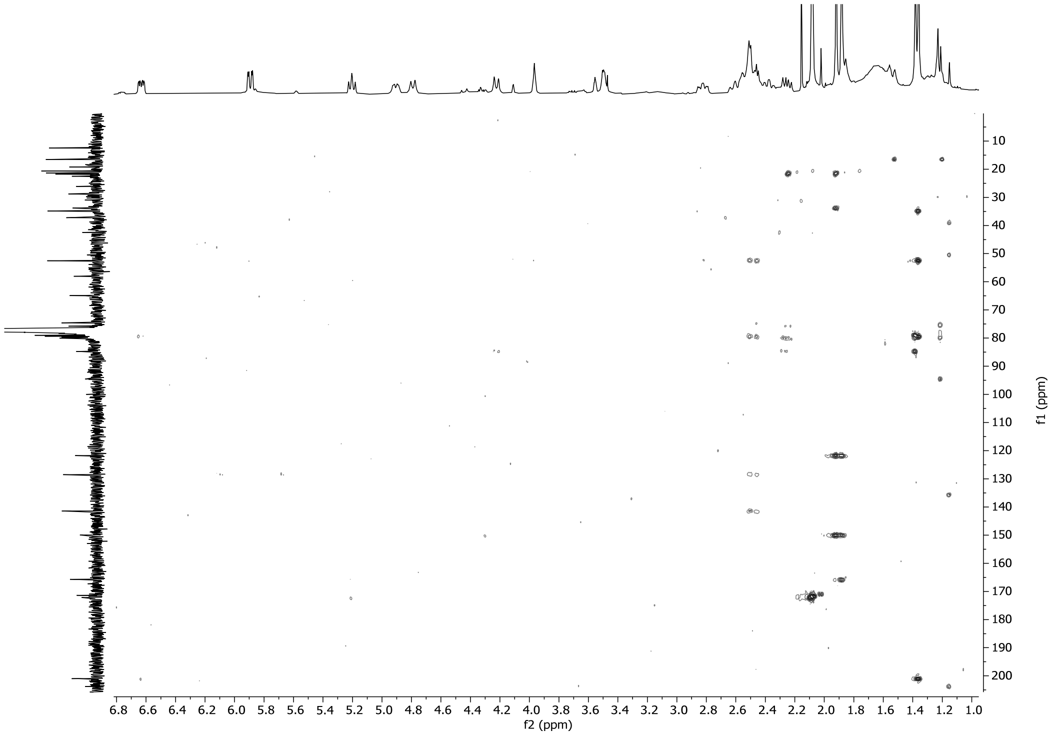

Figure S49. ^{1}H and 1D NOESY spectra (400 MHz) of 15α-acetoxy-5α-chloro-6β-hydroxy-5,6-dihydrophysachenolide D (**18**) in $CDCl_3$

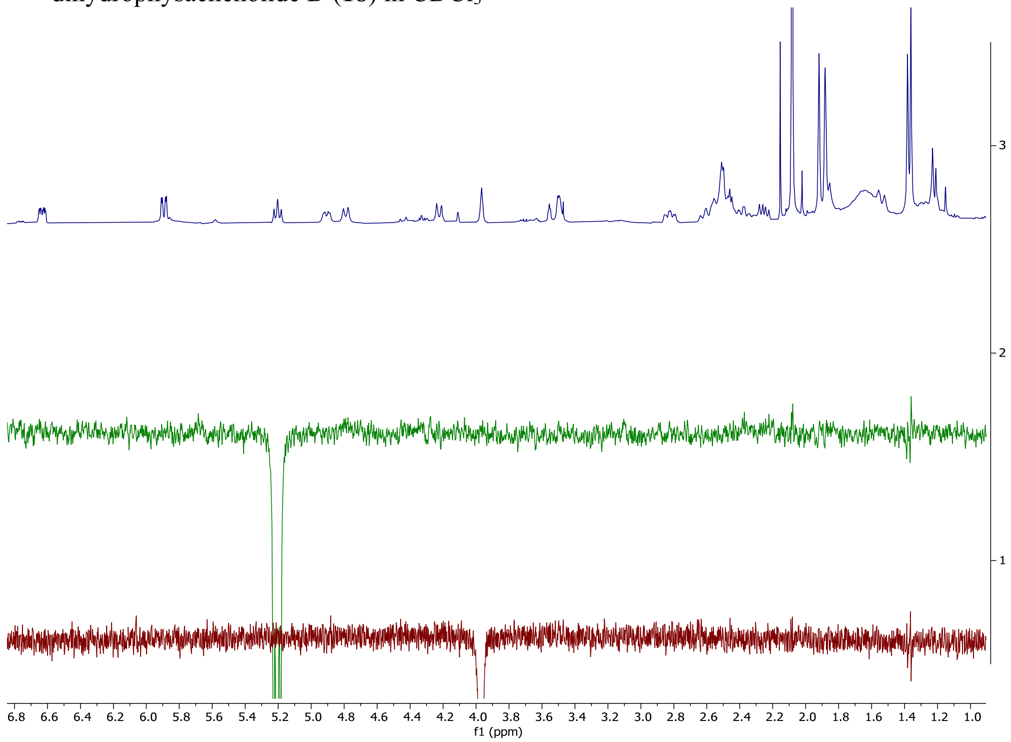

Figure S50. ^{1}H NMR spectrum (400 MHz) of 28-hydroxy-5α-chloro-6β-hydroxy-5,6-dihydrophysachenolide D (**19**) in $CDCl_3$

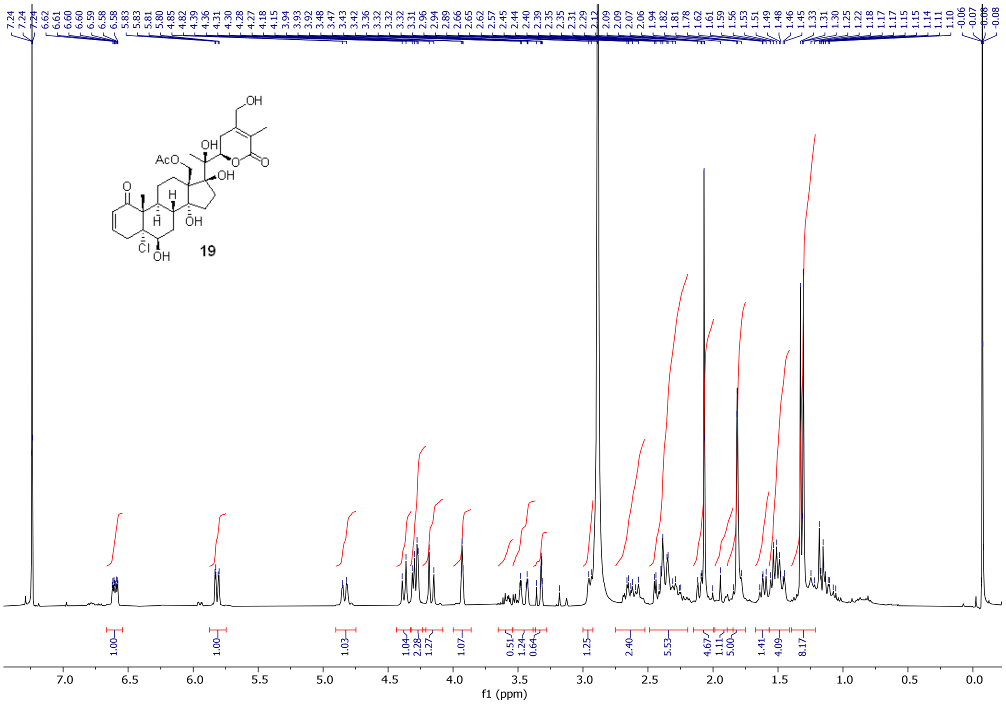

Figure S50a. ^{1}H NMR spectrum (400 MHz) of 28-hydroxy-5α-chloro-6β-hydroxy-5,6-dihydrophysachenolide D (**19**) in CD_3OD

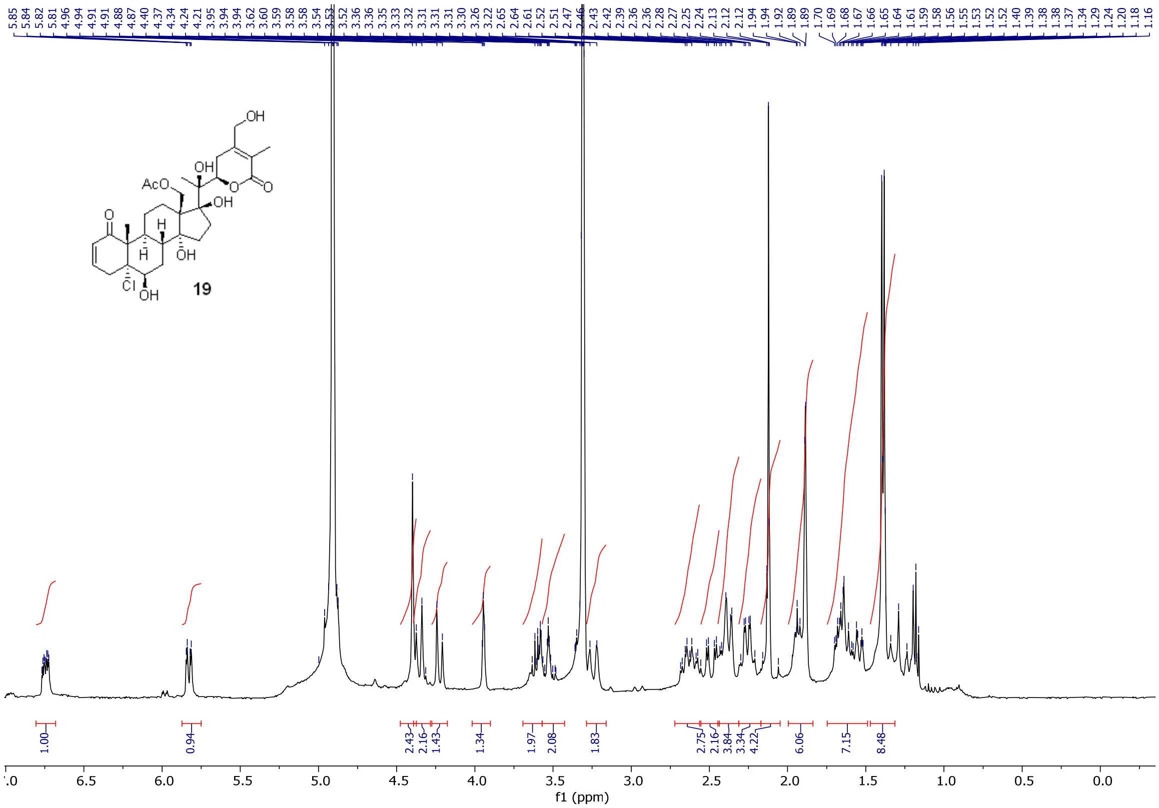

Figure S51. ^{13}C NMR spectrum (100 MHz) of 28-hydroxy-5α-chloro-6β-hydroxy-5,6-dihydrophysachenolide D (**19**) in $CDCl_3$

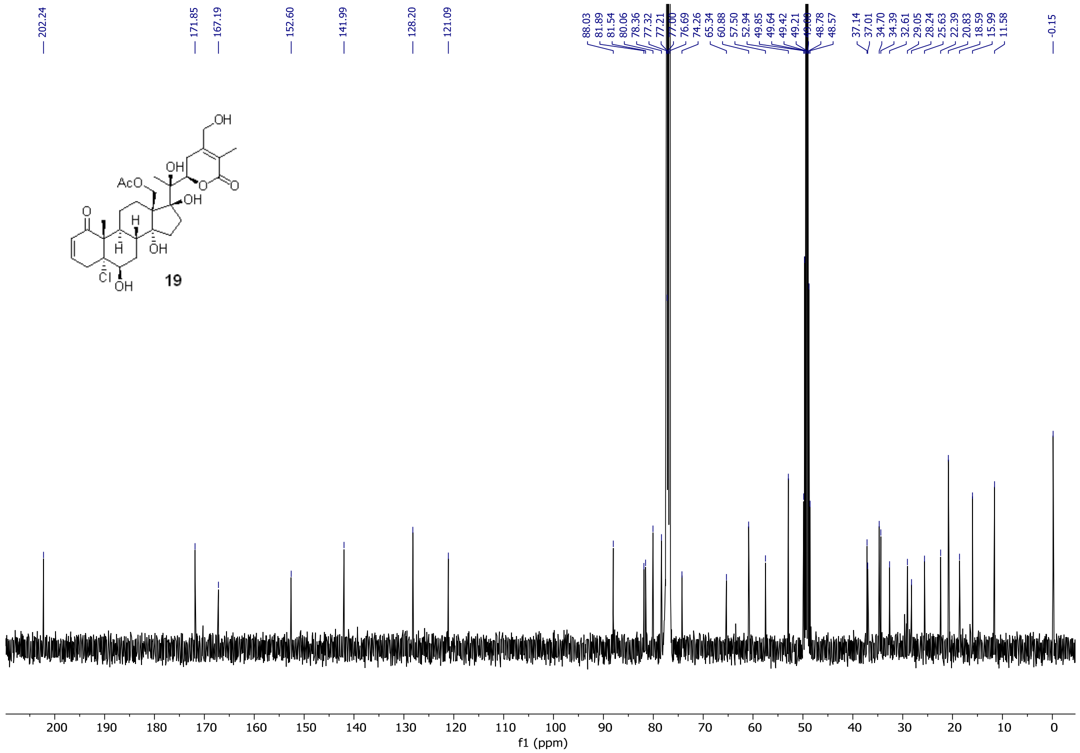

Figure S51a. ^{13}C NMR spectrum (100 MHz) of 28-hydroxy-5α-chloro-6β-hydroxy-5,6-dihydrophysachenolide D (**19**) in CD_3OD

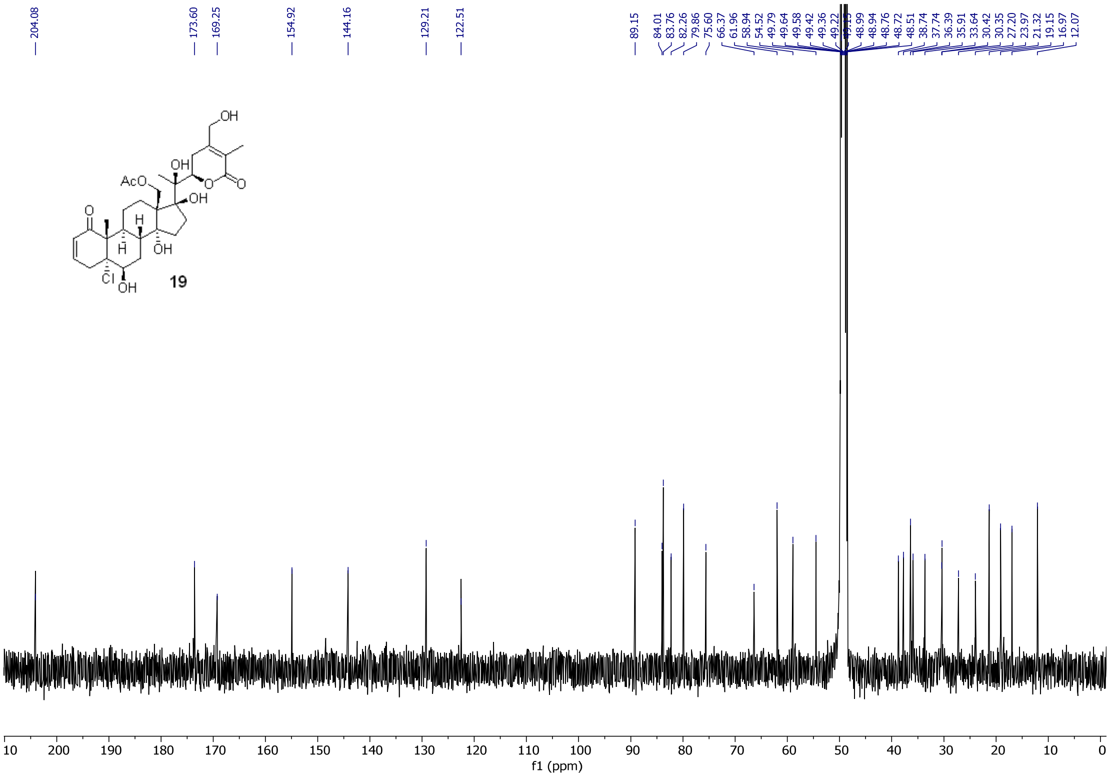

Figure S52. HSQC spectrum (400 MHz) of 28-hydroxy-5α-chloro-6β-hydroxy-5,6-dihydrophysachenolide D (**19**) in CD_3OD

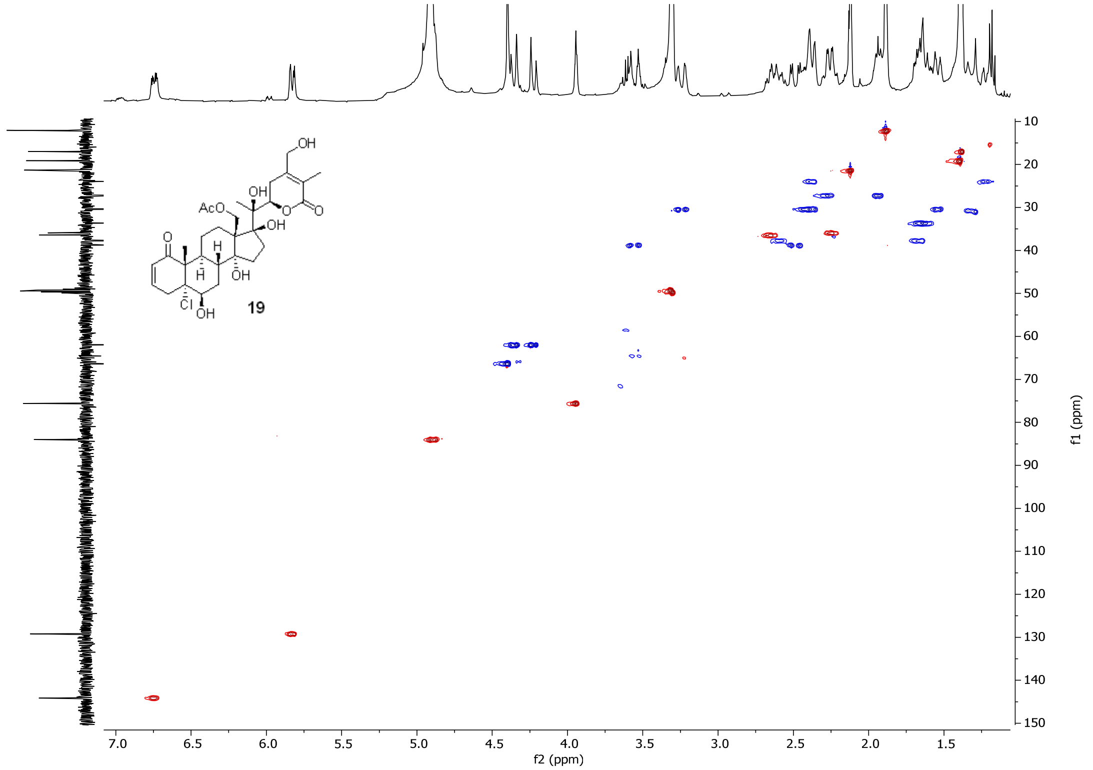

Figure S53. HMBC spectrum (400 MHz) of 28-hydroxy-5α-chloro-6β-hydroxy-5,6-dihydrophysachenolide D (**19**) in CD_3OD

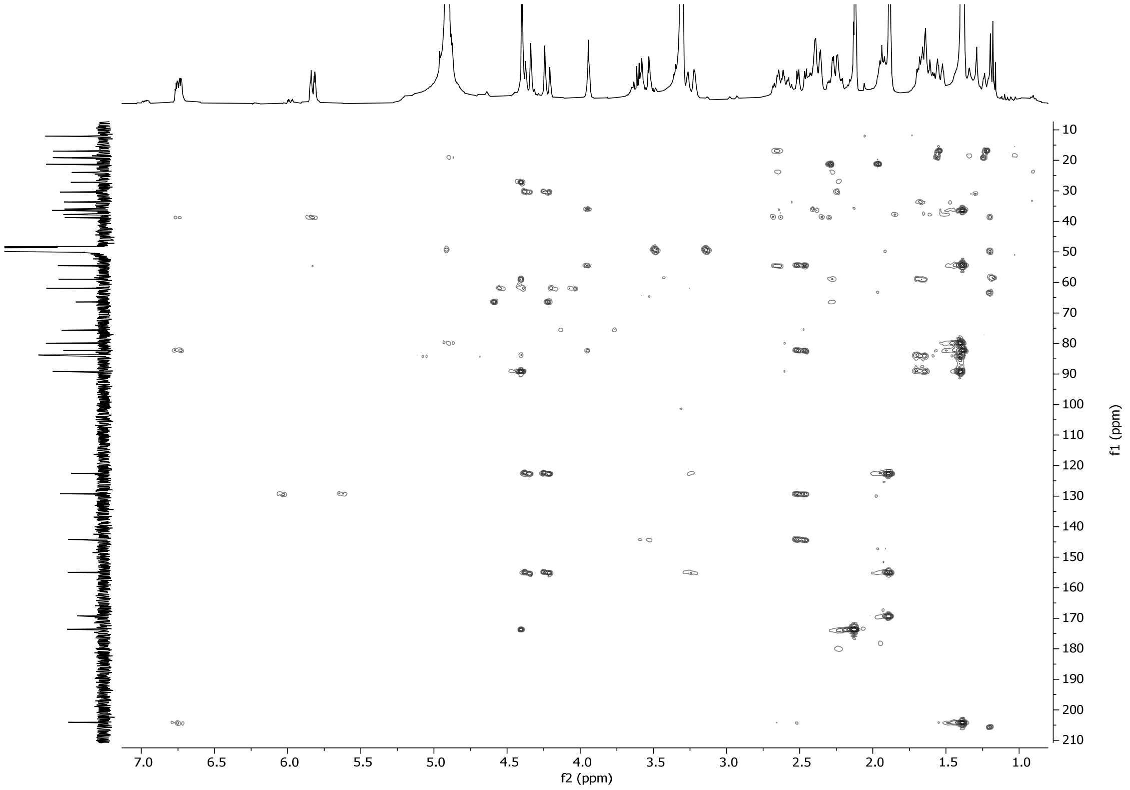

Figure S54. ^{1}H-^{1}H COSY spectrum (400 MHz) of 28-hydroxy-5α-chloro-6β-hydroxy-5,6-dihydrophysachenolide D (**19**) in CD_3OD

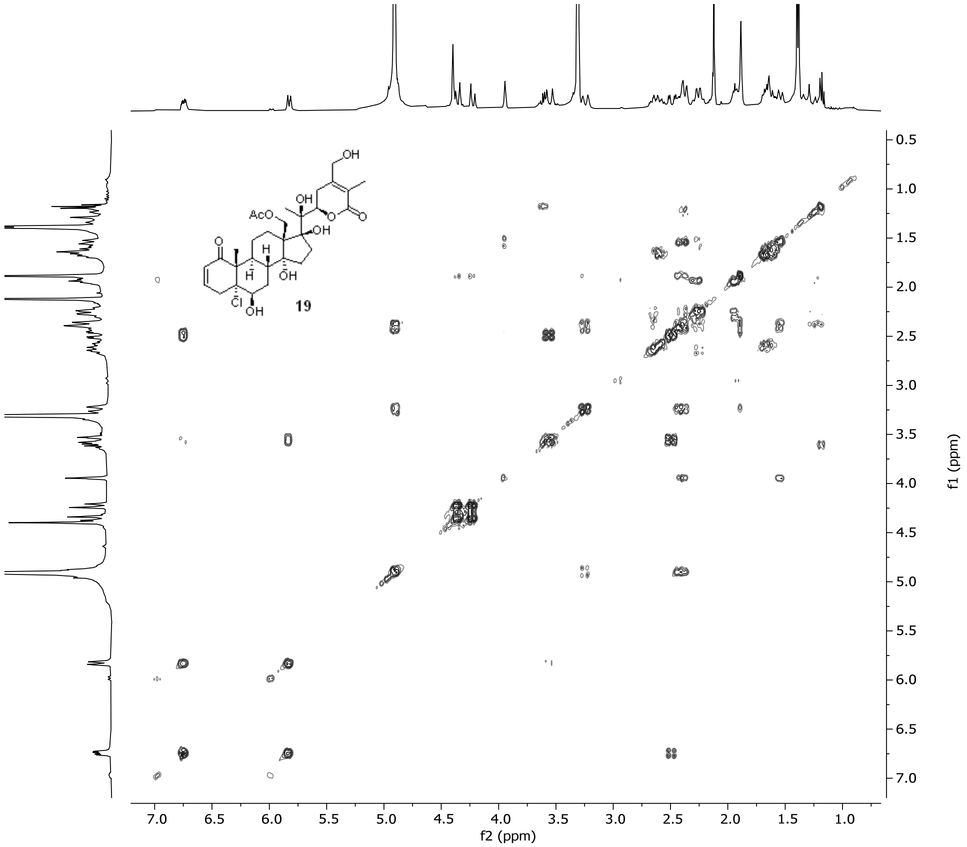

Figure S55. ^{1}H NMR spectrum (400 MHz) of physachenolide A-5-methyl ether (**20**) in $CDCl_3$

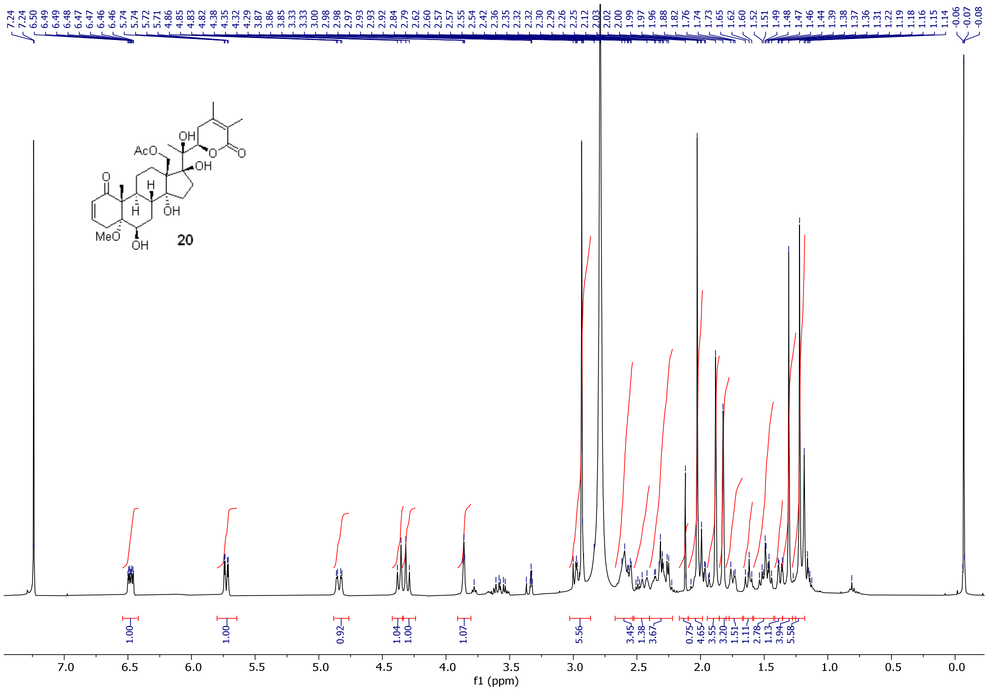

Figure S56. ^{13}C NMR spectrum (100 MHz) of physachenolide A-5-methyl ether (**20**) in $CDCl_3/CD_3OD$ (100:1)

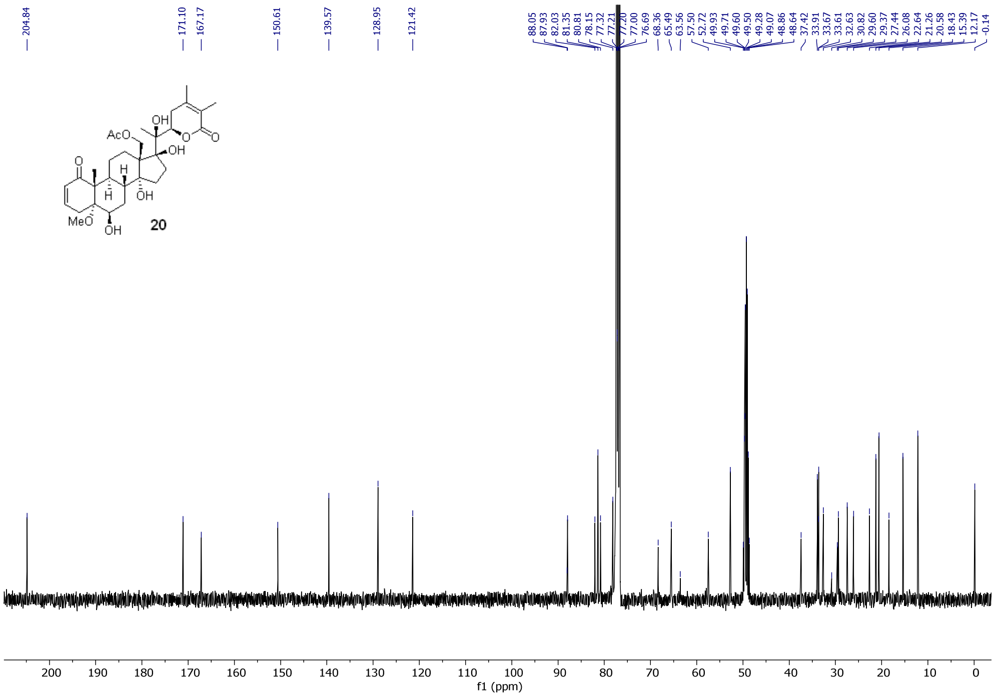

Figure S57. HSQC spectrum (400 MHz) of physachenolide A-5-methyl ether (**20**) in $CDCl_3/CD_3OD$ (100:1)

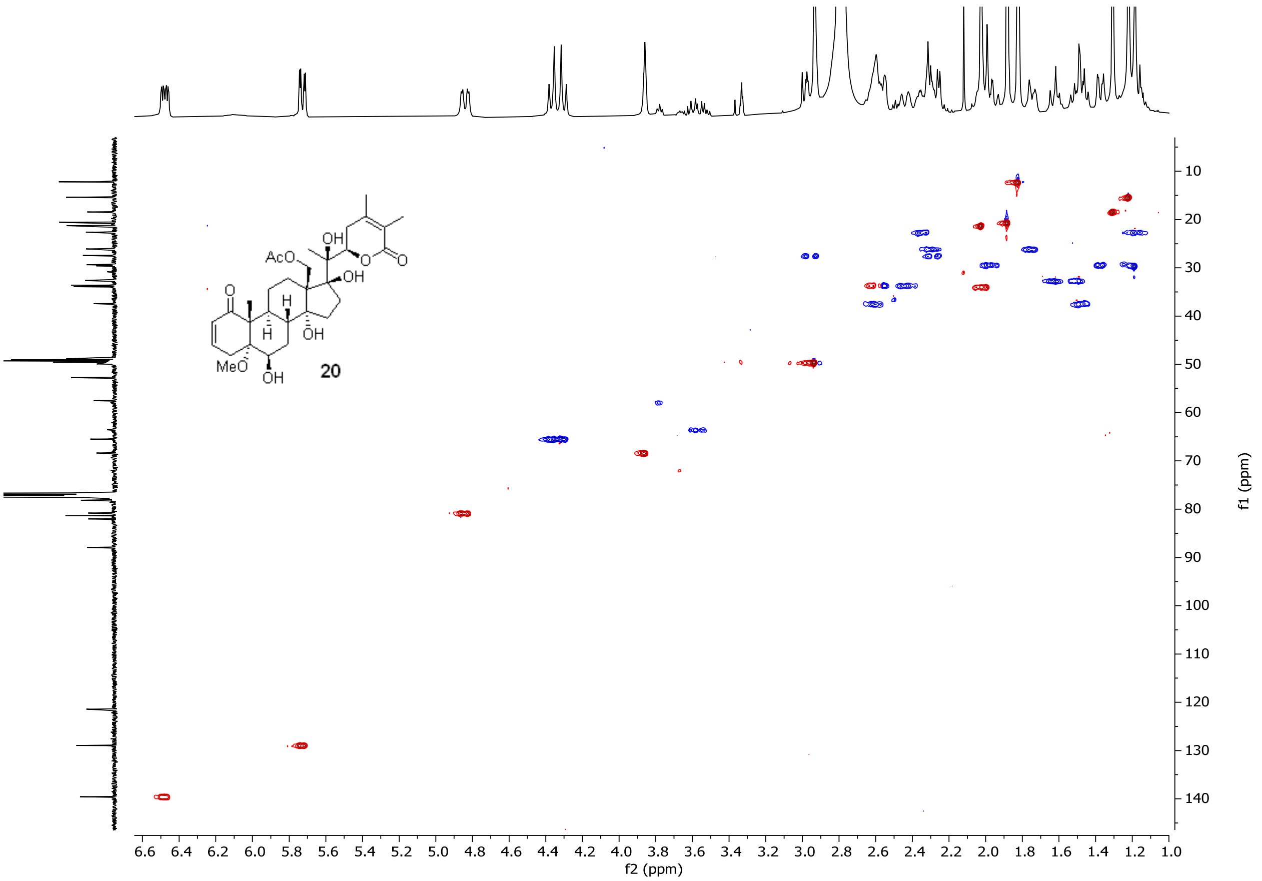

Figure S58. HMBC spectrum (400 MHz) of physachenolide A-5-methyl ether (**20**) in $CDCl_3/CD_3OD$ (100:1)

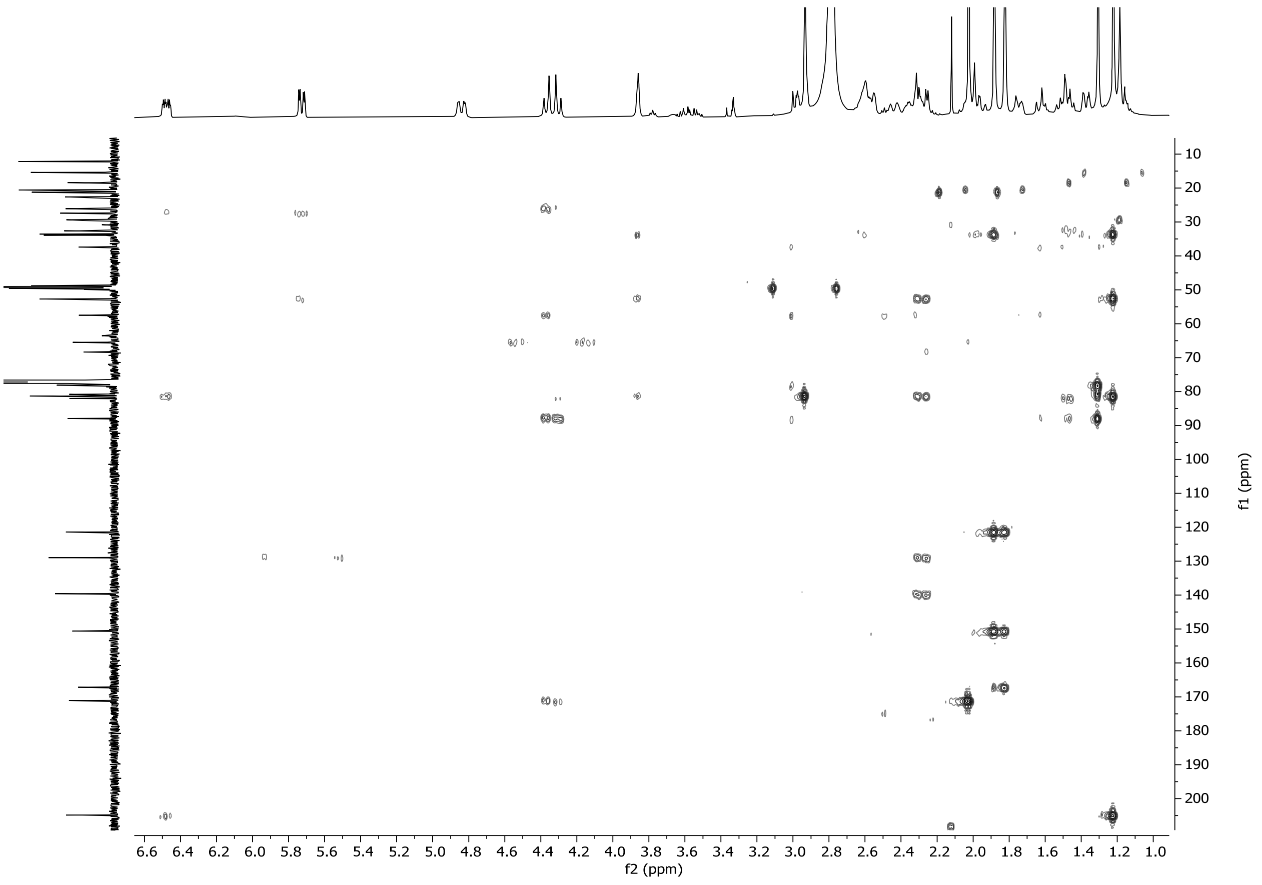

Figure S59. ECD spectra of withanolides **9**–**20**.

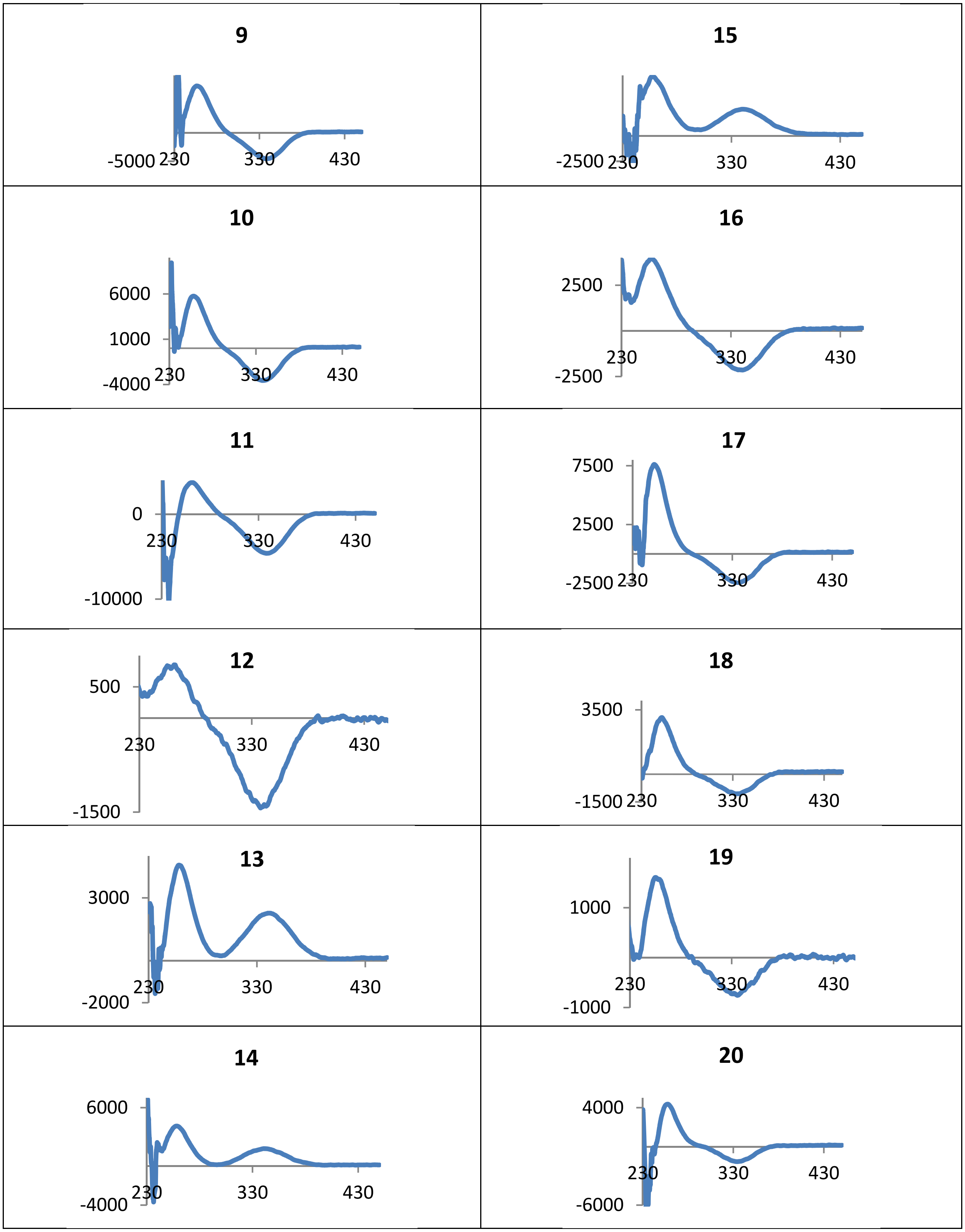

Figure S60. Key HMBC correlations (→) of **9–20** and Key ^{1}H-^{1}H COSY correlations (▬) of **11**, **12**, **19**, and **20**

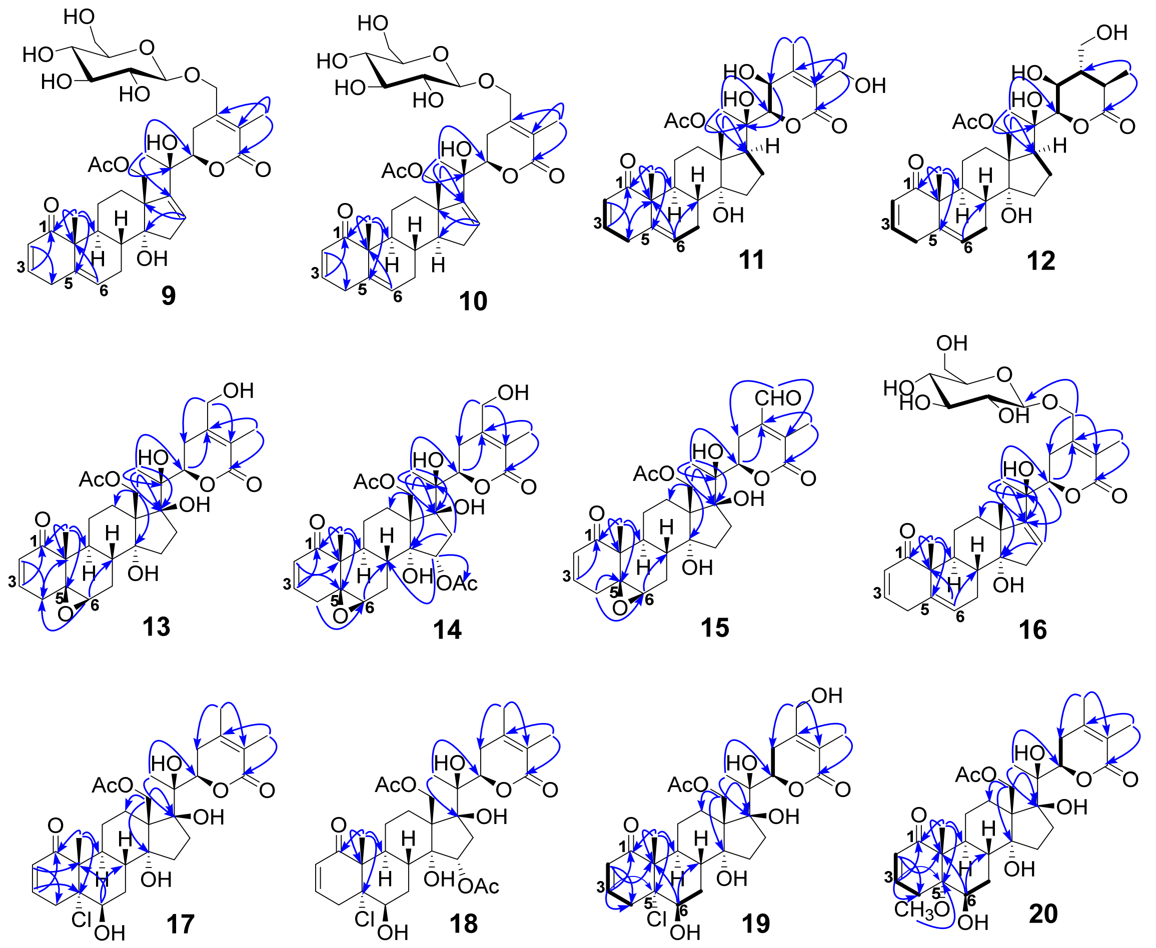

Figure S61. Key NOESY correlations (↔) of **11**, **12**, **17**, and **18**

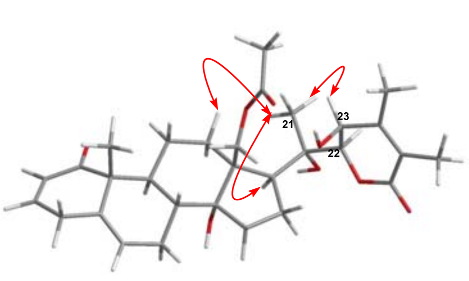

11

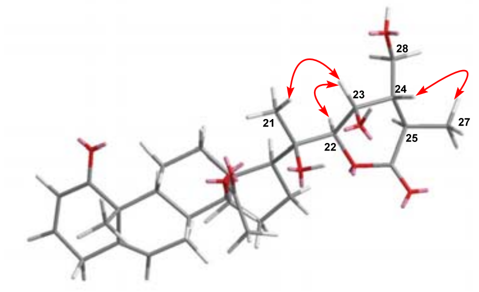

12

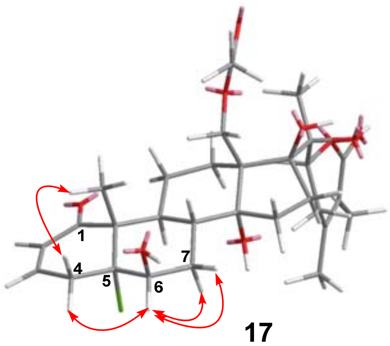

17

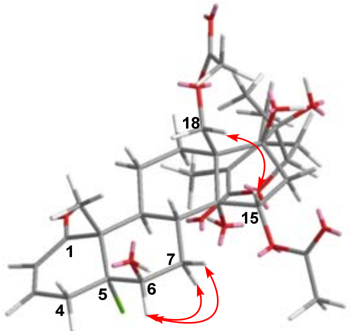

18

Figure S62. Investigation of products formed on exposure of physachenolide C (**8**) to mild acidic conditions by HPLC. **A**: HPLC trace of **8** in MeOH/H_2O. **B**: HPLC trace of withanolide **8** (0.05% w/v) exposed to 0.2 N HCl in MeOH/H_2O for 4 h at 25 °C showing its conversion to 5*α*-chloro-6*β*-hydroxy-5,6-dihydrophysachenolide D (**17**), 5α,6β-dihydroxy-5,6-dihydrophysachenolide D (**21**) and 5*α*-methoxy-6*β*-hydroxy-5,6-dihydrophysachenolide D (**20**), all of which were encountered in *P. coztomatl* extract. Identity of peaks due to **17**, **20**, and **21** were confirmed by the peak enhancement method.

Conditions used for HPLC analysis: 250 x 4.6 mm C-18 HPLC column, flow rate: 0.7 mL/min, Solvent: MeOH-H_2O gradient system by increasing MeOH from 40% to 100% in 30 min, detection: UV 230 nm.

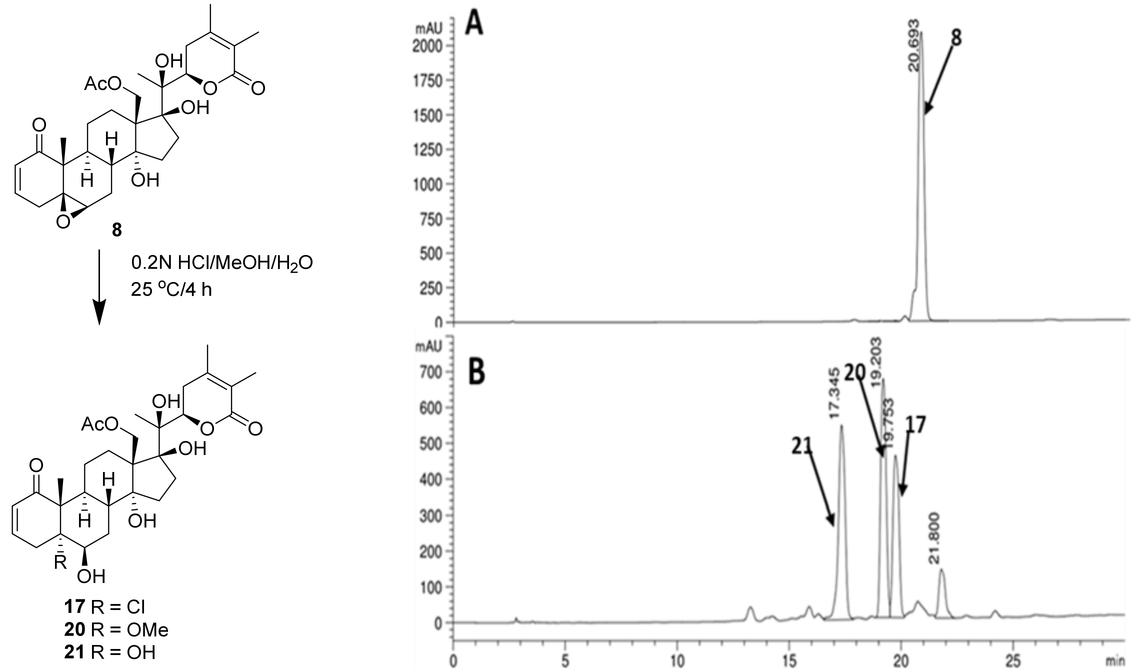

Supplement: Supplementary file 1 [file molecules-27-00909-s001.zip › molecules-1550100-supplementary.pdf]
